# Hindbrain boundaries as niches of neural progenitor and stem cells regulated by the extracellular matrix proteoglycan chondroitin sulphate

**DOI:** 10.1242/dev.201934

**Published:** 2024-02-13

**Authors:** Carmel Hutchings, Yarden Nuriel, Daniel Lazar, Ayelet Kohl, Elizabeth Muir, Olga Genin, Yuval Cinnamon, Hadar Benyamini, Yuval Nevo, Dalit Sela-Donenfeld

**Affiliations:** ^1^ Koret School of Veterinary Medicine, The Robert H. Smith Faculty of Agricultural, Food, and Environmental Sciences, The Hebrew University of Jerusalem, Rehovot 7610001, Israel; ^2^ Department of Physiology, Development and Neuroscience, University of Cambridge, Cambridge CB2 1TN, UK; ^3^ Agricultural Research Organization, Volcani Center, Department of Poultry and Aquaculture Science, Rishon LeTsiyon 7505101, Israel; ^4^ Info-CORE, Bioinformatics Unit of the I-CORE at the Hebrew University of Jerusalem, Jerusalem 9190401, Israel

**Keywords:** Hindbrain boundaries, Embryo, Neural stem cells, Progenitor cells, RNA-sequencing, Chondroitin-sulphate proteoglycan, Extracellular matrix

## Abstract

The interplay between neural progenitors and stem cells (NPSCs), and their extracellular matrix (ECM) is a crucial regulatory mechanism that determines their behavior. Nonetheless, how the ECM dictates the state of NPSCs remains elusive. The hindbrain is valuable to examine this relationship, as cells in the ventricular surface of hindbrain boundaries (HBs), which arise between any two neighboring rhombomeres, express the NPSC marker Sox2, while being surrounded with the membrane-bound ECM molecule chondroitin sulphate proteoglycan (CSPG), in chick and mouse embryos. CSPG expression was used to isolate HB Sox2^+^ cells for RNA-sequencing, revealing their distinguished molecular properties as typical NPSCs, which express known and newly identified genes relating to stem cells, cancer, the matrisome and cell cycle. In contrast, the CSPG^−^ non-HB cells, displayed clear neural-differentiation transcriptome. To address whether CSPG is significant for hindbrain development, its expression was manipulated *in vivo* and *in vitro*. CSPG manipulations shifted the stem versus differentiation state of HB cells, evident by their behavior and altered gene expression. These results provide further understanding of the uniqueness of hindbrain boundaries as repetitive pools of NPSCs in-between the rapidly growing rhombomeres, which rely on their microenvironment to maintain their undifferentiated state during development.

## Teaser

**Summary:** Transcriptomic analysis of hindbrain boundaries reveals that they harbor cells with neural progenitor and/or stem cell properties that rely on the local extracellular matrix to maintain their undifferentiated state.

## INTRODUCTION

A common theme during development is morphological organization into compartments comprising segregated cell populations (Morata and Lawrence, 1978). Those segments constitute the building blocks of the body plan, which dictate regional organization within the tissue. Segregation of cells into defined domains rely on their molecular properties that serve for recognition, as well as on the presence of boundaries, which assure cell separation as they organize, proliferate and differentiate (Batlle and Wilkinson, 2012; Dahmann et al., 2011; Irvine and Rauskolb, 2001; Kiecker and Lumsden, 2012). These boundaries were suggested to act as physical barriers to prevent neighboring cells from intermingling, as well as organizing centers that determine the fate of flanking cells by secretion of signals (Kiecker and Lumsden, 2005; Krumlauf and Wilkinson, 2021; Rubenstein et al., 1994). In the developing CNS, two well-defined boundary regions appear to function as organizing centers – the zona limitans intrathalamica (ZLI) and the midbrain-hindbrain boundary (MHB) – which orchestrate neuronal fates in the thalamus, midbrain and/or hindbrain through the secretion of Wnts, FGFs and/or SHH (Guinazu et al., 2007; Lim and Golden, 2007; Rhinn and Brand, 2001).

The embryonic hindbrain is an intriguing model for studying compartment boundaries (Moens and Prince, 2002). It undergoes a segmentation process along its rostro-caudal axis, resulting in transitory formation of seven to eight repetitive compartments, termed rhombomeres (Rhs). Each Rh has unique gene expression patterns promoting regional-specific fates, differentiation of neurons and production of distinct neural-crest streams (Frank and Sela-Donenfeld, 2019; Parker and Krumlauf, 2020). In between the Rhs, sharp domains arise, which are termed hindbrain boundaries (HBs). Remarkably, the HBs have been found to share specific genes and cellular characteristics; they have a fan-shaped morphology, are enriched in extracellular matrix (ECM) and have a reduced cell proliferation rate, as opposed to the neighboring Rhs (Heyman et al., 1995; Lumsden and Keynes, 1989). The fact that, unlike Rhs, the repetitive HBs share similar properties, together with their ability to regenerate once removed and their conserved formation in vertebrates, stresses their likely significance (Guthrie and Lumsden, 1991; Pujades, 2020; Wilkinson, 2021). Similar to other compartment boundaries, the HBs have been suggested to act as local organizing centers for the adjacent Rhs, achieved through secretion of various signaling molecules, such as FGF in chick or Wnt and semaphorins in zebrafish (Mahmood et al., 1995; Pujades, 2020; Riley et al., 2004; Sela-Donenfeld et al., 2009; Terriente et al., 2012; Weisinger et al., 2012).

We have previously found that Sox2, a neural progenitor and/or stem cell (NPSC) master gene known to regulate the self-renewal and multipotency of NPSCs (Lai et al., 2012; Sarkar and Hochedlinger, 2013), is enriched at the ventricular and/or subventricular regions of HBs in stage 16-18 chick embryo (HH16-18) (Peretz et al., 2016). We also found that these Sox2^+^ cells co-express other NPSC markers and constitute a slow-dividing group, which populate the boundary core, and an amplifying group localized near the HB-Rh interface. Furthermore, these Sox2^+^ HB (hereafter, HB-Sox2) cells were found to differentiate along the ventricular-mantle axis, but also to provide amplifying Sox2^+^ cells horizontally to Rhs, via cell division. Finally, Sox2 manipulations led to disorganized neurogenesis in the chick hindbrain. Based on these results, HBs have been proposed to act as pools of Sox2^+^ NPSCs aimed at providing progenitors to the intensely differentiating chick hindbrain. These findings were reinforced in the zebrafish hindbrain, where HBs were suggested to act as regions of self-renewing progenitors that later on provide differentiating neurons to the hindbrain (Hevia et al., 2022; Voltes et al., 2019). However, as opposed to the chick, Sox2 expression is not enriched in zebrafish HBs. Nevertheless, the corresponding evidence from chick and zebrafish hindbrains underlines a plausible new role for these unique domains. Yet the mechanisms governing the preservation of HB-Sox2 cells as NPSCs in between the Rhs are not known.

NPSCs in the sub-ventricular zone (SVZ) of the forebrain ventricles, or in the hippocampal dentate gyrus (DG) (Gates et al., 1995; Mira and Morante, 2020), are found in niches typically constructed with a network of ECM (Faissner and Reinhard, 2015; Kazanis and ffrench-Constant, 2011; Mercier, 2016; Su et al., 2019). A dedicated crosstalk has been suggested to exist between NPSCs and their milieu to regulate the behavior of the cell (Morrison and Spradling, 2008; Walma and Yamada, 2020), which is only partially understood. The hindbrain is a valuable system for illuminating this fundamental crosstalk, as we present here that the HBs of chick and mouse embryos display an intense accumulation of the ECM molecule chondroitin sulphate proteoglycan (CSPG). CSPG, which consists of several secreted or membrane-bound subtypes, is a major ECM component known to play crucial roles in CNS development and pathology (Carulli et al., 2005; Dyck and Karimi-Abdolrezaee, 2015). Here, we present evidence that a membranal-bound CSPG, DSD1, primarily surrounds the Sox2^+^ NPSCs in the HBs and prevents the cells from undergoing premature differentiation in both species. CSPG was also used to separate the HB-Sox2 cells for RNA-seq analysis, which strongly highlighted the properties of the HB-Sox2 cells as typical NPSCs, in contrast to the non-HB cells, which demonstrated a marked neural differentiation transcriptome profile. Altogether, we present further understanding on the molecular uniqueness of the HB cells as domains of NPSCs in the developing CNS and demonstrate their dependence on their ECM to stabilize their stem and/or progenitor-cell state.

## RESULTS

### Conserved expression of Sox2 and CSPG at hindbrain boundaries of chick and mouse embryos

**Fig. 1.**
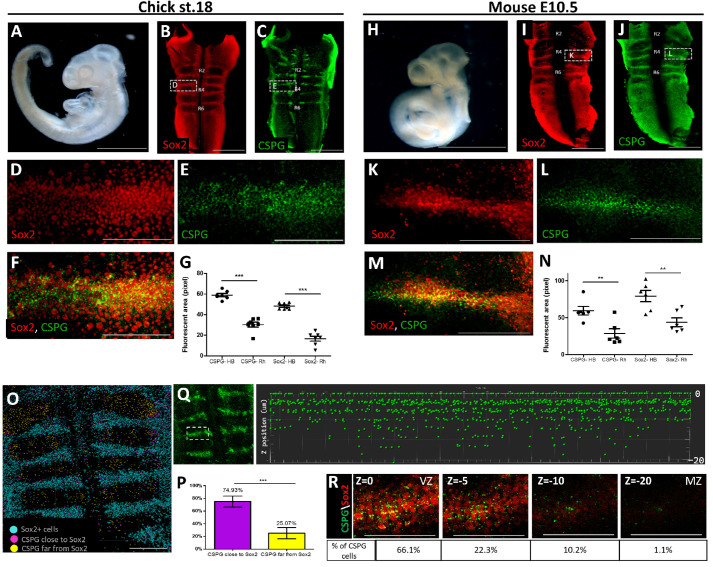
**Co-expression of Sox2 and CSPG in chick and mouse hindbrain boundaries.** (A,H) Images of HH18 chick and E10.5 mouse embryos. (B-F,I-M) Whole-mounted hindbrains immunostained for Sox2 or CSPG, or both. (G,N) Quantification of Sox2 and CSPG immunostaining in HBs and Rhs. Each dot represents an average of four HBs or Rhs in one embryo (*n*=7). (O,P) Representative proximity analysis of Sox2 and CSPG signals (*n*=7, distance ≤5 µm). (Q,R) Scatter plot (Q) and *z*-stack images (R) displaying CSPG distribution at the ventricular-mantle axis of a typical HB region. Area outlined in Q shows the region analyzed with *z*-distribution presented on the right. Data are mean±s.d. (two-tailed unpaired *t*-test). **P<0.005, ***P<0.0005. Scale bars: 1000 µm in A,H; 100 µm in B,C,I,J; 50 µm in D-F,K-M,R; 200 μm in O. HB, hindbrain boundary; Rh, rhombomere; VZ, ventricular zone; MZ, mantle zone.

HBs of HH16-18 chick embryos comprise Sox2^+^ cells while also being enriched with proteoglycans, in particular CSPG (Heyman et al., 1995; Weisinger et al., 2012). To determine whether these characteristics are avian specific or also preserved in mammals, we reviewed hindbrains of chick and mice embryos at equivalent stages (HH18 and E10.5, respectively) (Fig. 1A,H). In both species, the expression of Sox2 was notably higher in the nuclei of HB cells compared with Rhs (Fig. 1B,D,I,K). The use of a general antibody against CSPG revealed its co-localization with Sox2 at the HBs, exhibiting a membrane-bound pattern (Fig. 1C,E,F,J,L,M; Fig. S1A). Notably, longitudinal expression was also observed in the dorsal hindbrain. However, when an antibody specific to a membrane-bound CSPG subtype (DSD1; Faissner et al., 1994; Heyman et al., 1995) was employed, it was exclusively evident in the HBs (Fig. S1B), confirming that HB-Sox2^+^ cells express a membrane-bound CSPG. Quantification of the fluorescent area at the HBs confirmed that both proteins are enriched at mouse and chick HBs (Fig. 1G,N). Spatiotemporal analysis revealed that HB cells express both CSPG and Sox2 from HH16 to HH20, whereas rhombomeres show a notably lower incidence of co-expressing cells. In earlier stages (HH14), Sox2 exhibits a broader distribution across the hindbrain, in contrast to the notably fainter presence of CSPG (Fig. S2; Heyman et al., 1995; Peretz et al., 2016). To further validate the precise colocalization of Sox2 and CSPG, we executed a proximity analysis using IMARIS-3D software, by employing a module built to distinguish CSPG molecules expressed adjacent to Sox2 cells out of total CSPG signal detected (Fig. 1O). On average, nearly 75% of the identified CSPG molecules were found in proximity to Sox2^+^ cells (Fig. 1P, inclusion criteria- distance ≤5 µm; *n*=7). Further evaluation of the spatial co-expression of both proteins was analyzed in *z*-stacks along with a *z*-position scatter plot, showing both markers to be mostly expressed in the ventricular and sub-ventricular zones, rather than at the mantle layer of HBs (Fig. 1Q,R). The spatial arrangement of the Sox2^+^ CSPG^+^ cells was also analyzed by correlative light and scanning electron microscopy. In both species, the hindbrain topography was found to contain elevated ridges positioned in between submerged domains (Fig. S3A,B). Sox2 and CSPG accumulated at the ventricular surface of the elevated zones, verifying them as HBs. These results verify the colocalization of Sox2 and CSPG in the ventricular and/or subventricular layer of HBs, together with the unique topography of these domains in chick and mouse.

### Modifications of CSPG levels affect the differentiated state in the hindbrain

**Fig. 2.**
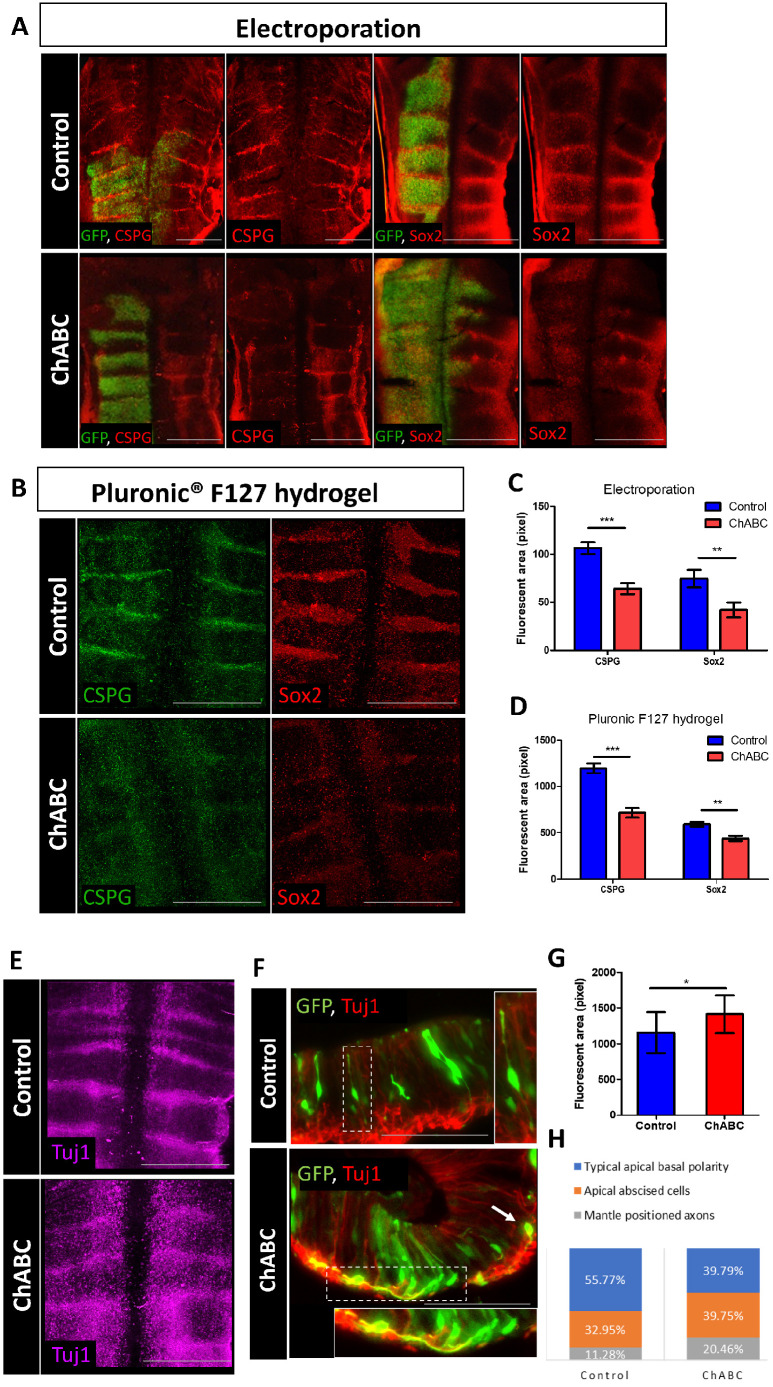
**CSPG loss alters the expression of Sox2 and Tuj1 *in vivo*.** (A,B,E) Flat-mount views of HH18 chick hindbrains electroporated with control-GFP or ChABC-GFP plasmids (A) or injected with ChABC (mixed in Pluronic-F172 hydrogel) (B,E), and immunostained for CSPG and Sox2 (A,B) or Tuj1 (E). Electroporated areas (green) are shown in the merged (red and green) images. (C,D) Quantification of CSPG and Sox2 staining in electroporated hindbrains (C) (*n*=10 control; *n*=14 ChABC for Sox2 and CSPG) or in injected hindbrains (D) (*n*=13 control; *n*=17 ChABC for CSPG). (F) Transverse sections of HB regions electroporated with control-GFP or ChABC-GFP plasmids, immunostained for Tuj1. Areas outlined are presented at higher magnification at the side or bottom of the image, showing phenotypes of typical apical-basal polarity (control) or mantle-positioned axons (ChABC). Arrow indicates an apically abscised and radially migrating cell. (G) Quantification of Tuj1 staining in injected hindbrains (*n*=20 control; *n*=20 ChABC). (H) Quantification of control and ChABC electroporated GFP^+^ cells of different migratory phenotypes (*n*=5 control, *n*=8 ChABC). Data are mean±s.d. (two-tailed unpaired *t*-test). *P<0.05, **P<0.005, ***P<0.0005. Scale bars: 200 µm in A; 500 µm in B,E; 100 µm in F.

To test the role of CSPG in the hindbrain, we knocked down CSPG by using the procaryote enzyme chondroitinase ABC (ChABC), which digests the CS-chains on CSPG (Muir et al., 2010). Soluble ChABC was either injected into the hindbrain lumen (mixed into a thermo-sensitive hydrogel) or electroporated as ChABC-GFP-encoding plasmid into one side of the neuroepithelium. The significant decrease in CSPG levels was confirmed for both treatments by comparing the immunostaining pattern and intensity in control and treated embryos (Fig. 2A-D), which was also validated by flow-cytometry analysis (Fig. S4B). Notably, ChABC-treatment was not coupled with increased cell death (Fig. S4A) or with disorganization of the hindbrain gross-segmentation (Fig. S4C).

**Fig. 3.**
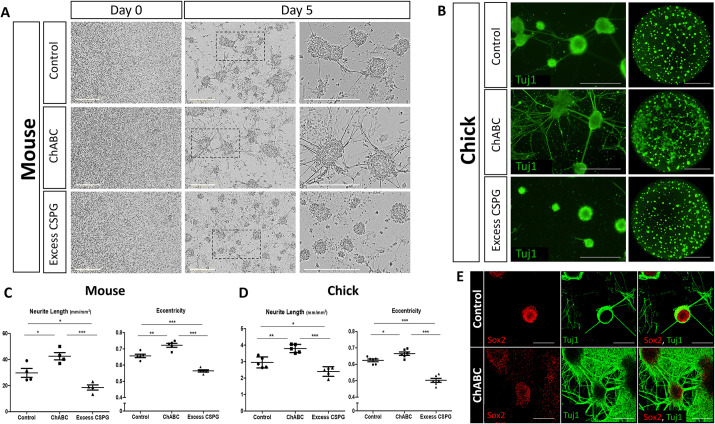
**Changes in CSPG levels modify hindbrain cell behavior *in vitro*.** (A) Phase-contrast images from time-lapse analysis of primary cultures of E10.5 mouse hindbrains treated with BSA (control), ChABC or excess CSPG. The areas outlined in the middle column are shown at higher magnification in the right column. (B) Primary cultures from chick hindbrains, on day 5 of incubation, treated as in A and immunostained for Tuj1. Whole-well images of Tuj1-stained cells are presented on the right. (C,D) Quantification of eccentricity and neurite-length in mouse or chick cultures. Each dot represents an average of six wells from four experimental replicates (mouse), or six to eight wells from five experimental replicates (chick). (E) Images of control and ChABC-treated primary cultures of HH18 chick hindbrains immunostained for Tuj1 and/or Sox2. Data are mean±s.d. (one-way ANOVA with a post-hoc Tukey's test). *P<0.05, **P<0.005, ***P<0.0005. Scale bars: 400 µm in A,B; 2000 µm in whole-well image in B; 100 µm in E.

Analysis of Sox2 expression in the ChABC-treated hindbrains revealed a significant reduction in Sox2 levels compared with controls (Fig. 3A-D). As a reduction in Sox2 expression may shift the cells towards a more differentiated state, we also examined the expression of the early neural differentiation marker β-tubulin III (Tubb3, also known as Tuj1). Typically, Tuj1 expression is strongly detected in the soma and axons of neurons migrating to the mantle layer of HBs, whereas Sox2^+^ cells are situated more apically in the ventricular and/or subventricular layers of the HB (Fig. S5). Evidently, in both chick and mouse hindbrain, Tuj1 is also occasionally expressed together with Sox2 in some cells that detach from the apical layer and migrate basally (Fig. S5; Das and Storey, 2014; Memberg and Hall, 1995; Peretz et al., 2016). The addition of ChABC resulted in an increased expression of Tuj1 (Fig. 2E,G), as also confirmed by qRT-PCR and flow-cytometry analyses (Fig. S6A,B), indicating that administration of ChABC in the hindbrain has a notable impact on neural differentiation. Interestingly, upregulation of Tuj1 was observed not only at the HBs, where CSPG is predominantly expressed, but also in the Rhs. This result may indicate that CSPG has a global, non-cell autonomous, effect outside its sites of expression. It is also plausible that a shift in the state of the few Sox2^+^ and/or CSPG^+^ cells located in the rhombomeres (Fig. 1, Fig. S2) also contributed to this widespread increase in neural differentiation.

To trace the effect of CSPG loss on individual HB cells, hindbrains were electroporated with control-GFP or ChABC-GFP plasmids and transversely sectioned 18 h later to visualize the behavior of GFP^+^ cells along the apical-basal axis of the HBs (Fig. 2F). Sections were also stained for Sox2 and Tuj1 to assess the differentiation state of the GFP^+^ cells, while also enabling the specific analysis of HB regions, based on the broader Sox2 expression at these domains (Fig. S6C). Most of control-GFP cells exhibited an organized apical-basal polarity, with a few cells that began to detach from the apical surface and migrate towards the mantle zone (Fig. 2F, Fig. S6D). ChABC-GFP cells were much more frequently observed to lose their apical-basal polarity, migrate basally and extend Tuj1^+^ axonal filaments in the mantle zone, while downregulating Sox2 expression in their soma (Fig. 2F, Fig. S6D). Quantification of the cell behavior patterns was carried out by scoring the abundance of individual GFP^+^ HB cells in three cell states: (1) radial glia morphology and/or apical-basal polarized cells; (2) apically abscised and/or radially migrating cells; and (3) mantle-positioned cells and/or axons. A higher percentage of ChABC-expressing cells have been found in states 2 and 3 compared with control cells (Fig. 2H). These results indicate that the absence of CSPG in individual HB cells promotes an accelerated progression toward neuronal differentiation.

The role of CSPG was next analyzed *ex vivo*. We have previously demonstrated the ability of hindbrain cells to grow in primary cultures using media that support the survival of NPSCs, rather than their differentiation. Most of the cells behaved as typical cultured NPSCs, as they formed floating neurospheres, whereas others gradually adhered to the surface, created monolayers and extended neurites, as expected from differentiating neurons *in vitro* (Peretz et al., 2018). Marker characterization of these neurospheres has demonstrated the distribution of Sox2^+^ cells detected throughout the spheres, whereas the early differentiation marker Tuj1, and the late differentiation markers Map2 and 3A10, were mostly evident in the periphery of the sphere. However, some differentiating cells still expressed Sox2, indicating that Sox2 downregulation during neural differentiation *in vitro* is gradual. Moreover, when spheres collapsed, they begun to form monolayers with networks of Tuj1-, Map2- and 3A10-expressing neurites (Peretz et al., 2016, 2018).

Based on this knowledge, similar primary cultures were prepared from HH18 chick or E10.5 mouse hindbrains and grown in NPSC-promoting media, with added ChABC or external CSPG compound, to simulate the effect of loss or excess CSPG (Sirko et al., 2010a). Flow-cytometry analysis revealed a ∼50% reduction in CSPG^+^ cells upon ChABC treatment (Fig. S7B), as also detected by immunofluorescence staining (Fig. S7A), confirming its inhibitory activity *in vitro*. Cells were monitored by live imaging for 5 days, after which distinct growth patterns were observed: control cells displayed multiple phenotypes, mostly including free-floating spheres, with some adherent spheres and extending neurites; ChABC-added cells did not demonstrate any free-floating spheres but instead developed into large adherent spheres that flattened into monolayers and extended many neurites; and cells exposed to excess CSPG remained mostly rounded as free-floating spheres (Fig. 3A and Movies 1-3 for mouse, Fig. S8A and Movies 4-6 for chick). Quantification of neurite length and sphere eccentricity illustrated the contrasting effect of modifications in CSPG levels (Fig. 3C,D). Eccentricity can range from 0-1, with 0 represent a full-rounded sphere, while neurite extension from the growing spheres and the formation of monolayers consequently increase the eccentricity value. As expected, eccentricity was highest in the ChABC-treated cells and lowest in the excess CSPG group. Simultaneously, neurite length increased upon CSPG loss, simulating the enhanced formation of neurites, which indicates a more differentiated cell state. Immunostaining for Tuj1 further highlighted the extensive neural differentiation and neurite formation upon CSPG loss, compared with both control and excess CSPG-treated cells, as the latter displayed very few Tuj1^+^ neurites (Fig. 3B for chick, Fig. S8B for mouse). Co-staining the cells for Sox2 and Tuj1 also demonstrated this phenotype, as Sox2 levels decreased along with expanded staining of Tuj1 in neurites of the CSPG-depleted cultures (Fig. 3E). Similarly, evaluating early [doublecortin (DCX)] and late (Map2) neural differentiation markers further emphasized the distinctions in the characteristics of the spheres within the different cultures, highlighting the emergence of neurites from the spheres after ChABC treatment, wherein both markers are expressed (Fig. S9). Altogether, the corresponding patterns observed in the chick and/or mouse hindbrain cultures signifies a conserved role for CSPG in preventing neural differentiation.

**Fig. 4.**
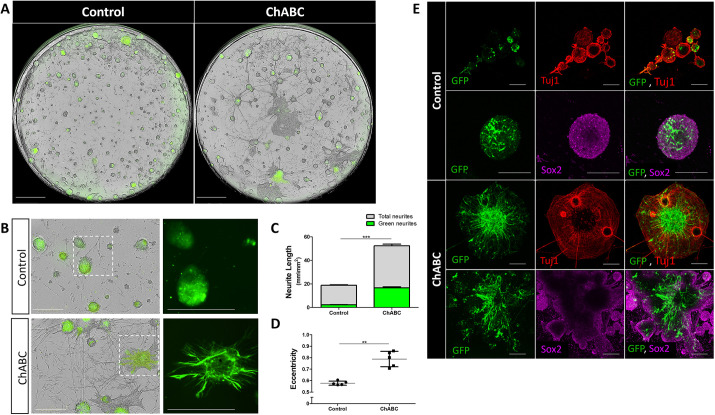
**Effect of ChABC on individual cells *in vitro*.** (A,B) Whole-well views (A) and single images (B) on day 5 of incubation of primary cultures, obtained from chick hindbrains electroporated with control-GFP or ChABC-GFP plasmids (phase contrast with GFP). Areas outlined in B are presented at higher magnification on the right. (C,D) Quantification of neurite length in GFP^+^ cells (green neurites) or in all cells (total neurites) (C), or sphere eccentricity (D). Bars (C) or each dot (D) represent an average of four to six wells from five experimental replicates. (E) Images of primary cultures of control or ChABC electroporated hindbrains, immunostained for Tuj1 or Sox2. Data are mean±s.d. (two-tailed unpaired *t*-test). **P<0.005, ***P<0.0005. Scale bars: 1000 µm in A; 400 µm in B; 1200 µm in E.

To trace how CSPG-loss affects individual cells *in vitro*, chick hindbrains were electroporated with a control- or ChABC-GFP plasmids, and similarly used for primary cultures. Whole-well views of the cultures clearly demonstrated once again an extensive neural differentiation pattern in the ChABC-expressing wells (Fig. 4A). Time-lapse analysis of the electroporated cells showed that most control-GFP cells remained rounded as an integral part of the sphere, whereas ChABC-GFP expressing cells displayed a marked phenotype of extension of neurites reaching out from the spheres (Fig. 4B, Fig. S10, Movies 7 and 8). Comparing neurite length between the cultures clearly showed the enhanced neurite formation in the ChABC-treated group, as also demonstrated by Sox2 and/or Tuj1 staining (Fig. 4C-E). Being predominantly present within rounded spheres, control-GFP cells were primarily Sox2^+^, whereas ChABC-GFP entities were notably depleted of Sox2^+^ cells, concurrently forming intricate Tuj1^+^ fibers around them (Fig. 4E). Interestingly, the increased formation of neurites in the ChABC culture was also evident in non-electroporated cells (Fig. 4A,B, total neurite length in Fig. 4C). This could imply that CSPG has a global and/or non-cell autonomous role in the hindbrain, in agreement with the ectopic Tuj1 expression found when ChABC was added *in vivo* (Fig. 2E). Yet, it is also possible that the ChABC-mediated effect in non-electroporated nearby cells is gained due to the fact that it is a secreted protein. Altogether, the *in vitro* results recapitulated the *in vivo* data, collectively suggesting a conserved role for CSPG in promoting a non-differentiated cell state in the hindbrain.

### Transcriptomic profiling of CSPG-based separated hindbrain cells

**Fig. 5.**
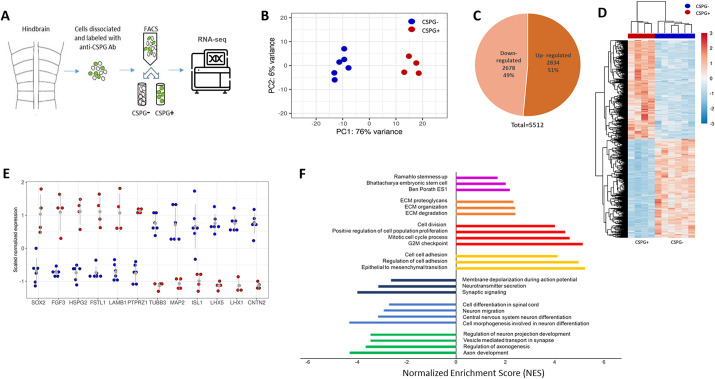
**Differential expression patterns and functions distinguish CSPG^+^ from CSPG^−^ cell groups.** (A) Scheme of the experimental procedure. (B) PCA based on the overall expression pattern of the six CSPG^−^ (blue) and four CSPG^+^ (red) samples. The first two components and their percentage of the total variance are shown. (C) Distribution of up- and downregulated genes, as indicated. All genes with a *P*adj<0.05 were included. (D) A heatmap representation of scaled normalized expression signals. Scaled values are colored according to the scale on the right (blue, negative values; red, positive values). Both genes (rows) and samples (columns) were hierarchically clustered, as shown by the dendrograms to the left and above the heatmap, respectively. Group identity of samples, CSPG^−^ or CSPG^+^, is indicated as text below the heatmap, as well as by color annotation above it. (E) Scaled normalized signal of specific genes indicated below the graph is shown for each sample, colored by group identity (blue, CSPG^−^; red, CSPG^+^). The average signal for each group is shown as a grey dot, with lines indicating the s.d. (F) Gene set enrichment analysis: up and downregulated gene sets. The change degree is measured by the normalized enrichment score (NES). Purple, orange, red and yellow: upregulated gene sets (higher expression in CSPG^+^ versus CSPG^−^). Blue, light blue and green: downregulated gene sets (lower expression in CSPG^+^ versus CSPG^−^).

As membrane-bound CSPG is highly expressed in the HBs, particularly surrounding Sox2^+^ cells, we employed its membrane-bound expression (Fig. 1, Fig. S1) to isolate the HB^−^Sox2^+^ cells, aiming to perform a comparative bulk RNA-seq analysis to fully elucidate the transcriptome of the HB. Approximately 30 hindbrains of HH18 chick embryos were pooled, dissociated into single cells, immunostained for CSPG and processed with FACS (Fig. 5A). This sorting yielded two cell fractions: CSPG-expressing cells (CSPG^+^), which were expected to be enriched with HB cells and made up 18.4% of all cells; and CSPG-negative cells (CSPG**^−^**), which made up 53.5% of all cells and consisted mostly of Rh cells, as well as the mantle layer of the HBs (Sox2^−^). Cells that displayed low levels of CSPG expression were excluded from the analysis (Fig. S11). Notably, DAPI^+^ dead cells were also omitted from the gating, resulting in 95.8% of total hindbrain cells that were used for each FACS series. This procedure was repeated six times. All biological replicates were forwarded for RNA-seq.

Principal component analysis (PCA) of the expression signal showed that the CSPG^+^ and CSPG^−^ replicates were clustered into two highly distinct groups (Fig. 5B). Differential gene expression (DEG) analysis revealed 5512 differentially expressed genes (adjusted *P*<0.05), out of which 2834 were significantly upregulated and 2678 were significantly downregulated in CSPG^+^ cells compared with the CSPG^−^ group (Fig. 5C), providing a rich data source for further analyses. The expression signal of these differentially expressed genes was reproducible between our biological replicates, as can be seen by the unsupervised hierarchical clustering of samples (Fig. 5D), further indicating the robustness of our experimental design. RNA-seq results for specific genes selected based on previous knowledge of their expression patterns are presented in Fig. 5E. For example, *SOX2*, *FGF3*, heparan sulphate proteoglycan (*HSPG2*)*,* follistain (*FSTL1*), laminin B1 (*LAMB1*) and the membrane-bound type of CSPG (*PTPRZ1*), all of which have previously been reported to be expressed in chick HB cells (Heyman et al., 1995; Peretz et al., 2016, 2018; Weisinger et al., 2012), were significantly upregulated in the CSPG^+^ group. In contrast, various neural differentiation markers, previously reported to be expressed in hindbrain post-mitotic neurons or in axonal fibers in the mantle zone, such as class III β-tubulin (*TUBB3*), *MAP2*, *ISL1*, *LHX1*, *LHX5* and *CNTN2* (also known as *TAG1*), (Kohl et al., 2012, 2015; Peretz et al., 2016, 2018), were significantly upregulated in the CSPG^−^ cell fraction.

Gene-set enrichment analysis (GSEA) of the RNA-seq data revealed significant variances in the expression of genes from multiple functional categories between the groups (Fig. 5F). Gene sets related to embryonic stem cells and cell division were upregulated in the CSPG^+^ cells (Fig. 5F, purple and red bars), along with gene sets related to cell adhesion and ECM organization (Fig. 5F, yellow and orange bars). Conversely, multiple gene sets linked to neural differentiation, axonal projection and neuronal activity were upregulated in the CSPG^−^ cell group (Fig. 5F, green, light-blue and dark-blue bars). Overall, it appears that the CSPG^+^-HB cells display typical characteristics of amplifying NPSCs with a well-defined ECM, whereas the CSPG^−^ cells embody characteristics of differentiating and mature neuronal cells. Moreover, the GSEA results are consistent with our previous findings, where localization of Sox2 and other progenitor markers was confined to the ECM-rich HBs, while neuronal differentiation occurred in the Rhs and/or mantle zone of the HBs (Peretz et al., 2016).

**Fig. 6.**
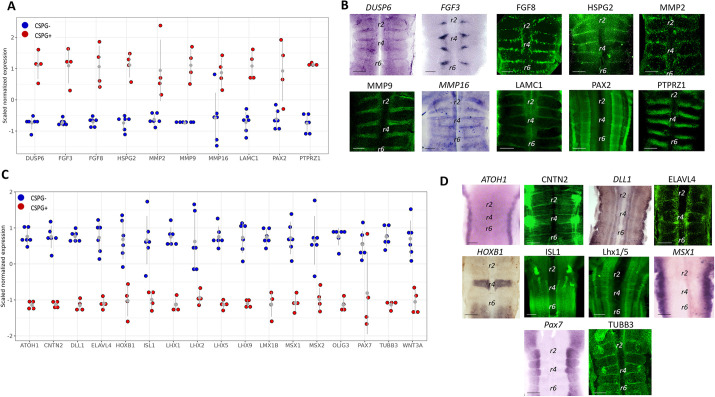
**Validation of DEGs by *in situ* hybridization or immunofluorescence staining.** (A,C) Scaled normalized signal of specific genes (indicated below the graph) is shown for each sample, colored by group identity (blue, CSPG^−^ group; red, CSPG^+^ group). Grey dots represent average signal, lines indicating s.d. All genes in A are upregulated in the CSPG^+^ samples; all genes in C are upregulated in the CSPG^−^ samples. (B,D) Flat-mount views of hindbrains stained for the different genes by *in situ* hybridization (purple) or immunofluorescence (green). In all panels, flat-mounted hindbrains are ventricular side up, except for *CTNT2*, *ELAVL4* and *TUBB3*, where hindbrains are mantle side up. Scale bars: 200 µm. r, rhombomere.

Experimental validation of DEGs was conducted through *in situ* hybridization or immunostaining (Fig. 6). Consistent with genes significantly upregulated in the CSPG^+^ group [i.e. *DUSP6* (also known as *MKP3*), *FGF3*, *FGF8*, *HSPG2*, matrix metalloprotease *2* (*MMP2*), *MMP9*, *MMP16*, laminin subunit gamma 1 (*LAMC1*), *PAX2* and *PTPRZ1*], their expression was distinctly pronounced at the HBs or enhanced at these sites, even when not fully confined (*PAX2*, for example) (Fig. 6A,B). Conversely, DEGs that were significantly upregulated in the CSPG^−^ group (Fig. 6C) were expressed in domains known to contain post-mitotic neurons or axonal filaments at the mantle layer [i.e. *CNTN2*, *ELAVL4* (also known as HuD), *ISL1*, *LHX1*, *LHX5* and *TUBB3*], in specific rhombomeres (*Hoxb1*) or in longitudinal columns that exclude the HBs (*DLL1* and *PAX7*) (Fig. 6D). Finally, genes known to be expressed in the dorsal hindbrain, such as *ATOH1*, *MSX1*, *MSX2*, *OLIG3*, *LHX2, LHX9* and *WNT3A* (Hirsch et al., 2021; Hollyday et al., 1995; Landsberg et al., 2005; Mishima et al., 2009; Wang et al., 2005), were found to be significancy upregulated in the CSPG^−^ group (Fig. 6C), as also validated by the expression patterns of *ATOH1* and *MSX1* (Fig. 6D), indicating that the CSPG^+^ cell group does not contain dorsal-most cells. Altogether, these results confirm the separation of the hindbrain cells into the expected cell groups, each expressing a different set of genes in the HBs or in non-HB regions.

**Fig. 7.**
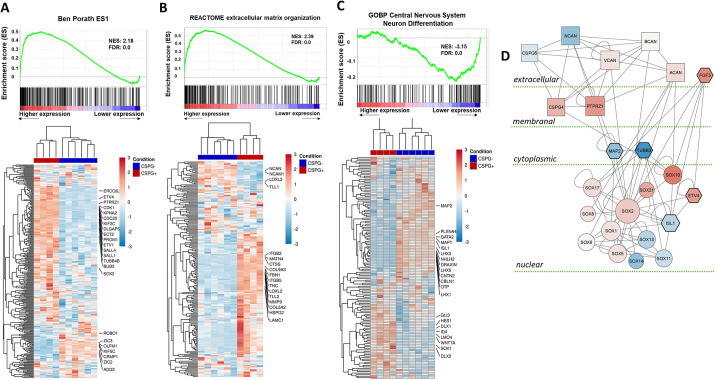
**Gene set enrichment analysis of selected pathways.** (A-C, upper part) GSEA-generated graphics showing the distribution of genes, represented by vertical lines on either the left side (A and B, upregulated in CSPG^+^ versus CSPG^−^) or the right side (C, downregulated in CSPG^+^ versus CSPG^−^). (A-C, lower part) Heatmap of genes belonging to the gene set, showing the same trends (A and B, upregulation; C, downregulation). (D) Protein-protein network of functionally important genes in CSPG^+^ versus CSPG^−^. Each shape represents a protein, lines connect interacting proteins. Blue, downregulated genes; red, upregulated genes; round, Sox genes; square, CSPGs; hexagon, experimentally validated genes.

To further investigate some of the most enriched gene sets of the GSEA analysis (Subramanian et al., 2005), expression of the full sets of genes were presented as hierarchically clustered heatmaps. This showed that a high proportion of the genes in these sets are clustered with a distinct expression pattern of either up or down-regulation. Using the embryonic stem cell pathway ‘Ben Porath ES1’, multiple genes related to maintenance and self-renewal of stem cells, which were upregulated in the CSPG^+^ group, clustered together in a dense area of the heatmap (Fig. 7A). For example, the transmembrane glycoprotein *PROM1*, the zinc-finger transcription factors (TFs) *SALL1* and *SALL4*, the ETs-related TFs *ETV1* and *ETV5*, and the cell cycle regulatory genes *CDK1* and *CDC20* (Akagi et al., 2015; Exner et al., 2017; Walker et al., 2013; Wei et al., 2021; Yamano, 2019) all displayed remarkable upregulation. Genes related to cancer stem cells and tumorigenesis, such as *KPNA2*, *ECT2*, *ERCC6L* and *TUBB4B*, and the cell-cycle regulators *DLGAP5*, *KIF2C* and *BUB3* (Christiansen and Dyrskjøt, 2013; Dharmapal et al., 2021; Gong et al., 2020; Pu et al., 2017; Silva and Bousbaa, 2022; Sun et al., 2017; Tsou et al., 2003) were also significantly upregulated in the CSPG^+^ group, and clustered well within the same area. Notably, *SOX2* and *PTPRZ1* were also found in this gene set, indicating their relevance to HBs. Concurrently, even though not contributing to the gene set enrichment score, evaluation of the most notable downregulated genes in this gene set further confirmed the NPSC-like properties of the CSPG^+^ cells. Genes involved in neural differentiation and axonogenesis (i.e. *ROBO1*, *OLFM1*, *CRMP1*, *KIF5C*, *ADD2* and the TF genes *ZIC2* and *ZIC3*; Aruga, 2004; Guthrie, 2004; Kanai et al., 2000; Matsuoka et al., 1998; Nakaya et al., 2008; Yamashita and Goshima, 2012) were notably downregulated in the CSPG^+^ group (Fig. 7A). In addition, some of these genes also regulate neural induction at earlier stages, indicating their context-dependent roles during development.

Furthermore, analysis of the ‘Reactome ECM organization’ gene set revealed many ECM-related genes that were markedly upregulated in the CSPG^+^ cell fraction (Fig. 7B). These included the cell surface integrin proteins *ITGB2* and *ITGB5* (Gardiner, 2011; Ikeshima-Kataoka et al., 2022), various ECM proteins, such as the fibrillar proteins fibrillin (*FBN1*) and collagen subtypes *COL9A3* and *COL5A2* (Ricard-Blum, 2011), the ECM-adaptor protein matrilin (*MLN4*) (Uckelmann et al., 2016), the tenascin glycoprotein family member *TNC* (Midwood et al., 2016), the sulphated proteoglycan *HSPG2* (Sarrazin et al., 2011), the laminin glycoprotein member *LAMC1* (Aumailley, 2013), and ECM-remodeling protease genes, such as *MMP9* (Monsonego-Ornan et al., 2012) and cathepsin C (*CTCC*) (Tran and Silver, 2021). Conversely, ECM and cell-adhesion proteins that participate in axonal growth and neural differentiation, such as *NCAM* (Paratcha et al., 2003) and the CSPG-soluble protein *NCAN* (Zhou et al., 2001), were markedly downregulated in the CSPG^+^ group, further illuminating the non-differentiated state of this group.

Synchronously, many genes related to neural specification, differentiation, migration and axonogenesis have been significantly upregulated in the CSPG^−^ group, contributing to the enrichment of the ‘GOBP Central Nervous System Differentiation’ gene set (Fig. 7C). These included several types of TFs, such as the ventral neuronal markers *GATA2* and *ISL1* (Liang et al., 2011; Zhou et al., 2000), the dorsal interneuron markers *LHX1*, *LHX3* and *LHX5* (Hirsch et al., 2021; Pillai et al., 2007), the dopaminergic neuronal marker *OTP* (Ryu et al., 2007), and the regulator of precerebellar nuclei migration *NHLH2* (Schmid et al., 2007). Moreover, genes encoding various axonal-growth cues and receptors, such as the semaphorin receptor *PLXNA4* (Suto et al., 2005), the repulsive signal *DRAXIN* (Ahmed et al., 2011), the chemoattractant signal *CBLN1* (Han et al., 2022), the microtubule-associated proteins *MAP2* and *MAPT* (Riederer and Matus, 1985), and the cell-adhesion molecule *CNTN*, which promotes axon guidance and fasciculation (Stoeckli and Landmesser, 1995), were all significantly upregulated in the CSPG^−^ cell group (Fig. 7C). Accordingly, gene encoding factors that promote cell proliferation and prevent neural differentiation in NPSCs and/or neuroepithelial cells were clearly downregulated in the CSPG^−^ group. These included the TFs *HES1* (Kageyama et al., 2008), *DLX1* and *DLX2* (Carrillo-García et al., 2010), *ID4* (Bedford et al., 2005), *LMO4* (Kashani et al., 2006), *SOX1* (Venere et al., 2012) and the cell-cycle-regulated protein *HURP* (hepatoma up-regulated protein) (Tsou et al., 2003).

Finally, protein-protein interaction network analysis revealed the putative crosstalks between different CSPG subtypes, FGF signaling components, neural differentiation markers and various Sox genes that are up or downregulated in the CSPG^+^ cell group, in accordance with their extra- or subcellular localization (Fig. 7D). This analysis further demonstrates the direct interaction of Sox2 with the membranal CSPG subtype PTPRZ1 and the soluble signal molecule FGF3, in agreement with their spatial expression patterns (Fig. 1, Fig. S1) (Weisinger et al., 2012). Collectively, the divergence of the whole-transcriptomic data, together with the enrichment of the gene-set categories and networks, substantiate that HB cells are a subpopulation of NPSCs that aggregate in specific niches at the HBs, differing from the adjacent more differentiated CSPG^−^ and/or Rhs cells.

### CSPG^+^ HB cells reveal typical NPSC-like behavior *in vivo*

**Fig. 8.**
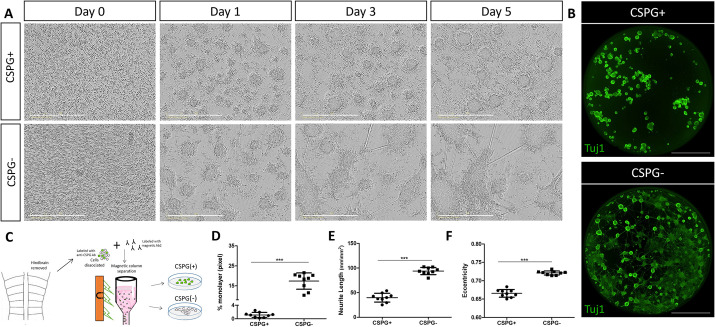
**CSPG-based separated cells show distinct characteristics *in vitro*.** (A) Phase-contrast images from time-lapse analysis of chick hindbrain cultures, separated into CSPG^+^ and CSPG^−^ groups. (B) Whole views of Tuj1-stained CSPG^+^ and CSPG^−^ cells. (C) Illustration of the magnetic-based immuno-column procedure. (D-F) Quantification of monolayer, neurite length and sphere eccentricity in CSPG^+^ and CSPG^−^ cells. Each dot represents an average calculated in one well at day 5 of incubation. *n*=9 wells for each group from three experimental replicates. Data are mean±s.d. (two-tailed unpaired *t*-test). ***P<0.0005. Scale bars: 400 µm in A; 2000 µm in B.

As the transcriptome of HB-Sox2 cells appeared to resemble that of other types of NPSCs, we next examined whether these molecular properties are coupled with the typical behavior of NPSCs. To separate the HB cells from the rest of hindbrain cell populations, we once again relied on the membrane-bound CSPG expression in HB-Sox2 cells (Fig. S1). Hindbrains of chick embryos were dissociated and immunolabeled for CSPG, then passed through a magnetic-based immunocolumn, ultimately providing two viable CSPG^+^ and CSPG^−^ cell fractions (Miltenyi et al., 1990) (Fig. 8C). This sorting method was selected as the cells demonstrated a higher viability during prolonged incubation, in comparison with FACS-sorted cells. Flow-cytometry analysis confirmed an efficient separation, which demonstrated the enrichment of cells expressing CSPG in the CSPG^+^ fraction compared with the CSPG^−^ fraction (Fig. S12). The CSPG^+^ and CSPG^−^ cell groups were seeded and live imaged for 5 days, followed by immunostaining for Tuj1 at the end of the incubation. Noticeably, the two cell fractions displayed distinctly dissimilar characteristics: CSPG^+^ cells mostly gathered into rounded spheres with very few cells that adhered and extended neurites, whereas the CSPG^−^ cells largely adhered and showed high extension of neurites (Fig. 8A; Movie 9 for CSPG^+^; Movie 10 for CSPG^−^). This extensive neurite formation was further demonstrated by a whole-well view of Tuj1-immunostained cells, as the CSPG^−^ cells revealed a substantial formation of entwined network of Tuj1-expressing neurites, extending in and between the adhered spheres, while the CSPG^+^ cells were found in rounded spheres, almost completely devoid of Tuj1+ neurites (Fig. 8B). As monolayer structure is typical for differentiating neurons, formation of monolayer was quantified in the two cell fractions and was found to be higher in the CSPG^−^ group (Fig. 8D). Neurite length and eccentricity were also significantly higher in those cells, further revealing the more differentiated nature of the CSPG^−^ cells compared with the CSPG^+^ population (Fig. 8E,F). Together, the noticeably distinguished behavior of the two cell populations further verifies that the CSPG^+^ fraction is enriched with cells displaying typical NPSC phenotypes, whereas the CSPG^−^ fraction comprises more-differentiated hindbrain cells.

### CSPG is required to maintain HB-Sox2 cells in their NPSC-like state

**Fig. 9.**
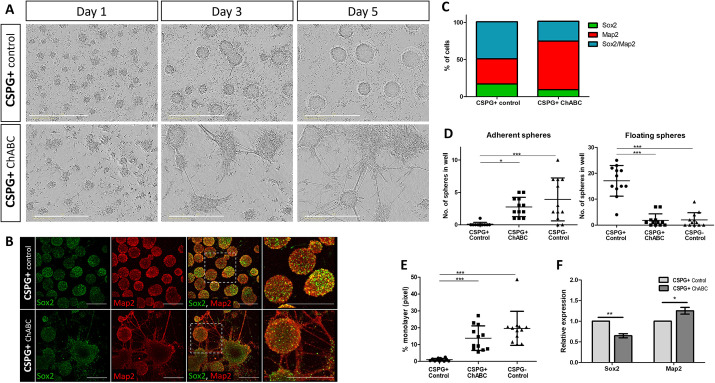
**CSPG loss promotes boundary cell differentiation.** (A,B) Phase-contrast (A) or fluorescent (B) images of time-lapse analysis of control-CSPG^+^ cells or ChABC-treated CSPG^+^ cells. Images in B are immunostained for Sox2 and Map2. Areas outlined in B are shown at higher magnification on the right. (C) Quantification of cells expressing Sox2 or Map2, or both, in control and ChABC-treated CSPG^+^ cells. *n*=9 wells for each group from three experimental replicates. (D,E) Quantification of monolayers and floating or adherent spheres in control CSPG^+^ and CSPG^−^ cells, and in ChABC-treated CSPG^+^ cells. Each dot represents an average calculated in one well at day 5 of incubation. *n*=12 wells for each group from three experimental replicates. Data are mean±s.d. (one-way ANOVA with a post-hoc Tukey's test). (F) qRT PCR analysis of *Sox2* and *Map2* expression in control and ChABC-treated CSPG^+^ cells. Data are mean±s.d. from three experimental replicates: *n*=4 wells for each group (two-tailed unpaired *t*-test). *P<0.05, **P<0.005, ***P<0.0005. Scale bars: 400 µm in A; 100 µm in B.

Finally, to fully uncover the effect of CSPG on HB-Sox2 cells, we sought to directly examine the behavior of the HB CSPG^+^ cells following CSPG removal. Live-imaging analysis showed the untreated CSPG^+^ cells typically aggregating to form rounded spheres (Fig. 9A, Movie 11). However, addition of ChABC to the CSPG^+^ cells caused them to rapidly adhere and extend many neurites (Fig. 9A, Movie 12), thus exhibiting a remarkable resemblance to the CSPG^−^ cell fraction (Fig. 8A). To quantify the shift in the state of the cell, formation of monolayers and development to adhered and/or floating type of spheres were measured in the control and ChABC-treated CSPG^+^ cells (Fig. 9D,E). Appropriately, control cells formed almost exclusively floating spheres, while formation of monolayers was highest in the ChABC-treated group. This behavior was comparable with that detected in the CSPG^−^ cell group, which also generated mostly adherent spheres, indicating that loss of CSPG in HB-Sox2 cells is sufficient to shift them into a more differentiated state, as evident in CSPG^−^ cells. Immunostaining for Sox2 and/or Map2 further elucidated the change in the differentiation state, as Sox2 expression was found to be more profound in control cells compared with a weaker expression in the ChABC-treated cells (Fig. 9B). Moreover, Map2, which was expressed in the outer layer of the spheres in the control and ChABC-treated cells, was also found in the extending neurites that were formed almost exclusively in the latter group (Fig. 9B). Analysis of the ratio of Sox2- to Map2-positive cells also revealed this distinct shift in cell state. Control cells predominantly exhibited positivity for both markers, indicating that Map2^+^ cells at the periphery of the spheres retained their progenitor identity. In contrast, CSPG-treated cells, having progressed beyond the progenitor state, displayed a higher prevalence of differentiation marker expression, particularly in the extensively formed neurites (Fig. 9C). qRT-PCR analysis of relative gene expression in the two cell groups also showed a decrease in *Sox2* and an increase in *Map2* levels in CSPG^+^ cells upon treatment with ChABC (Fig. 9F).

**Fig. 10.**
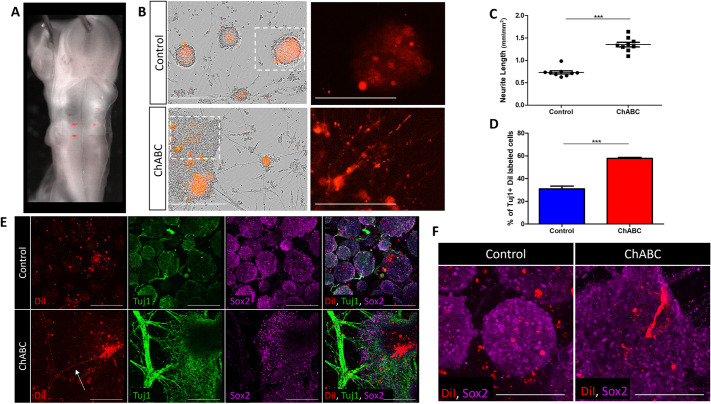
**Effect of CSPG loss on CM-DiI-labeled boundary cells.** (A) View of CM-DiI-labelled HBs in HH15 chick embryo. (B) Images of control and ChABC-treated cultures, prepared from CM-DiI-labeled hindbrains, showing unlabeled or CM-DiI-labelled cells (phase contrast or red, respectively). Areas outlined are shown at higher magnification on the right, showing labeled cells only. (C) Quantification of neurite length in CM-DiI-labelled cells. Each dot represents an average calculated in one well at day 5 of incubation. *n*=9 wells for each group from three experimental replicates. (D) Quantification of CM-DiI-labeled cells expressing Tuj1. (E) Images of CM-DiI-labeled cultures immunostained for Tuj1 and Sox2. Arrow indicates an extending neurite originating from a labeled HB cell. (F) Representative spheres stained for Sox2 from control and ChABC-treated CM-DiI-labeled cultures. Data are mean±s.d. (two-tailed unpaired *t*-test). ***P<0.0005. Scale bars: 400 µm in B (200 µm for higher magnification images); 200 µm in E; 100 µm in F.

Finally, we aimed to trace the specific behavior of single HB cells after CSPG loss, when cultivated in the presence of all other hindbrain cells. HB cells were manually labeled with the lipophilic dye CM-DiI (Fig. 10A), after which cultures were prepared and treated with ChABC. Evidently, over time, control DiI-labeled cells remained integrated within rounded spheres, while DiI-labeled cells treated with ChABC flattened and extended neurites (Fig. 10B,E,F, Fig. S13, Movies 13 and 14). Quantification of neurite length in DiI-labeled cells confirmed the observed shift towards neural-differentiation behavior of HB cells in the absence of CSPG (Fig. 10C). Staining for Tuj1 further validated this phenotype, as the formed neurites were Tuj1^+^ (Fig. 10E), accompanied by a total increase in the percentage of Tuj1^+^ DiI-labeled cells (Fig. 10D). Conversely, while embedded within the spheres, control-DiI cells appropriately displayed Sox2 expression, whereas some ChABC-treated cells undergoing neural differentiation were observed to have lost this progenitor marker (Fig. 10E,F). Together, these analyses directly confirm the shift towards differentiation of HB cells in the absence of CSPG.

## DISCUSSION

This study reveals that the ventricular and/or subventricular layers of the HBs are enriched with cells expressing Sox2 and CSPG in mouse and chick embryos, and unveils the function of CSPG in maintaining these cells as NPSCs, as they reside in designated niches in between the Rhs. The transcriptomic profile of HB cells uncovered a substantial number of DEGs that participate in multiple types of embryonic, neural and cancer stem cell-related pathways, and in ECM-enriched pathways, which have not previously been identified in HB cells. Correspondingly, neural differentiation-related genes and pathways were found to be upregulated in the non-HB cells. Ultimately, this study highlights the unique position of HBs in amniotes as CSPG-enriched niches of NPSCs in between the differentiating Rhs, as well as contributing new data regarding genes and pathways that are active in these cells, which emphasizes the significance of compartment boundaries during development.

### HB cells are NPSCs

Highlighting the uniqueness of HBs as repetitive CSPG-rich niches of NPSCs in the hindbrain, raises the question of how similar are they to other NPSC types. Comparing our transcriptome data with knowledge on NPSCs from other domains reveals that, in addition to shared expression of Sox2 among many types of NPSCs, other NPSC landmark genes are also expressed in the HBs. This includes, for example, the membranal protein prominin 1 (*PROM1* or *CD133*) and the glutamate transporter family member *GLAST1* (*SLC1A3*). These markers are paramount for the self-renewal and acquisition of multi-lineage differentiation capacities by the NSCs from the SVZ and DG (Coskun et al., 2008; Lee et al., 2005; Uchida et al., 2000). Interestingly, PROM1 and GLAST1 are also upregulated in brain cancer cells and correlate with poor prognosis, demonstrating the intricate relationship between NSCs and brain tumors (Reya et al., 2001; Singh et al., 2004). These findings raise the question of whether abnormal development and/or maintenance of HB cells can participate in brainstem tumors.

The milieu surrounding NPSCs in various CNS domains is enriched with different ECM proteins that dictate their proliferation, differentiation and fate (Kazanis and ffrench-Constant, 2011; Preston and Sherman, 2011; Walma and Yamada, 2020). For example, the secreted glycoprotein tenascin C (TNC) and the ECM receptor integrin b (ITGB) modulate such processes in telencephalic or spinal cord NPSCs, as their loss of function decreased the number of NPSCs (Faissner et al., 2017; Garcion et al., 2001; Karus et al., 2011; Temple, 2001; Theocharidis et al., 2021). Our transcriptomic analysis found that *TNC*, *ITGB2* and *ITGB5*, were upregulated in HB cells, suggesting similar role in promoting the development of the HB NPSCs. Notably, in chick mesencephalic neural progenitors, integrin was found to activate Wnt7A signaling, which in turn induces the expression of the ECM molecule decorin (DCN) that promotes neurogenesis in neighboring cells (Long et al., 2016). Intriguingly, *WNT7A* and *DCN* were also upregulated in the HB and/or CSPG^+^ cell group, implying a conserved function for integrins in midbrain and hindbrain progenitors.

Several cell-cycle regulatory genes have been identified in various types of NPSCs and cancer-stem cells. For example, the kinesin molecule Kif2C and the anaphase-promoting factor CDC20 regulate proliferation of NSCs or tumorigenic brain cells (Li et al., 2022; Qi et al., 2020; Sun et al., 2017; Tu et al., 2022; Xie et al., 2015; Yang et al., 2019). These genes were upregulated in HB cells, along with the DNA excision repair gene *ERCC6L*, a pan-cancer marker that promotes growth and invasion in various cancers, including glioma (Chen et al., 2022; Xie et al., 2019). Mutations in this gene are linked to Cockayne syndrome, a rare inherited disorder characterized by neural abnormalities (Wang et al., 2020b). *ERCC6L*, which may be involved in regulating the proliferation of HB cells, has not yet been reported in other types of NSCs.

Altogether, these examples reveal several emerging genetic similarities between HB NPSCs and other NPSCs. The outcomes of their mis-regulation in other contexts could possibly unravel their role in the regulation of HB cells as NPSCs.

### The role of CSPS in the HBs

CSPG has been previously found to regulate the state of NPSCs in the brain. Yet, its removal from NPSCs using ChABC produces different outcomes, with some reporting reduced neurogenesis and proliferation, and others reporting increased proliferation, differentiation and migration (Gu et al., 2009; Sirko et al., 2007; Yamada et al., 2018). Our transcriptomic profiling revealed that *PTPRZ*, a gene encoding a transmembrane protein of the CSPG family, was significantly elevated in HB cells. This correlates with the membrane-bound pattern of DSD1 observed in our immunostaining, altogether suggesting that the HBs are enriched with PTPRZ. Previous studies using cortical and telencephalic NSCs have reported PTPRZ expression and found that its degradation using ChABC decreased their self-renewal and neurosphere formation, and increased their differentiation *in vitro* (Sirko et al., 2007, 2010b; von Holst et al., 2006). Our data further emphasize the significance of this CSPG in hindbrain NPSCs, as treatment with ChABC decreased Sox2 expression and promoted cell differentiation *in vivo* and *in vitro*. Notably, the effect of CSPG modifications on NSC fate differs in different CNS domains; interference with the sulfated state of the glycosaminoglycan (GAG) chains caused cortical-derived NSCs to differentiate towards the astrocytic lineage, whereas spinal cord NPSCs generated more-immature neurons *in vitro* (Karus et al., 2012; Sirko et al., 2007). Further investigation is required to determine the fate of NPSCs in the HBs, specifically when they are either enriched or depleted of CSPG.

How can CSPG maintain the HB cells in an undifferentiated state? As a highly abundant ECM factor, CSPG was found to interact with multiple signaling molecules, neurotrophic factors, ECM components and cell-adhesion proteins (Djerbal et al., 2017). Yet, most of these interactions occur in processes that follow NPSC stages, including neuronal migration, axonal pathfinding and synaptogenesis (Dyck and Karimi-Abdolrezaee, 2015; Mencio et al., 2021; Miller and Hsieh-Wilson, 2015; Rogers et al., 2011). Interestingly, CSPG is a key inhibitor of axonal growth upon CNS injury; hence, its elimination is essential for regeneration (Brown et al., 2012; Griffith et al., 2017). Modified CSPG levels are also related to mental and neurodegenerative disorders (Jang et al., 2020; Schultz et al., 2014), signifying the multi-faceted roles of CSPG in the CNS (Zhang and Chi, 2021). Contrary to its function in these processes, its role in NPSCs is less well known. Several studies have reported that CSPG is required for maintaining a stemness state in NPSCs in the SVZ, and have suggested that this activity is mediated by interaction with FGF2 to promote cell proliferation (Bian et al., 2011; Roll et al., 2022; Sirko et al., 2010a). In spinal cord NSPCs, such FGF2 activity was recently found to depend on the sulfation patterns of the CSPG-GAG chains that operate as docking sites for specific proteins (Schaberg et al., 2021). Intriguingly, our RNA-seq data did not find a differential expression of *FGF2* in the two CSPG-separated cell groups. However, other FGFs, including *FGF3*, *FGF8*, *FGF10*, *FGF18* and *FGF22*, were upregulated in the HB-cell fraction, suggesting that local CSPG may prevent cells from undergoing differentiation by acting as a co-receptor for these FGFs. This possibility is supported by the upregulation of several FGF-downstream target genes in the HB cells (such as *ETV4* and *ETV5*), as well as by our previous findings on the presence of di-phosphorylated ERK (dpERK) in these domains (Weisinger et al., 2012). Yet, it is possible that other signals, receptors or ECM factors interact with CSPG at the HBs (Tham et al., 2010). For example, transcripts of various ECM-related factors (collagens, integrins, laminin and fibronectin), which have been reported to interact with CSPG in other processes, such as metastasis, CNS injury and axonogenesis (Avram et al., 2014; Knutson et al., 1996; Ohtake and Li, 2015), were upregulated in the HB cell RNA-seq. Upon illuminating the significance of CSPG in regulating the state of HB cells, deciphering the pathways and the participating factors should be the next step.

We have previously reported that two subgroups of Sox2^+^ cells constitute the HBs: a main group composed of slow-dividing cells positioned in the core of the boundary; and a smaller group composed of faster-amplifying cells located at the boundary edges that either migrate and undergo differentiation at the HB mantle zone or enter the rhombomeres upon division (Peretz et al., 2016). The association between the HB cell state and the cell cycle was validated by blocking the cell cycle, which resulted in over-accumulation of Sox2^+^ cells at the HBs and their depletion from the rhombomeres. Similar features of NPSCs have also been reported in the hippocampal subventricular zone, where a quiescent subgroup serves as a reservoir of uncommitted NSCs that gives rise to a proliferating group of NPCs, which will later differentiate (Suh et al., 2007). This evidence raises the possibility that CSPG is involved in maintaining the HBs as slow-dividing progenitors and that its removal leads to an increased number of Sox2^+^ cells transitioning into a rapid-amplifying state – a necessary step before entering the rhombomeres and initiating the differentiation process. This scenario aligns with the observation of Tuj1 upregulation upon ChABC treatment, not only in HBs but also in the rhombomeres, suggesting that more HB cells become actively proliferating, resulting in an elevated population of Sox2^+^ cells migrating towards the rhombomeres and undergoing differentiation. As our preliminary evidence shows an increase in mitotically active Sox2^+^ cells in the HBs upon CSPG loss (C.H. and D.S.D., unpublished), exploring the CSPG-related mechanism that controls the cell cycle state of the NPSCs in HBs, awaits further research.

### HBs in different model systems

In contrast to the limited data on HBs in amniotes, extensive research has been performed in zebrafish. Zebrafish HB cells have been found to act as organizing centers to induce neurogenesis in rhombomere-flanking zones and to repel axons and drive their accumulation in the Rhs (Gonzalez-Quevedo et al., 2010; Terriente et al., 2012). In agreement with our previous finding on chick HBs as reservoirs of NPSCs (Peretz et al., 2016), subsequent work confirmed that zebrafish HBs similarly serve as pools of progenitors in an active proliferative state, regulated by Yap and Taz-TEAD activity (Voltes et al., 2019). Recent monitoring of the spatiotemporal dynamics of HB cells in zebrafish has revealed that they initiate as neuroepithelial stem cells that divide symmetrically, while they later shift to become radial glia progenitors undergoing asymmetrical division to contribute neurons to the hindbrain (Hevia et al., 2022). This transition was found to be triggered by Notch3 signaling. Intriguingly, although *NOTCH3* was not detected in our transcriptomic analysis, *NOTCH2* is upregulated in the chick HB cells, whereas two delta ligands (*DLL1* and *DLL4*) were upregulated in the non-HB fraction. These findings may indicate a conserved lateral inhibition mode of action of HB cells in avian and teleost to preserve HB cells in an undifferentiated state. This is further supported by the upregulation of the Notch downstream target *Hes1* in the transcriptome of the HB, as also previously found in mice HBs (Baek et al., 2006). Yet, the Notch signaling-supporting factors, radical fringe (*rfng*) and lunatic fringe (*lfng*), which are expressed in zebrafish HB cells and prevent them from undergoing differentiation (Nikolaou et al., 2009; Voltes et al., 2019), were not detected in our RNA-seq or in previous studies in mice (Moran et al., 2009). These differences may indicate inter-species variations in the of the gene profile of HBs, consistent with other factors that are expressed in avian and mammalian, but not fish, HBs, such as FGF3 and Sox2. These species-specific properties are also emphasized by the fact that, in zebrafish, Rh centers are another non-neurogenic zone that also regulate neural differentiation in their neighboring domains (Cheng et al., 2004; Gonzalez-Quevedo et al., 2010). This leads to the formation of repetitive neurogenic stripes in the Rhs that are found in between the HBs and Rh centers, but are not evident in amniotes.

Further comparison between HBs of zebrafish and chick can be drawn from a single-cell RNA-seq carried out in zebrafish hindbrain, which demonstrated three cell clusters (Tambalo et al., 2020): HB cells, Rh center cells and neurogenic cells. The HB cluster expressed genes such as *rasgef1ba*, *rac3b*, *prdm8*, *follistatin1b* and *gsx1*. Looking into our transcriptomic data, those genes were either found not to differ between the CSPG^+^ and CSPG^−^ groups or to be upregulated in the non-HB fraction. However, some of their homologues (i.e. *RASGEF1A*, *RAC1* and *FSTL1*) were upregulated in the chick HB cells. Likewise, *fgf20* and *etv5b*, which have been identified in the Rh centers of zebrafish, were either not detected in the hindbrain (*fgf20*) or actually upregulated in the HBs (*etv5b*). Nevertheless, the presence of several other FGFs in chick HB cells, along with the upregulation of several FGF downstream targets (*ETV1*, *ETV5*, *ETV4*, *DUSP6* and *SPRED1*), raises the intriguing possibility that the non-neurogenic function of Rh centers in teleost may have been lost in amniotes, while the HB zones have retained a similar role throughout evolution.

## MATERIALS AND METHODS

### Embryos

#### Chick

Fertile Loman chicken eggs (Gil-Guy Farm, Moshav Orot, Israel) were incubated at 37°C for 72-84 h until reaching the desired Hamburger Hamilton (HH) developmental stage of HH14 or HH18, as specified. A small hole was made in the shell through which 5 ml of albumin were removed using a syringe. Next, a small window was made in the shell to expose or harvest the embryo for further procedures (Kayam et al., 2013). All mice and chick procedures were approved by the Hebrew University Animal Care regulations (license number for mice studies 18-15452-1).

#### Mice

Wild-type mice (C57BL/6) were purchased from Harlan Laboratories (Rehovot, Israel). Mice were mated and females were examined for a vaginal plug the following morning; this was considered as embryonic day (E) 0.5. After 10.5 days, females were euthanized and embryos were taken for further procedures (Kalev-Altman et al., 2020). Mice were kept in the Hebrew University Specific Pathogen Free animal facility according to animal care regulations. All procedures were approved by the Hebrew University Animal Care Committee (license number 18-15452-1).

### Hindbrain primary cell culture experiments

#### Chick

Hindbrain regions of HH18 embryos were dissected in sterile PBS with penicillin-streptomycin (Pen-Strep, 1:100; Gibco, USA), then placed in a tube containing human embryonic stem cell medium [hESC; DMEM/F-12 1:1 with 20% KnockOut serum replacement, GlutaMax L-alanyl-L-glutamine (2 mM), non-essential amino acids (0.1 mM; all from Gibco), β-mercaptoethanol (0.1 mM; Sigma-Aldrich), Pen-Strep (1:100) and Fungizone (1:500)]. Media was next replaced with 1 ml of TrypLE Express (Gibco) to dissociate the tissue into single cells. After a manual disassociation by pipetting up and down, TrypLE was neutralized with 10:1 hESC medium and cells were passed through a 100 μm mesh strainer to detach adherent cells. Cells were cultured in hESC media at density of 1×10^5–6^ cells/ml, seeded in a 48- or 96-well Nunclon Delta Surface culture plate (Thermo Fisher Scientific) and incubated at 37°C in 5% CO_2_ (Peretz et al., 2016, 2018). For live imaging, cell plates were imaged every 3-6 h in IncuCyte S3 Zoom HD/2CLR time-lapse microscopy system, equipped with a 20× Plan Fluorobjective (Sartorius). Time-lapse movies were generated by capturing phase images for up to 5 days of incubation (Wang et al., 2020a).

#### Mouse

Cell cultures from E10.5 hindbrains were prepared and imaged as described above, with the following modifications: hindbrains were collected in PBS containing calcium and magnesium (Biological Industries, Israel), and cells were grown in hESC media combined with NeuroCult Proliferation Supplement, supplemented with 20 μg of human recombinant EGF, 10 μg human recombinant bFGF and 10 μg 0.2% heparin solution (all from STEMCELL Technologies).

#### Magnetic bead cell sorting of hindbrain cells

Cell separation was carried out using MACS MicroBeads cell separation system (Miltenyi Biotec) according to the manufacturer's protocol, with slight adjustments. Briefly, 60 hindbrains of HH18 chick embryos were harvested and disassociated into single cells using collagenase type 4 (200 units/ml, Worthington 47B9407). Cells were then centrifuged at 600 ***g*** for 10 min, washed in PBS and re-centrifuged. Next, cells were incubated with mouse anti-CSPG antibody (c8053; Sigma-Aldrich) diluted 1:50 in MACS BSA Stock Solution and autoMACS Rinsing Solution (1:20, Miltenyi Biotec) for 1-2 h at room temperature. Next, cells were centrifuged and washed in PBS twice, then incubated with anti-mouse IgG micro-beads (1:10 in autoMACS Running Buffer, Miltenyi Biotec) for 30 min at 4°C. Cells were then washed and moved into MACS cell separation magnetic columns placed on MACS iMAG separator, allowing the CSPG^+^ cells to attach to the column, while the CSPG^−^ fraction passed through and was collected. The CSPG^+^ cells were finally eluted from the column by removal of the magnetic field and collected separately. The separated CSPG^+^ and CSPG^−^ cells fractions were centrifuged, suspended in hESC medium, plated to generate a culture, and grown and imaged as described above. Validation of the proper separation into CSPG^+^ and CSPG^−^ cell fractions was carried out using flow cytometry analysis, as described below.

#### Treatments

Inhibition of CSPG through digestion of its CS chains was carried out by adding 50 mU/ml Chondroitinase ABC (ChABC; Sigma-Aldrich) diluted in 0.01% BSA (Sigma-Aldrich). Addition of external CSPG was achieved using 50 mg/ml proteoglycan from bovine nasal septum (Sigma-Aldrich), diluted in molecular grade water. Both treatments were added to the culture media every 48 h. As controls, cells were treated similarly with 0.01% BSA or water.

### *In vivo* experiments

#### Plasmid electroporation

pcDNA3.1-chABC and pcDNA3.1-GFP plasmids (Muir et al., 2010) were mixed 2:1. For control, pcDNA3.1-GFP plasmid was mixed with molecular grade water in a 2:1 ratio. Plasmids were injected into the hindbrain lumen of HH14 embryos using a pulled glass capillary, as previously described (Kohl et al., 2013). L-bent gold electrodes (1 mm diameter) were placed flanking the hindbrain and an electrical current of 25 V was applied in five pulses of 45 ms with a pulse interval of 300 ms using ECM 830 electroporator (BTX). After electroporation, PBS was applied over the embryos, and eggs were sealed with parafilm and re-incubated at 37°C for additional 24 h before harvesting. Embryos were then either fixed for immunofluorescence staining or their hindbrains were removed to generate cell culture, as described in the section ‘Chick’.

#### ChABC injection

ChABC (50 mU/ml) was diluted in 15% Pluronic F127 thermosensitive hydrogel (Sigma-Aldrich) and injected locally into the hindbrain lumen of HH14 embryos *in ovo*, using a pulled glass capillary. As a control, embryos were treated with 0.01% BSA. After injection, embryos were re-incubated overnight at 37°C, allowing the hydrogel to solidify, hence assuring a prolonged exposure of the area to the substance (Pokhrel et al., 2022; Shriky et al., 2020). Post incubation, embryos were harvested and hindbrains were removed.

#### CM-DiI labeling

HH15 embryos were placed in a petri dish containing PBS with their roof plate open. CM-DiI (C-7000, Molecular Probes) was dissolved in 100% ethanol to reach a concentration of 1 mg/ml, then further diluted in DMSO to a working concentration of 10 μg/ml. CM-DiI was manually applied to local HB cells using a pooled glass capillary under a stereoscope for accurate detection of the HBs. Immediately after labeling, hindbrains were used to generate primary cell cultures, as described in the sections ‘Chick’ and ‘Treatments’.

### Flow-cytometry

Whole hindbrains dissected from HH18 chick embryos, or 5-day-old primary cultures, treated as mentioned above, were incubated in Express TrypLE for 10 min at 37°C, then dissociated manually and neutralized with 1:10 hESC medium. Cells were then fixed in 4% paraformaldehyde solution (PFA; Sigma-Aldrich) for 10 min at room temperature, centrifuged at 600 ***g*** for 10 min, washed in PBS for 5 min and centrifuged again. Cells were next incubated in blocking solution (0.2% Triton in PBS and 2% goat serum) for 1 h at room temperature, followed by incubation for 2 h at room temperature or overnight at 4°C in 1% BSA with primary antibodies (1:300). After washes and centrifugation, cells were incubated for 2 h at room temperature in 1% BSA with the appropriate Alexa-Fluor secondary antibody (1:300; Life Technologies) dissolved in 0.5% BSA. Next, cells were centrifuged, washed and centrifuged again, as described above. Finally, cells were suspended in clean PBS and passed through an Accuri C6 Flow Cytometer (BD Biosciences). Flow cytometry analysis was performed using BD Accuri C6 software. For validation of the immunomagnetic separation according to CSPG expression levels, CSPG^+^ and CSPG^−^ cells were collected and centrifuged for 3 min at 14,000 ***g***, then subjected to the same procedure. Detection of cell death due to exposure to ChABC was achieved using Annexin V-FITC Early Apoptosis Detection Kit (Cell Signaling), carried out according to the kit user guide with minor modifications. Briefly, treated and control hindbrains were dissociated and prepared as described above, then resuspended in 300 µl 1× Annexin V Binding Buffer. Next, cells were added with Annexin V-FITC conjugate (1:100) and propidium iodide (1:30) and incubated on ice for 10 min. Finally, 100 µl of 1× Annexin V Binding Buffer was added to the cells to terminate the reaction and cells were taken for FACS analysis.

### Flow-cytometry cell sorting

Thirty hindbrains of HH18 chick embryos were harvested and dissociated into single cells using collagenase type 4 (200 units/ml, Worthington 47B9407). Cells were then centrifuged at 600 ***g*** for 10 min, washed in PBS, then centrifuged again. Next, cells were incubated with mouse anti-CSPG antibody (c8053, Sigma-Aldrich) diluted 1:50 in MACS BSA Stock Solution and autoMACS Rinsing Solution (1:20, Miltenyi Biotec) for 75 min at room temperature. Next, cells were centrifuged and washed in PBS twice, then incubated with anti-mouse Alexa-Fluor 488 antibody (1:200, Thermo Fisher Scientific) in autoMACS Running Buffer (Miltenyi Biotec) for 30 min at room temperature. Cells were then washed, centrifuged, resuspended and kept in hESC media overnight at 4°C. Next, cells were washed with autoMACS Running Buffer and stained with DAPI (1:200 in autoMACS Running Buffer) for 5 min at room temperature, then washed again. 1×10^7^ cells/ml were passed to FACS tubes and sorted using an ARIA III FACS (BD Biosciences) into 1 ml autoMACS Running Buffer. The gating was set according to size and granularity using FSC and SSC to capture singlets and remove debris. The cut-off for sorting the positive (CSPG^+^) and negative (CSPG^−^) cells was based on Alexa-Fluor 488 stained or unstained cells, appropriately, with the exclusion of dead and/or damaged DAPI^+^ cells, chosen by manual gating.

### Immunofluorescence

#### Whole mount staining

Chick or mouse embryos were harvested at HH18 or E10.5, respectively, cleaned from surrounding membranes and fixed in 4% PFA (Sigma-Aldrich) overnight at 4°C. Embryos were next washed with PBS and incubated in blocking buffer [0.1% Tween 20 in PBS (PBT) with 5% goat serum] (Biological Industries) for 2 h. Next, embryos were incubated overnight at 4°C in blocking solution with the following primary antibodies: rabbit anti-Sox2 (1:400; Millipore), mouse anti-CSPG and mouse anti-Map2 (1:80 and 1:200, respectively, Sigma-Aldrich), rat anti-DSD-1 (1:200; Sigma-Aldrich), rabbit anti-FGF8 (1:200; Thermo Fisher Scientific), mouse anti-Tuj1 and mouse anti-HuC/D (both 1:400; Abcam); rabbit anti-MMP2 and rabbit anti-MMP9 (both 1:100, Abcam), mouse anti-Pax2 (1:50, Abcam), goat anti-doublecortin (1:200, Santa-Cruz Biotechnology) and mouse anti-Islt1, anti-Lhx1, anti-Lhx5, anti-laminin C1, anti-Tag1 and anti-HSPG (all 1:50, DSHB, USA).

After washes, embryos were incubated for 2 h at room temperature with the following secondary antibodies: goat anti-mouse Alexa488, goat anti-mouse Alexa594, goat anti-rabbit Alexa488 and goat anti-rabbit Alexa594 (1:300; diluted in blocking solution; Life Technologies). Next, embryos were washed with PBS and incubated for 15 min at room temperature in PBS with DAPI (1:400; Sigma-Aldrich). After washes with PBS, hindbrains were finally dissected and flat-mounted on slides with FluoroGel with Tris buffer mounting medium (Electron Microscopy Science) (Kohl et al., 2012).

#### Staining in frozen sections

Chick embryos were fixed as above and then incubated overnight in 30% sucrose and PBS at 4°C. Embryos were embedded and frozen in Optimal Cutting Temperature compound (Sakura Finetek) in fitting cryomolds. Blocks were sectioned at 13 µm using a CM1860 cryostat (Leica). Immunostaining was performed as described above.

#### Cell culture staining

Cultured media were removed and cells were fixed with 4% PFA for 30 min at room temperature. Wells were rinsed with PBS for 10 min and incubated in blocking solution (5% goat serum in 0.05% PBT) for 2 h at room temperature. Primary antibodies (as mentioned above) were diluted in blocking solution and added to each well overnight at 4°C. Wells were washed with PBS three times and incubated for 2 h at room temperature with appropriate Alexa-Fluor secondary antibodies, as described above. Wells were re-washed and incubated with DAPI (1:400) for 15 min, washed again and kept in PBS until imaged.

### *In situ* hybridization

Whole-mount *in situ* hybridization was performed on HH18 chick embryos as previously described (Weisinger et al., 2008), using digoxigenin-UTP (DIG)-labeled probes for chick Atoh1 (Cath1), Delta1, FGF3, Mkp3, MMP16, Msx1 and Pax7 (Ben-Yair and Kalcheim, 2005; Kohl et al., 2012; Myat et al., 1996; Roth et al., 2017; Sela-Donenfeld et al., 2009; Weisinger et al., 2008, 2010, 2012). DIG-labelled probes were detected using 1:2000 alkaline phosphatase-coupled antibody followed by NBT/BCIP staining (Roche).

### Transcriptomics

#### Library preparation and sequencing

mRNA was extracted from six biological replicates of FACS-isolated hindbrain cells, as described above, using Single Cell RNA purification kit (Norgen Biotek), according to the manufacturer's protocol. Each RNA sample had a RIN>6.9. Libraries were prepared by the Center for Genomic Technologies at the Hebrew University of Jerusalem, using the KAPA Stranded mRNA-Seq Kit (KR0960, Roche) and Illumina platforms sample preparation protocol (v3.15) according to the manufacturer's protocol. RNA sequencing was conducted on an Illumina NextSeq 2000 machine using NextSeq 2000 P2, 100 cycles kit (Illumina). The output was ∼25 million single end-120 bp reads per sample.

#### Bioinformatic analysis

Reads in fastq format were created with bcl2fastq v2.20.0.422, inspected for quality issues with FastQC, v0.11.8 and quality-trimmed with cutadapt, v3.4, for removal of adapters, polyA and low-quality sequences, as previously described (Alfi et al., 2021). Based on these quality analyses, reads from four biological replicates of the CSPG^+^ group and six biological replicates of the CSPG^−^ group were further analyzed by alignment to the chicken transcriptome and genome with TopHat, using genome version GRCg6a with annotations from Ensembl release 99. Quantification was caried out with htseq-count, v0.13.5. Differential gene expression analysis was performed using the R package DESeq2, v1.30.0 (Love et al., 2014). Genes with a sum of raw counts less than 10 over all samples were filtered out, then normalization and differential expression were calculated. Comparing CSPG^+^ samples with CSPG^−^ samples was tested with default parameters using a significance threshold of *P*adj<0.05. Whole differential expression data were subjected to gene set enrichment analysis using GSEA (Subramanian et al., 2005) (cutoff independent) in order to determine whether *a priori* defined sets of genes show statistically significant concordant differences between the two biological states. We used the hallmark and Gene Ontology Biological Process (GO) gene sets collections, all taken from the molecular signatures database MSigDB (Subramanian et al., 2005). A plot of protein-protein interaction networks was generated, showing the interaction network between functionally important genes in the CSPG^+^ versus CSPG^−^ groups. The interaction information was obtained from IPA (Ingenuity Pathway Analysis, Qiagen; https://digitalinsights.qiagen.com/products-overview/discovery-insights-portfolio/analysis-and-visualization/qiagen-ipa/) and the STRING database (Szklarczyk et al., 2023). The figure was generated using Cytoscape (Shannon et al., 2003). RNA-seq data reported in this paper have been deposited in GEO under accession number GSE230804.

### Real-time PCR

mRNA was extracted from whole hindbrains or 5-day-old CSPG^+^ cultures, treated as described in the section ‘Treatments’, using a Single Cell RNA Purification Kit (51800; Norgen Biotek) according to the kit protocol, along with Norgen's RNase-Free DNase I Kit to degrade remaining DNA. cDNA was prepared using a High-Capacity cDNA Reverse Transcription kit (Thermo Fisher Scientific), according to the manufacturer's instructions. Real time (RT)-PCR was performed using Fast SYBR Green PCR Master Mix reagent (Thermo Fisher Scientific) with the following reverse and forward primers: GAPDH Fwd AGATGCAGGTGCTGAGTATG, Rev CTGAGGGAGCTGAGATGATAA; Sox2 Fwd TTAAGTGAAGGCGTGCTGC, Rev CCTCCTATCACTGCACCTTC; TUBB3 Fwd GACCGCATCATGAACACTTTC, Rev CGTGTTCTCCACCAGTTGAT; MAP2 Fwd CCTCCTAAATCTCCAGCAACTC, Rev CCCACCTTTAGGCTGGTATTT. 2 µl of cDNA were mixed with 10 µl SYBR mix, 7 µl deionized H_2_O and 1 µl of the selected primers. Real-time PCR amplification was performed using the following program: 95°C for 5 min, 40 cycles of 95°C for 15 s, then 60°C for 45 s. Results were normalized to GAPDH and analyzed using StepOne Software v2.2.2 (Applied Biosystems) using ΔΔCT.

### Imaging

#### Scanning electron microscopy

Chick and mouse embryos (HH18 and E10.5, respectively) were harvested and fixed for 1 h at room temperature with 2% PFA and glutaraldehyde in 0.1 M phosphate buffer pH 7 and 1% sucrose (all from Sigma-Aldrich). Next, the hindbrains were removed and placed on a coverslip coated with poly-l-lysine (Sigma-Aldrich). Samples were dehydrated in increasing ethanol concentrations (20%, 50%, 70%, 90% and 95%), then washed four times in 100% ethanol. The hindbrain samples were then moved to a Critical Point Dryer (Quorum K850) and were coated with gold in a gold sputter coating unit (Quorum Technologies). Samples were observed by low-vacuum scanning electron microscopy (SEM; JSM 5410 LV, Jeol). For correlative SEM-confocal analysis, hindbrains were taken to immunofluorescence staining as mentioned above, before the dehydration step. Hindbrains were first imaged using a confocal microscope (Zeiss LSM-510, with Argon-Ion and 2 He-Ne Lasers), then processed for SEM preparation.

#### Light and confocal microscopy

Flat-mounted hindbrains and cell cultures were imaged under an Axio Imager M1 microscope with AxioCam Mrm camera (Zeiss) or a CTR 4000 confocal microscope with DFC300FXR2 camera (Leica). *Z*-stack images were generated using Leica Microsystems software. For cell cultures and time-lapse analysis, an IncuCyte S3 with CMOS camera (Sartorius) was used, as previously described.

### Data analysis and statistics

Fluorescence quantification was performed using ImageJ, by subtracting the background reading out of the relative fluorescent area (corrected total cell fluorescence). For quantification of fluorescence in HBs versus Rhs, each data point represents an average value of four HBs and Rhs of each embryo (*n*=6 for mouse, *n*=7 for chick). For quantification of fluorescence in ChABC-treated hindbrains, each bar represents average fluorescence of an area within Rhs 3-5 in treated and control embryos (*n*=13-20 for inhibitor injection, *n*=20-28 for plasmid electroporation). Analysis of CSPG-Sox2 proximity was carried out using Microscopy Image Analysis Software 9.0.2 (IMARIS; Oxford Instruments). Confocal files were uploaded as 3D stacks into the software, then subjected to a three-step workflow. First, Sox2^+^ cells were marked with the ‘surface’ module, defined by the estimated size of a hindbrain cell. Next, CSPG spots were segmented with the ‘spots’ module. Finally, the CSPG^+^ spots were linked with the surface (Sox2^+^ cells) with the ‘spots-to-surface coloc.’ script, while the inclusion criteria were set as distance between spot and surface≤5 μm. The recorded protocol was applied for the rest of the dataset (*n*=7). The CSPG ‘spots’ were also used to generate a scatter plot, as each spot was assigned to its *z* dimension. Characterization of electroporated GFP^+^ cells in cryosections was performed in HB regions only, chosen based on the expression pattern of Sox2. For each embryo, analysis of HB cells in various regions was conducted. GFP^+^ cells in each image were assigned a score from 3 to 0 (3=a high quantity of cells, 2=some cells, 1=few cells and 0=no cells), reflecting the abundance of cells in three states: typical apical-basal polarity, apically abscised and radially migrating cells, and mantle-positioned cells and/or axons. The percentage of each phenotype was calculated for individual embryos and then averaged for each treatment (*n*=8 for ChABC and *n*=5 for control; at least 15 sections per embryo). Cell culture analysis for neurite length, eccentricity, and floating and adherent spheres was performed using the IncuCyte S3 live imaging system software (Sartorius). Each data point represents an average calculated in one or several wells at day 5 of incubation from different experimental replicates, as stated in the figure's legend. Quantification of phase and color cell count to calculate percentage of cells and co-expression was also carried out using the IncuCyte S3 live imaging system software. Monolayer quantification was performed using Ilastik software integrated with ImageJ analysis. Statistics were performed by unpaired *t*-test or one-way ANOVA followed by a Tukey's post-hoc test using Graphpad Prism 8 software. *P*<0.05 was considered significant; data are displayed as mean±s.d.

## Supplementary Material



10.1242/develop.201934_sup1 Supplementary information
