# Supplementary material for: Hindbrain boundaries as niches of neural progenitor and stem cells regulated by the extracellular matrix proteoglycan chondroitin sulphate
Source: Development. 2024 Feb 13;151(4):dev201934. doi: 10.1242/dev.201934 (PMC10911165; doi:10.1242/dev.201934)
Supplement: Supplementary information [file develop-151-201934-s1.pdf]

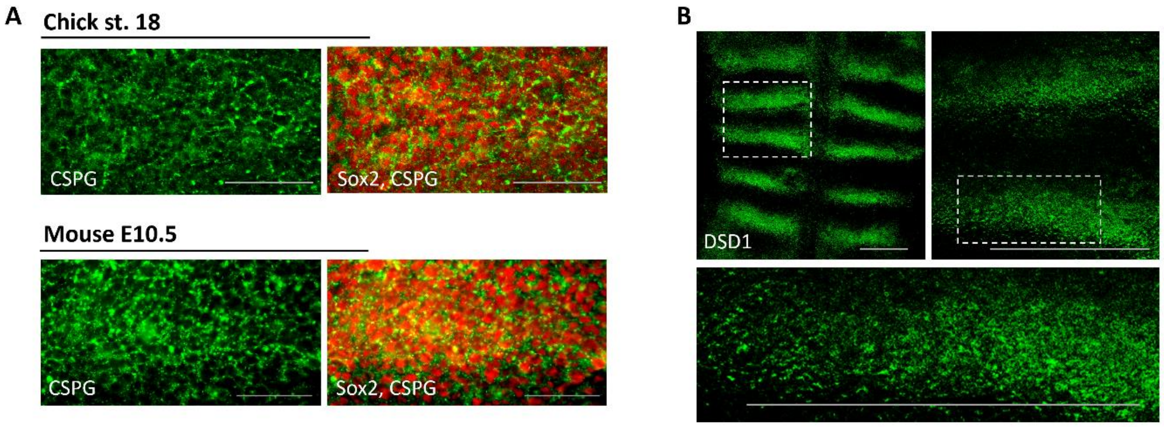

Fig. S1. Membranal staining of CSPG and DSD1 surrounding Sox2+ cells in the hindbrain boundaries. (A): Confocal images of magnified HB regions from st.18 HH chick and E10.5 mouse embryos, immunostained for CSPG (green) and Sox2 (red). **(B):** Confocal images of st.18 chick hindbrain immunostained for DSD1. High-magnification of boxed areas showing membranal staining in the HBs. Scale bar in A= 50 µm, in B= 200 µm. HB=hindbrain boundaries.

Development • Supplementary information

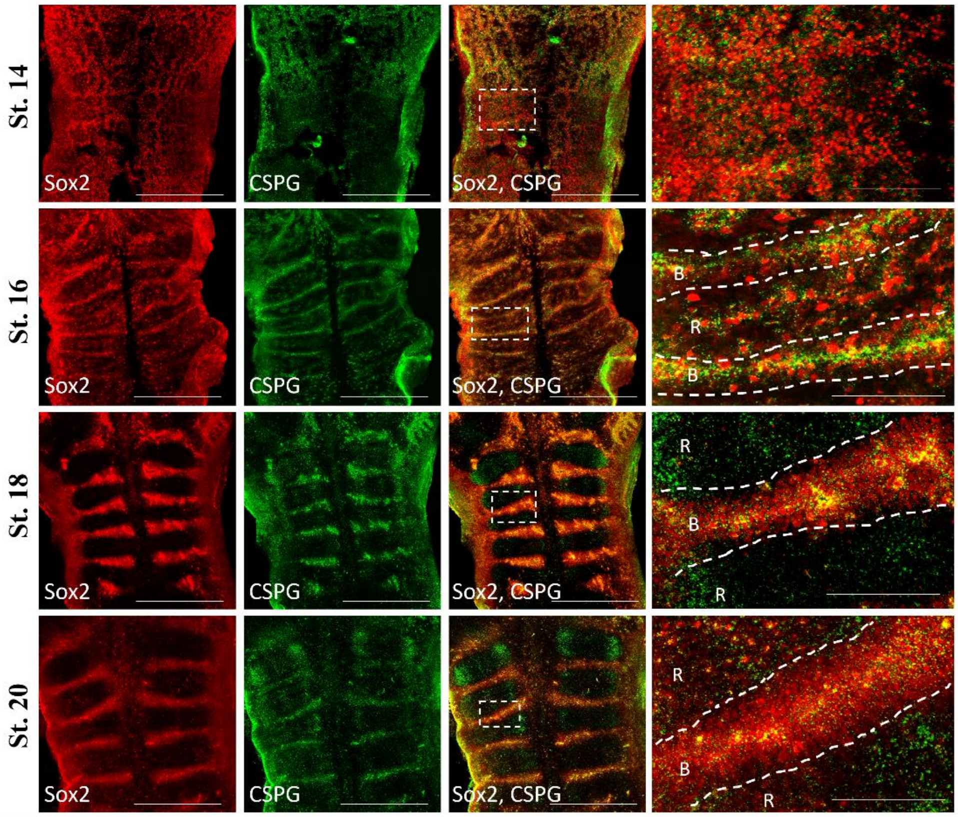

Fig. S2. Spatiotemporal analysis of Sox2 and CSPG co-expression in the hindbrain. Confocal images of st.14-20 HH chick hindbrains immunostained for Sox2 and CSPG. High-magnification of boxed areas are presented to the right. Dashed lines define boundaries (B) and rhombomere (R) areas. Scale bar = 500 µm, 100 µm for higher magnification.

Development • Supplementary information

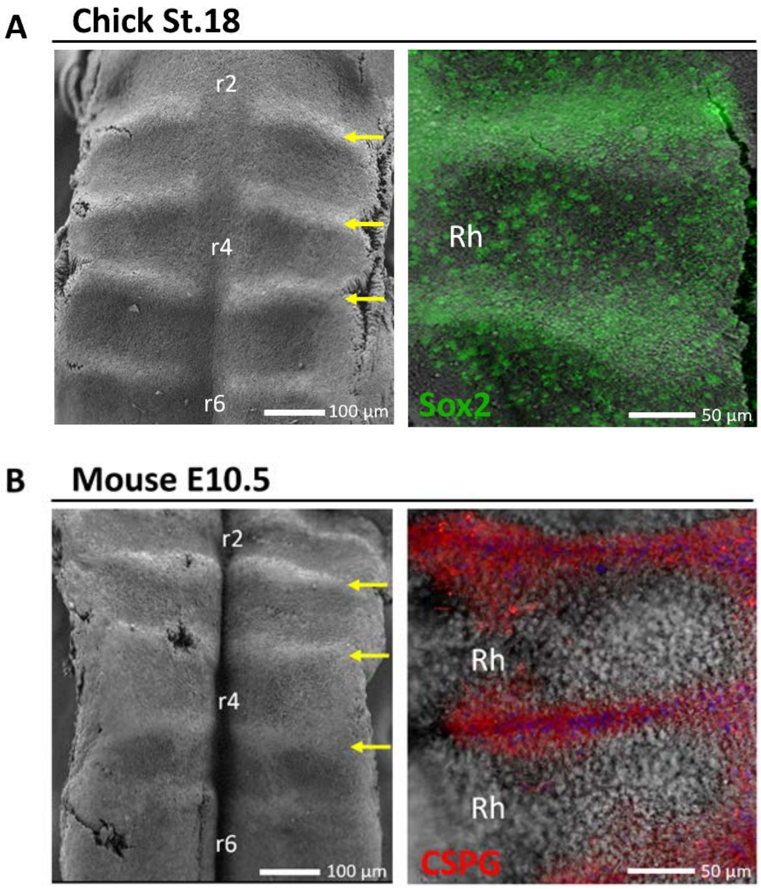

Fig. S3. Scanning electron microscopy analysis of chick and mouse hindbrains. (A,B): X200 magnification of SEM images of chick and mouse flat-mounted hindbrains, showing the boundaries as elevated ridges (arrows) and rhombomere grooves. Correlative light and scanning electron microscopy showing correlation between the elevated HBs regions and Sox2 (green) or CSPG (red) expression presented to the right. Arrows mark HBs. RH/r=rhombomere.

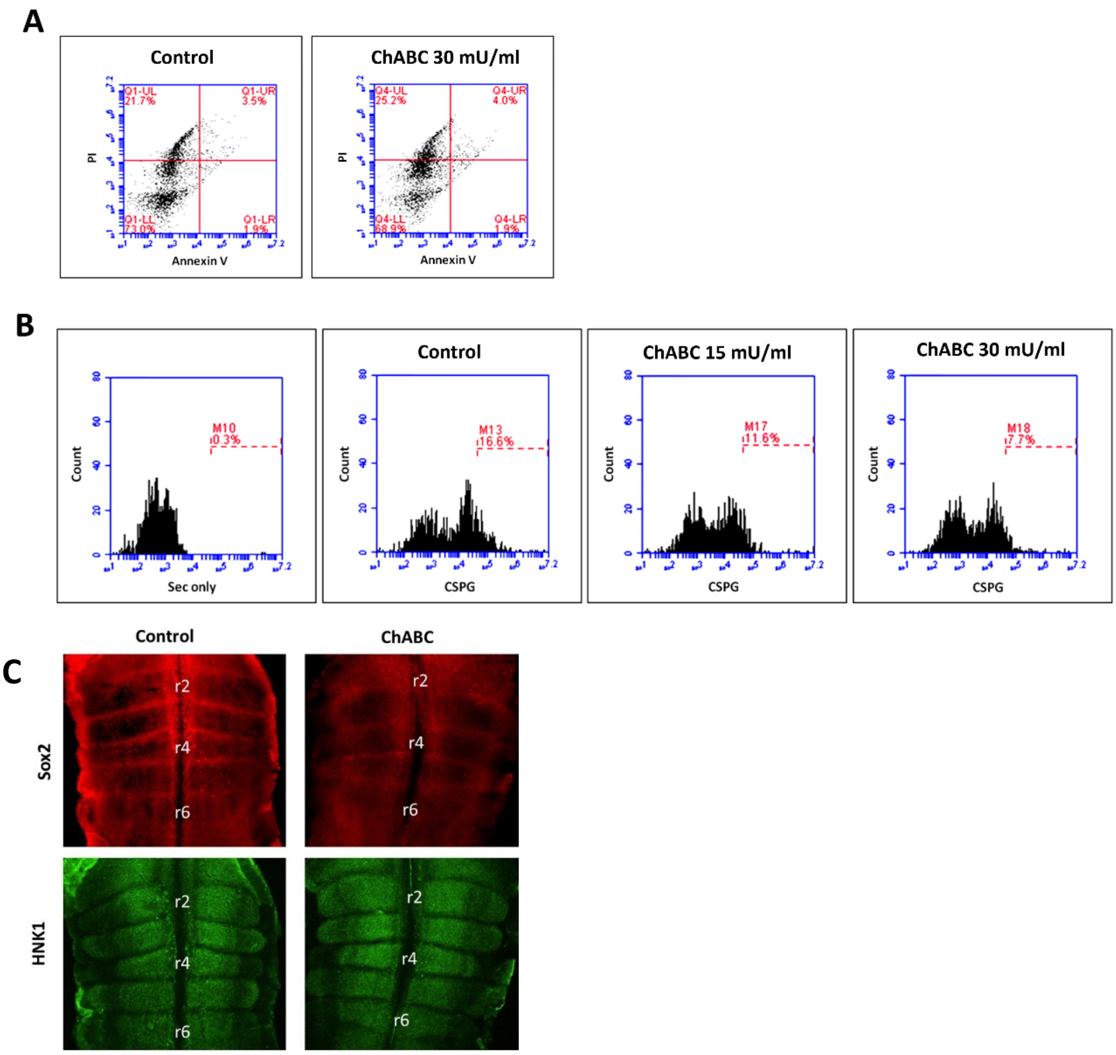

Fig. S4. Evaluation of the effect of ChABC on hindbrain cell death, segmentation and CSPG levels in vivo. (A): Flow-cytometry analysis of Annexin V and PI staining in control and ChABC-treated chick hindbrains. Cells positive to Annexin V and negative or positive to PI are either undergoing apoptosis or are dead (LR and UR in both panels). Percentage of Annexin V+/ PI+ cells (in red) similar in both cell groups **(B):** Flow-cytometry analysis of CSPG+ cells, showing staining of secondary antibody (Sec only) used for gating of cells positive for CSPG, and the ratio of CSPG+ cells in control and ChABC-treated hindbrains in two concentrations. Percentage of CSPG+ cells appear in red. LR= lower right; UR= upper right. **(C):** Flat mounts views of control and ChABC-treated embryos co-immunostained for Sox2 (red) and HNK1 (green). Sox2 expression is reduced at hindbrain boundaries upon ChABC-treatment, whereas the gross segmental organization of the hindbrain, as marked by HNK1, remains unaffected. r= rhombomere.

Development • Supplementary information

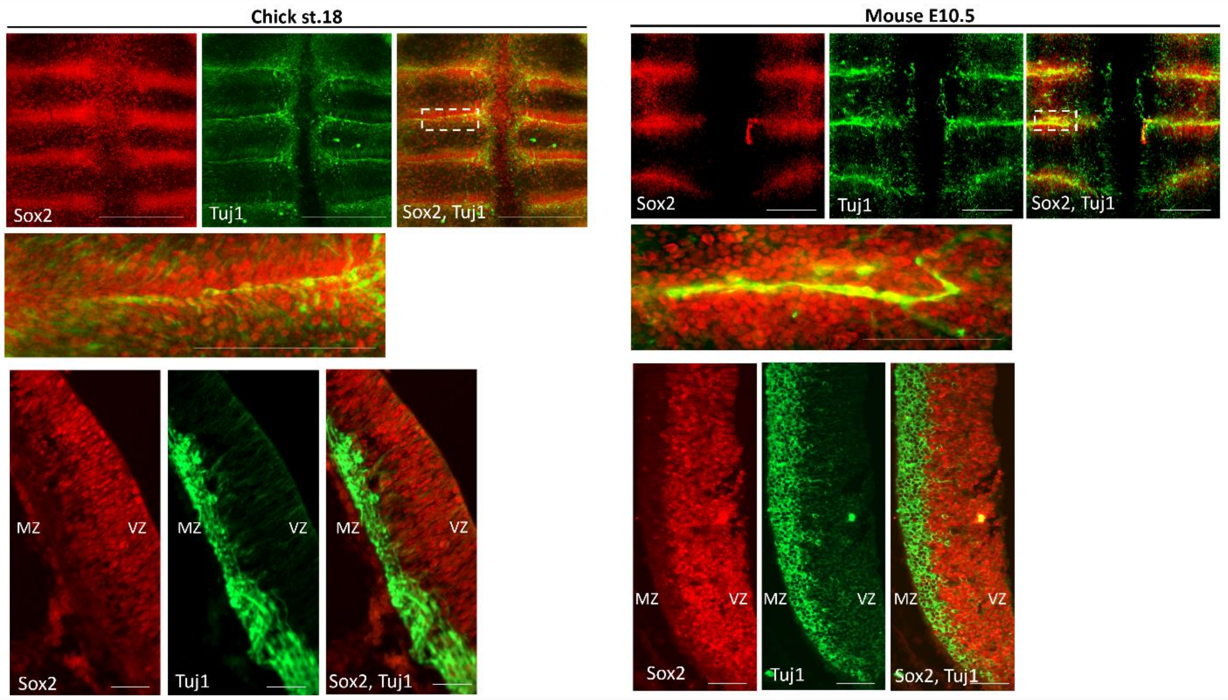

Fig. S5. Co-expression of Sox2 and Tuj1 in chick and mouse hindbrains. Flat-mounted view of st.18 chick and E10.5 mouse hindbrains (upper panels) and representative transverse sections of HBs (lower panels), immunostained for Sox2 and Tuj1. High-magnification of boxed areas showing co-staining of Sox2/Tuj1 are presented underneath each set of images. Scale bar = 100 µm for flat mounts, 50 µm for sections. VZ= ventricular zone; MZ= mantle zone.

Development • Supplementary information

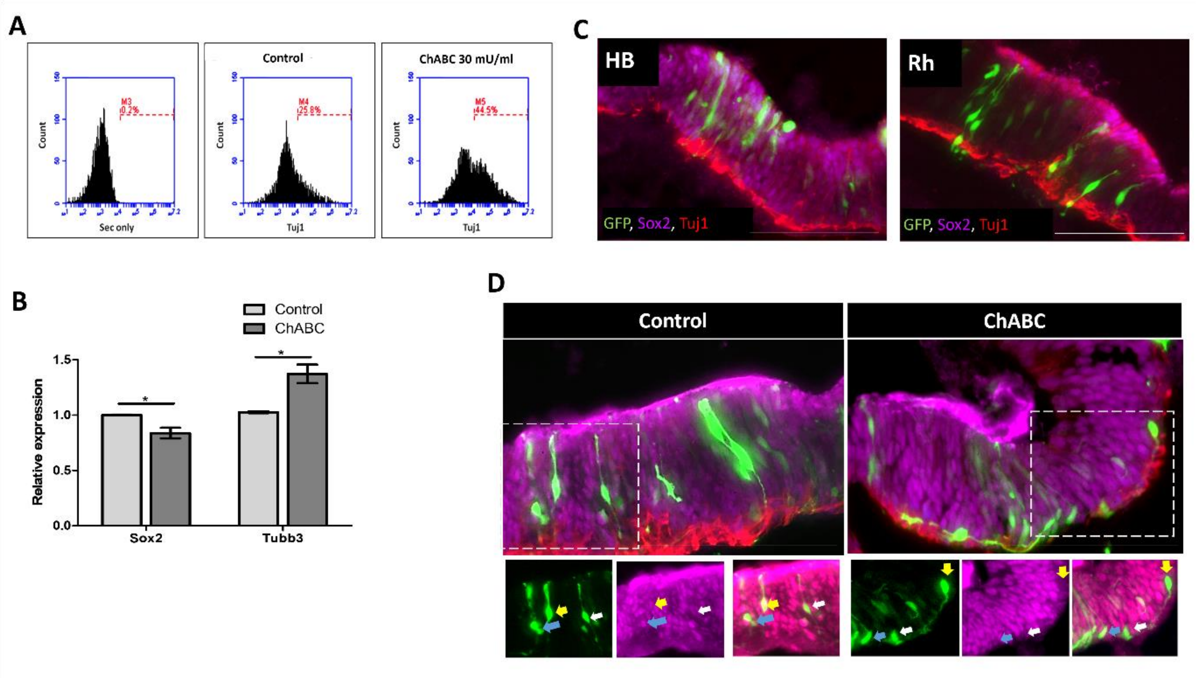

Fig. S6. The effect of ChABC on Sox2 and Tuj1 expression. (A): Representative flow-cytometry analysis of Tuj1+ cells in control and ChABC-treated hindbrains (n=12 hindbrains in each group), showing an increase in Tuj1+ cells upon CSPG-loss **(B):** Real-time RT-PCR analysis of *Sox2* and *Tubb3 (*Tuj1*)* expression in control and ChABC-treated hindbrains, showing downregulation of Sox2 and upregulation of Tuj1 upon CSPG-loss. Data are mean±s.d. from 3 experimental replicates; n=12 hindbrains in each group (two-tailed unpaired t-test). **(C):** Confocal images of transverse sections obtained from an hindbrain electroporated with pcDNA3.1-GFP plasmid (green) and immunostained for Sox2 (magneta) and Tuj1 (red). Classification of images as representing HBs/Rhs was done based on the span of Sox2 expression along the V-M zone, which shows broad Sox2 expression at the HBs, whereas much less expression is seen at the Rh. (**D)** Confocal images of transverse sections of HBs electroporated with pcDNA3.1-GFP or ChABC-GFP plasmids (green) and immunostained for Sox2 (magenta). Boxed areas are magnified in the panels bellow which present the green, magena and merged channels, respectively. Each colored arrow marks the same cells at the different channels, indicating that control-GFP cells are also Sox2-positive, whereas ChABC-GFP cells are Sox2-negative. * $P< 0.05$. Scale Bar= 100 µm. HB= hindbrain boundary; Rh= rhombomere.

Development • Supplementary information

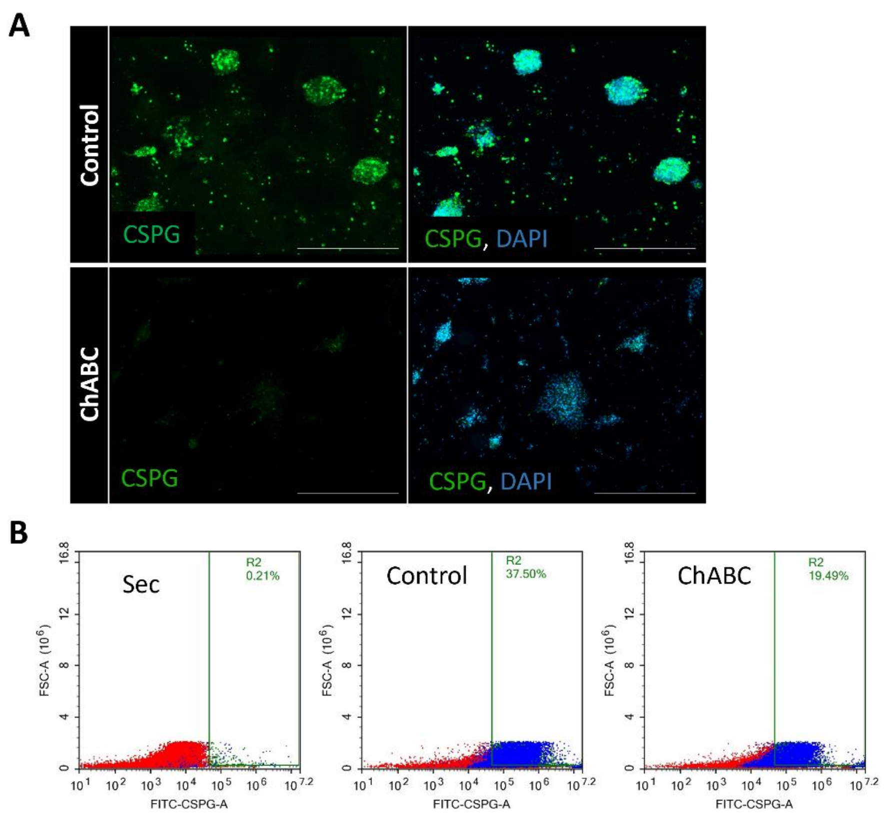

Fig. S7. Validation of the inhibitory effect of ChABC in vitro. **(A):** Confocal images of primary cultures on day 5 of incubation that were obtained from st.18 chick hindbrains, treated with BSA (control) or ChABC and immunostained for DAPI and CSPG (green). CSPG expression is weakened upon ChABC-treatment. **(B):** Flow-cytometry analysis of CSPG+ cells in the control and ChABC-treated cultures at day 5 of incubation, prepared as described above. Staining of secondary antibody (Sec) was used for gating of cells positive for CSPG (left). The number of CSPG+ cells in control and ChABC-treated cells is shown at the right panels. Percentage of CSPG+ cells appear in green. Scale bar= 50 µm.

Development • Supplementary information

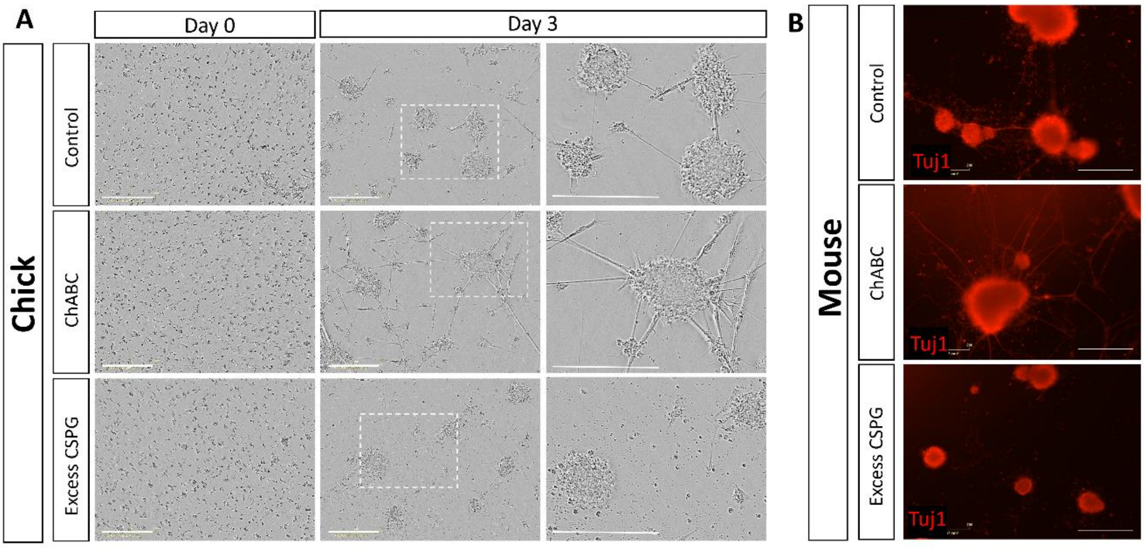

Fig. S8. Time-lapse analysis of chick and mouse hindbrain cells during modifications in CSPG levels in vitro. (A): Single bright-field images from time lapse analysis of primary cell culture of st.18 chick hindbrains on day 0 and day 3 of incubation. Cultures were treated with BSA (control), ChABC or excess CSPG. Higher-magnifications of boxed areas are represented to the right. **(B)**: Fluorescent images of primary cell culture of E10.5 mouse hindbrains on day 5 of incubation following treatment with ChABC or excess CSPG, immunostained for Tuj1. Scale bar = 200 µm.

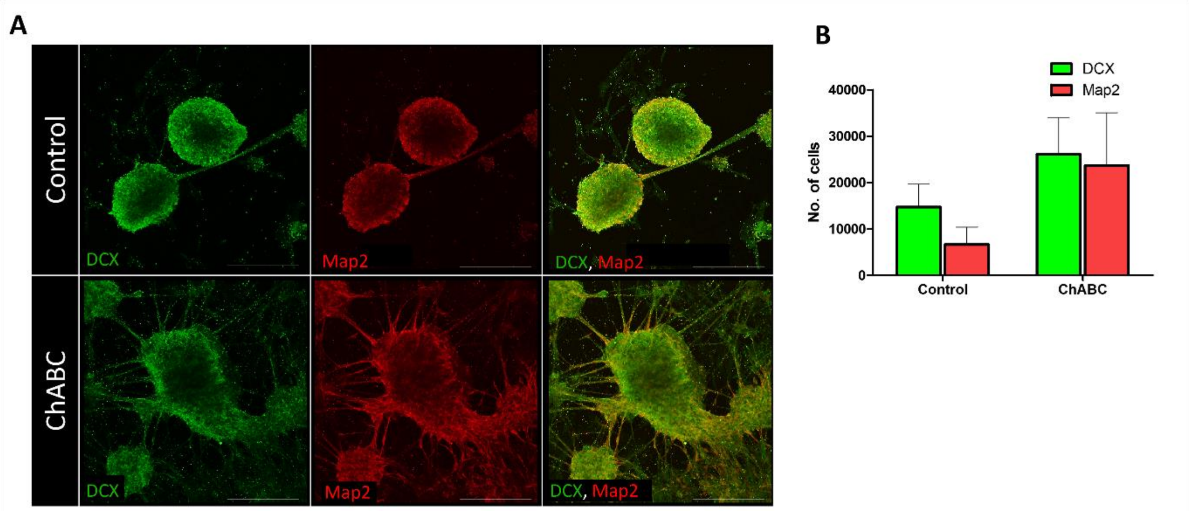

Fig. S9. Expression of early and late markers of neural differentiation in hindbrain-primary cell cultures. **(A)**: Confocal images of primary cultures on day 5 of incubation that were obtained from st.18 chick hindbrains, treated with BSA (control) or ChABC and immunostained for DCX and Map2. **(B):** Quantification of the number of DCX+ or Map2+ expressing cells in the control and ChABC-treated cultures. n=9 wells for each group from 3 experimental replicates. Data are mean±s.d. Scale bar = 200 µm.

Development • Supplementary information

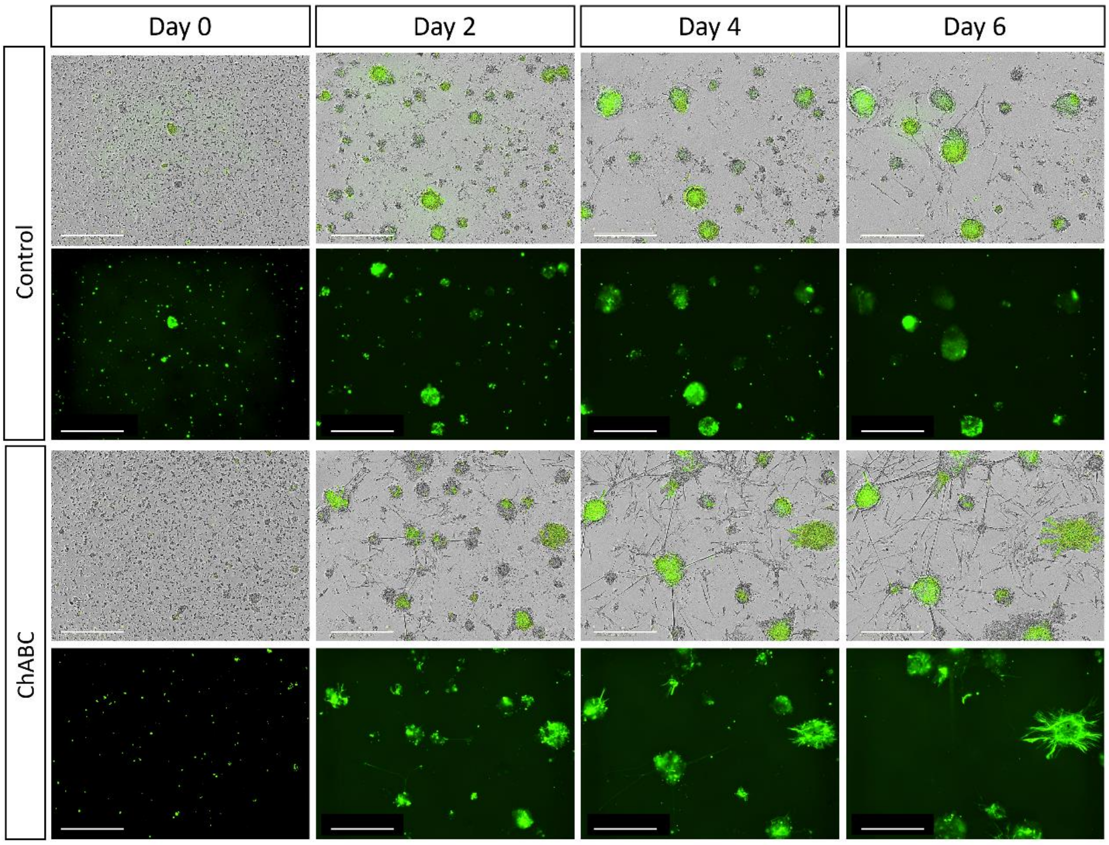

Fig. S10. Time-lapse analysis of chick hindbrain cells electroporated with control-GFP or ChABC-GFP plasmids. Single images from time lapse analysis of primary cell cultures prepared from st.18 chick hindbrains. Images from days 0,2,4,6 3 of incubation, following electroporation with pcDNA3.1-GFP (control) or pcDNA3.1-ChABC plasmids, are presented. Top panels are of merged bright-field + fluorescence channels, and lower panels are of fluorescence channel. Scale bar = 400 µm.

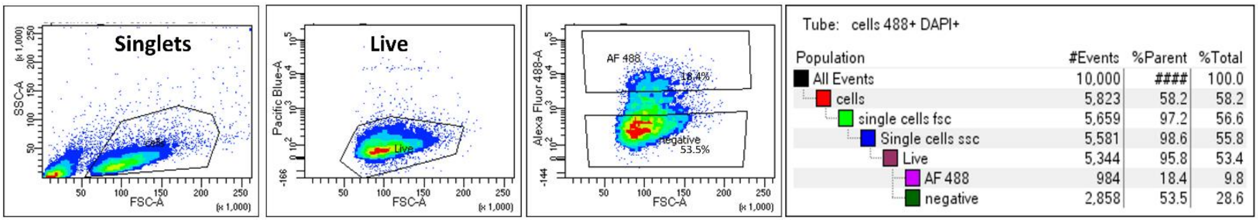

Fig. S11. FACS purification of CSPG-labeled cells from hindbrains of St.18 chick embryos stained with CSPG and AlexaFlour-488 secondary antibody, separated into CSPG+ and CSPG- cells. Gating was done according to singlets out of all events (5659 cells), live cells (DAPI^{+}/PI) out of all singlets (5344 cells), CSPG^{+} cells (AF 488; 984 cells) and CSPG^{-} cells (2858 cells) out of all live cells.

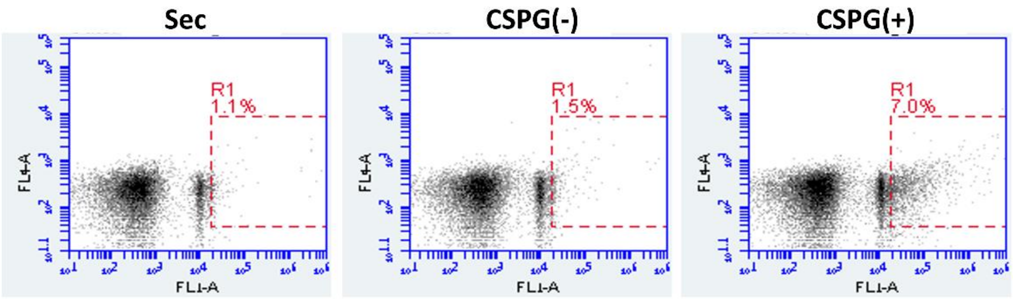

Fig. S12. Validation of magnetic column-based separation of CSPG+ cells. Flow-cytometry analysis of CSPG enrichment in CSPG- and CSPG+ cell fractions following magnetic bead cell sorting, showing staining of secondary antibody (Sec) used for gating of cells positive for CSPG, and the percentage of CSPG+ cells in the separated cell groups. Percentage of CSPG+ cells appear in red.

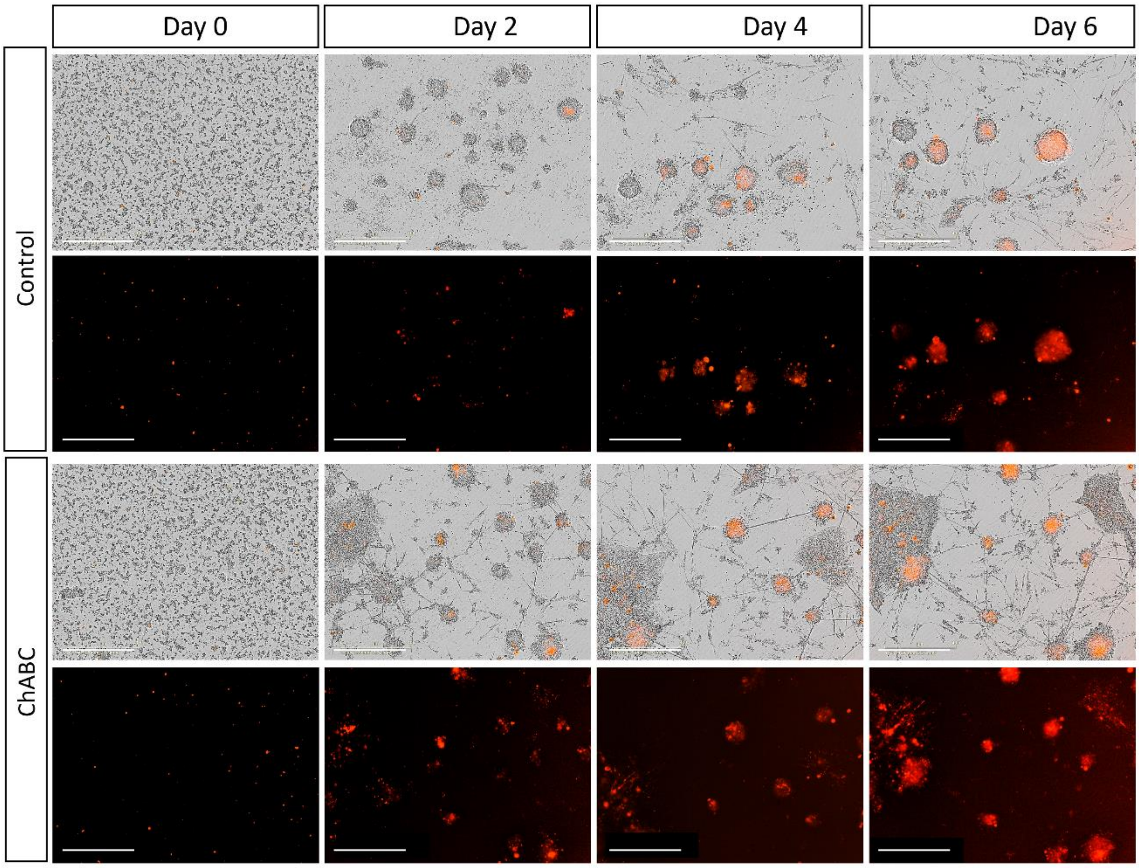

Fig. S13. Time-lapse analysis of chick hindbrain cells with HB cells labeled with CM-DiI. Single phase/red or just red channel images from time lapse analysis of primary cell culture of st.18 chick hindbrains on day 0,2,4,6 of incubation, following manual labeling of HB regions with CM-DiI (red). Scale bar = 400 μm.

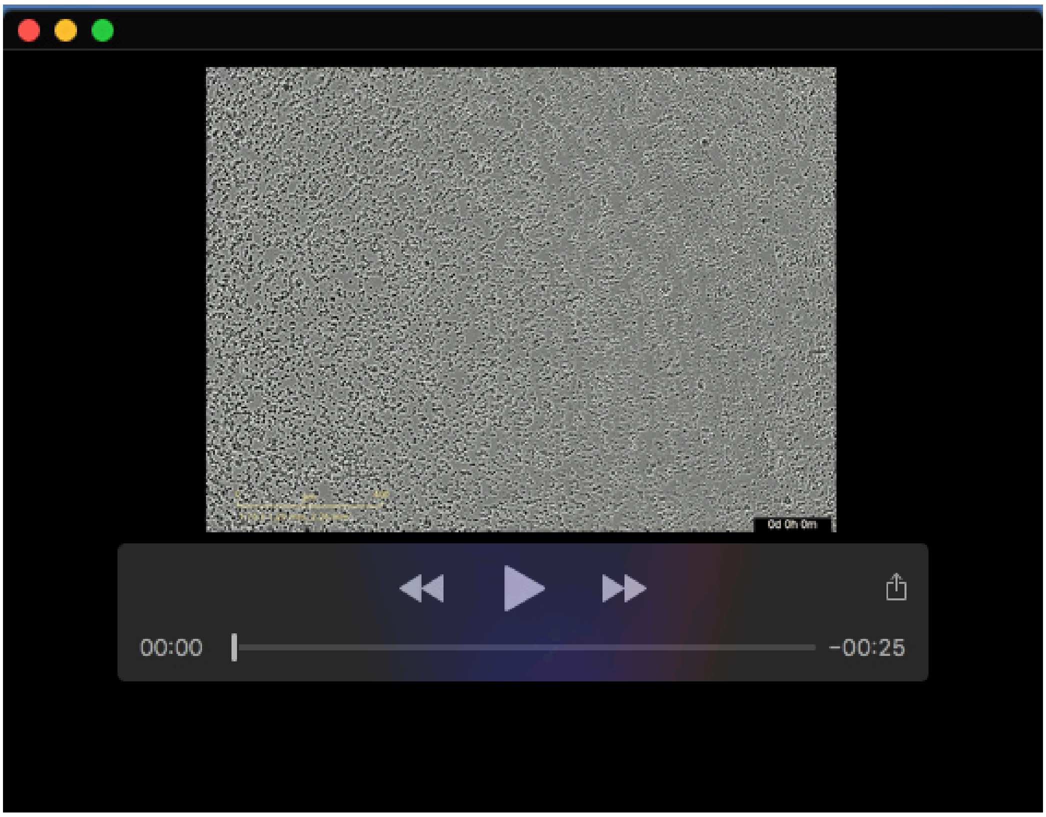

Movie 1. Time-lapse imaging of untreated mouse hindbrain cells. Primary culture was generated from E10.5 mouse hindbrains, cells were seeded (n=6 wells for each treatment) and recorded every 3 hours for 5 days. Video is showing a time-lapse imaging of a representative well. Time is indicated at the bottom right corner of the video.

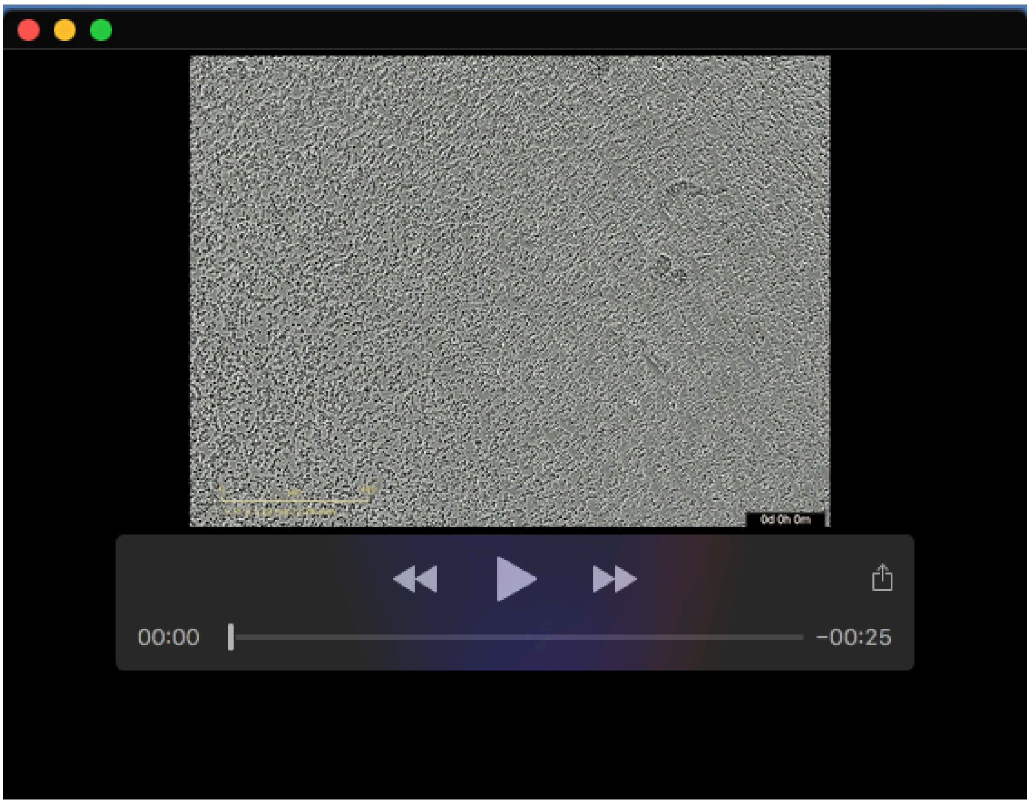

Movie 2. Time-lapse imaging of ChABC-treated mouse hindbrain cells. Primary culture was generated from E10.5 mouse hindbrains, cells were seeded (n=6 wells for each treatment) and recorded every 3 hours for 5 days. At time 0 and then every 48h, cells were exposed to 50 mU/ml of ChABC. Video is showing a time-lapse imaging of a representative well. Time is indicated at the bottom right corner of the video.

Development • Supplementary information

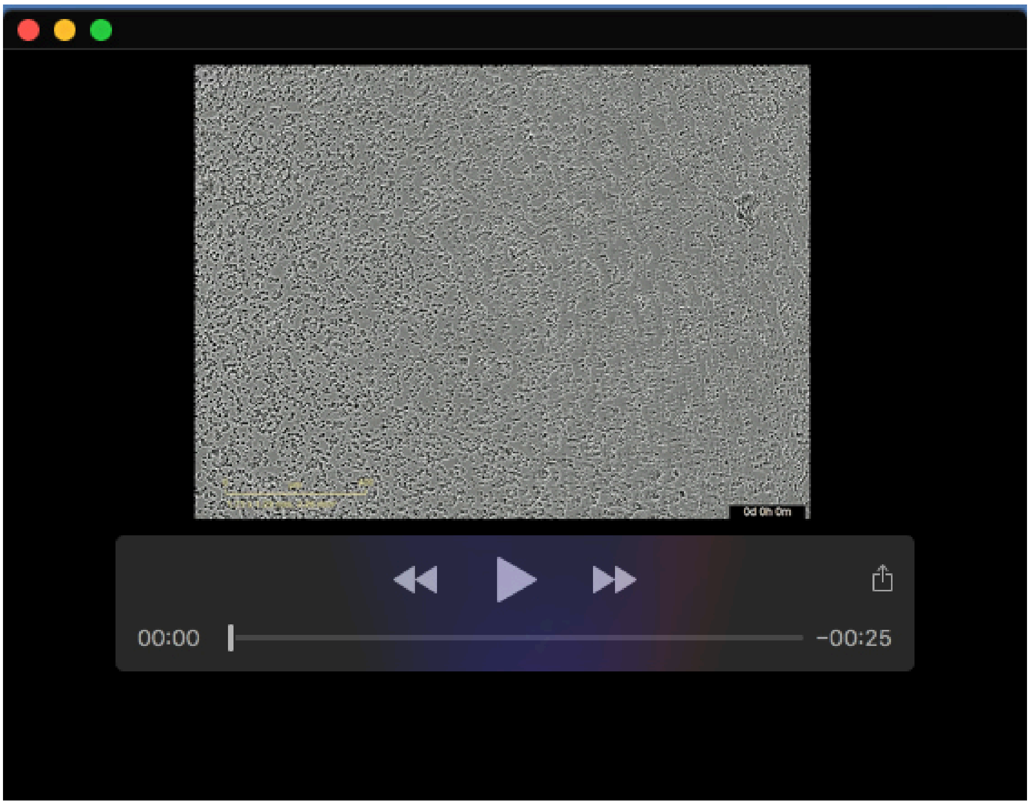

Movie 3. Time-lapse imaging of mouse hindbrain cells exposed to excess CSPG. Primary culture was generated from E10.5 mouse hindbrains, cells were seeded (n=6 wells for each treatment) and recorded every 3 hours for 5 days. At time 0 and then every 48h, cells were exposed to 50 mg/ml proteoglycan from bovine nasal septum. Video is showing a time-lapse imaging of a representative well. Time is indicated at the bottom right corner of the video.

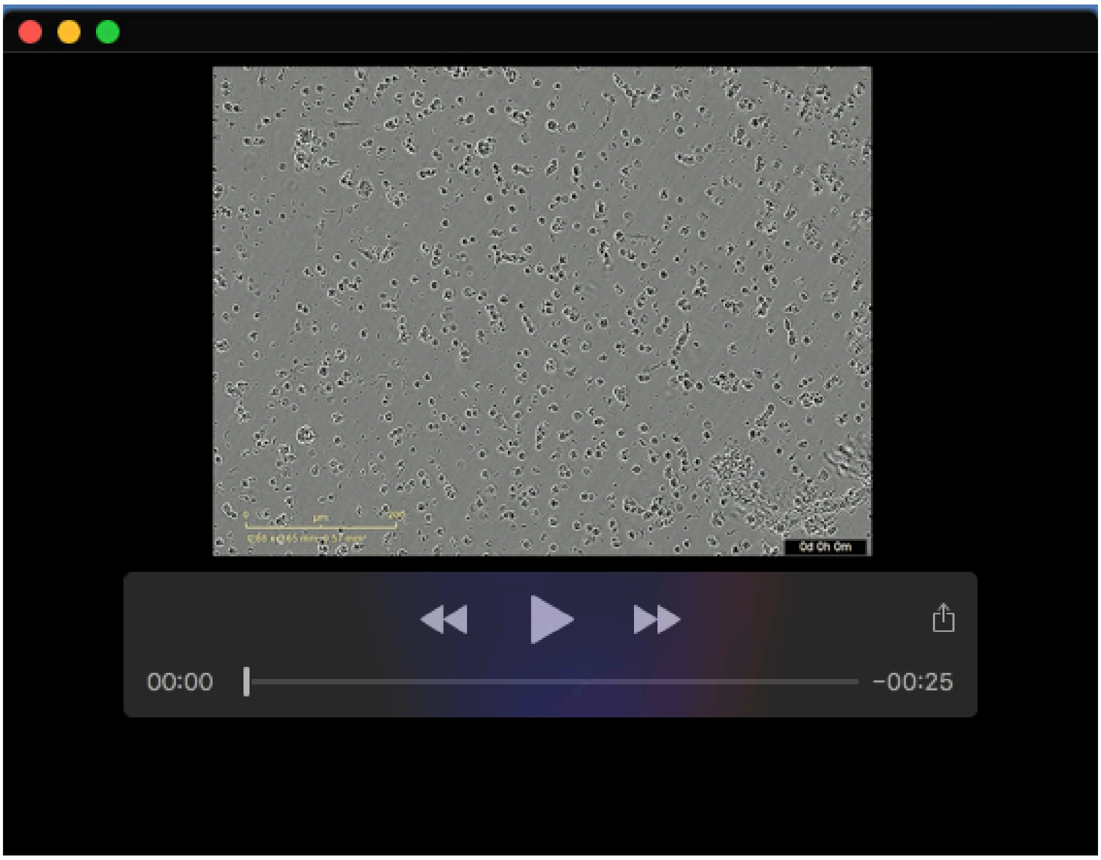

Movie 4. Time-lapse imaging of untreated chick hindbrain cells. Primary culture was generated from st.18 chick hindbrains, cells were seeded (n=8 wells for each treatment) and recorded every 6 hours for 88 hours. Time is indicated at the bottom right corner of the video.

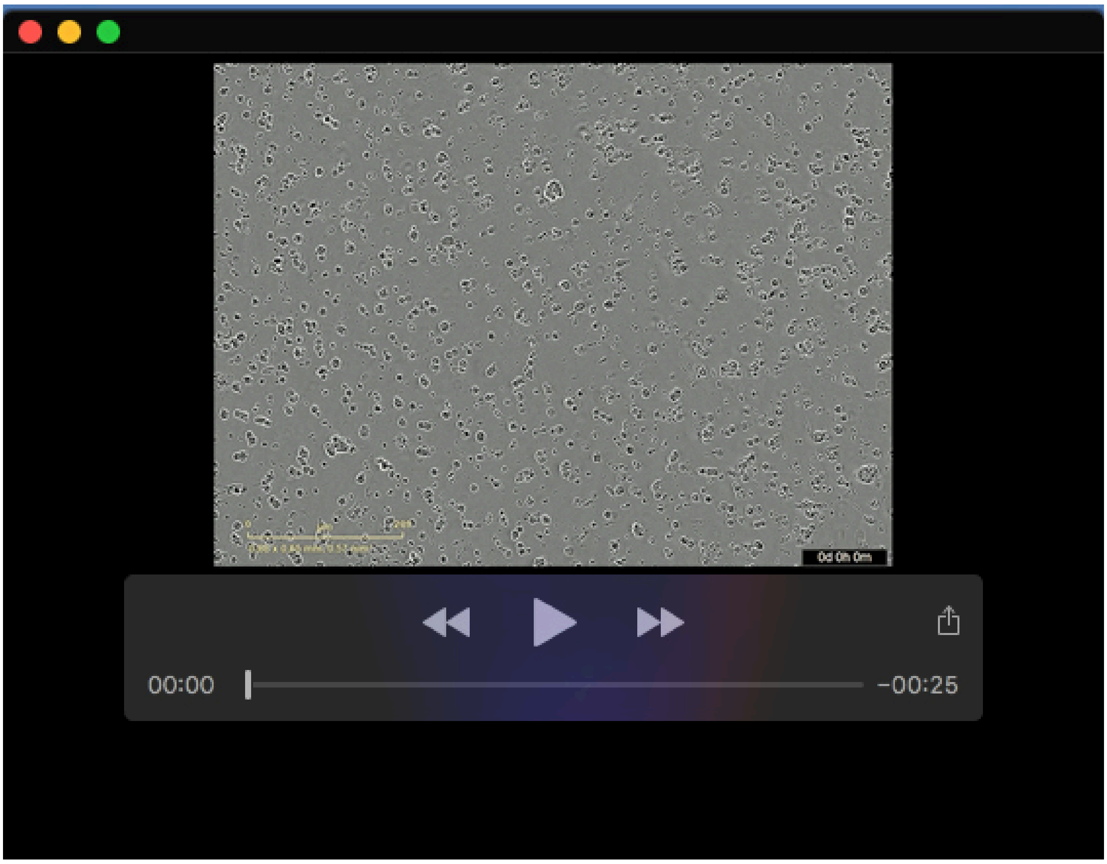

Movie 5. Time-lapse imaging of ChABC-treated chick hindbrain cells. Primary culture was generated from st. 18 chick hindbrains, cells were seeded (n=8 wells for each treatment) and recorded every 6 hours for 88 hours. At time 0 and then every 48h, cells were exposed to 50 mU/ml of ChABC. Video is showing a time-lapse imaging of a representative well. Time is indicated at the bottom right corner of the video.

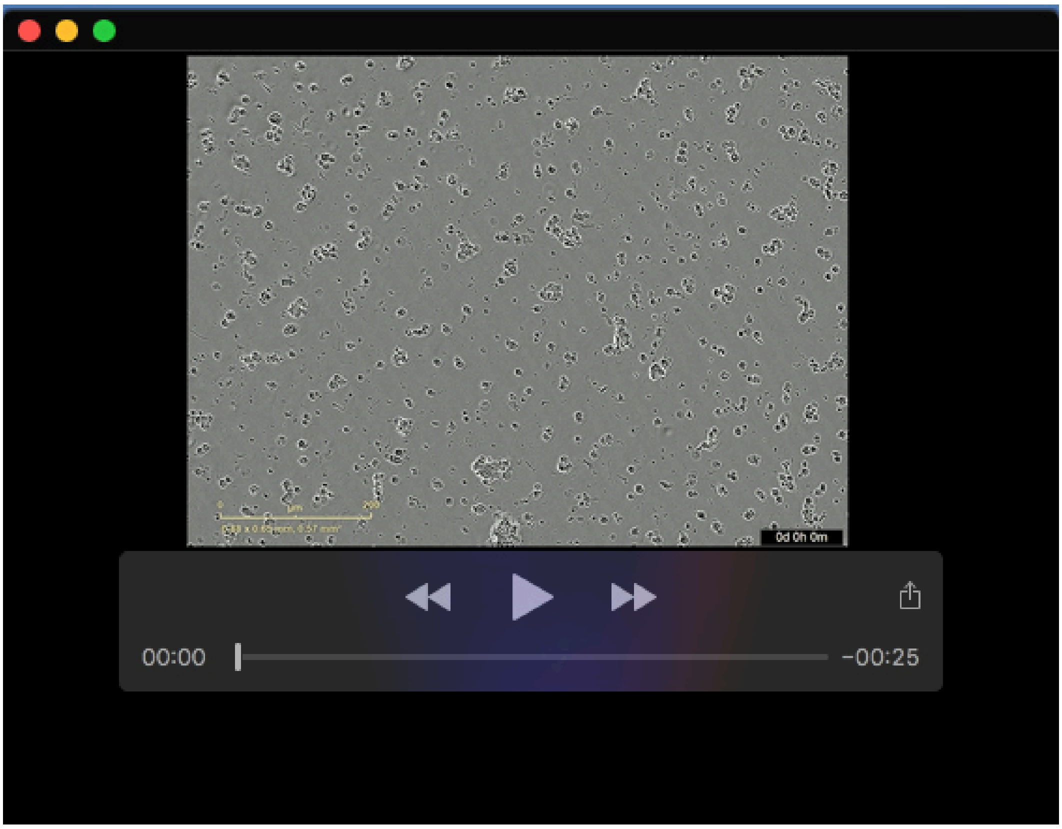

Movie 6. Time-lapse imaging of chick hindbrain cells exposed to excess CSPG. Primary culture was generated from st. 18 chick hindbrains, cells were seeded (n=8 wells for each treatment) and recorded every 6 hours for 88 hours. At time 0 and then every 48h, cells were exposed to 50 mg/ml proteoglycan from bovine nasal septum. Video is showing a time-lapse imaging of a representative well. Time is indicated at the bottom right corner of the video.

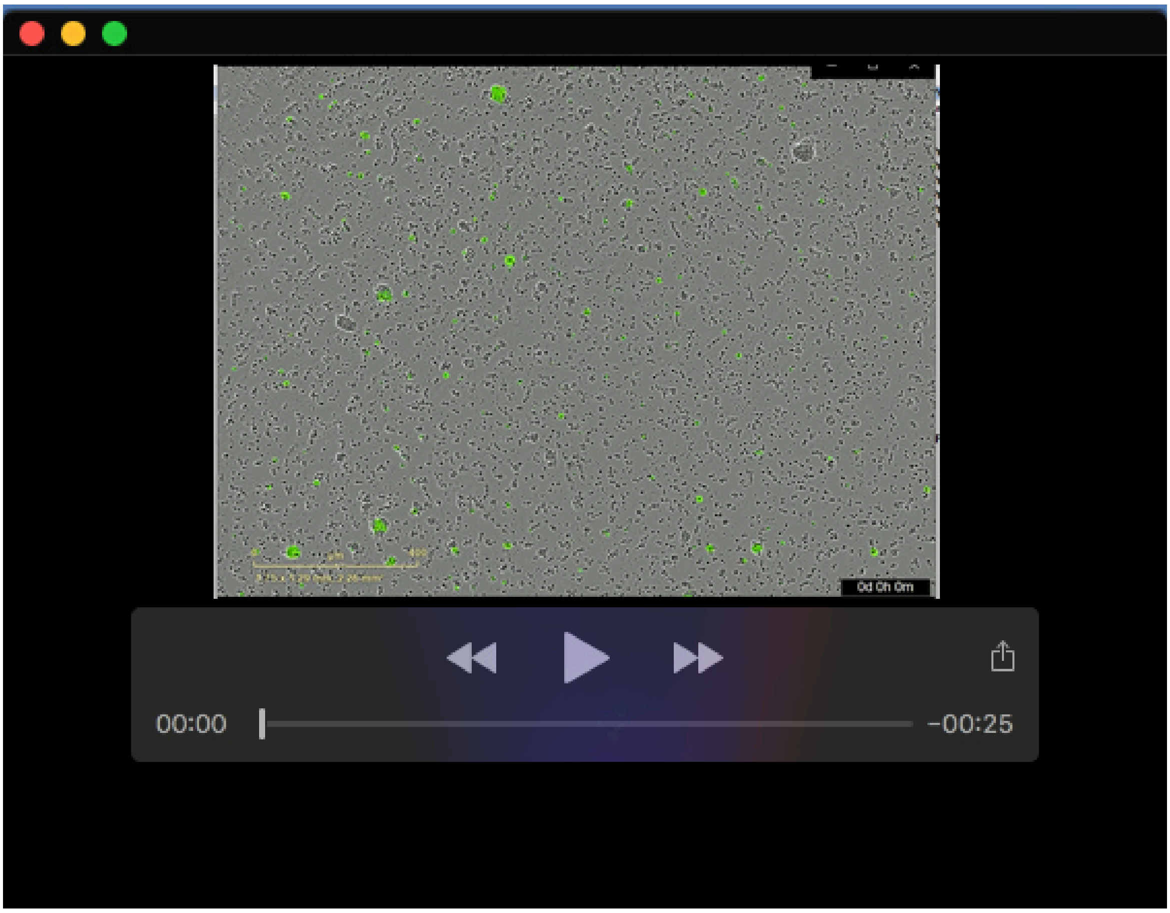

Movie 7. Time-lapse imaging of chick hindbrain cells electroporated with pcDNA3.1-GFP plasmid showing phase/GFP merge image. Primary culture was generated from st.18 chick hindbrains following electroporation and ON incubation, cells were seeded (n=12 wells for each treatment) and recorded every 6 hours for 5 days. Time is indicated at the bottom right corner of the video.

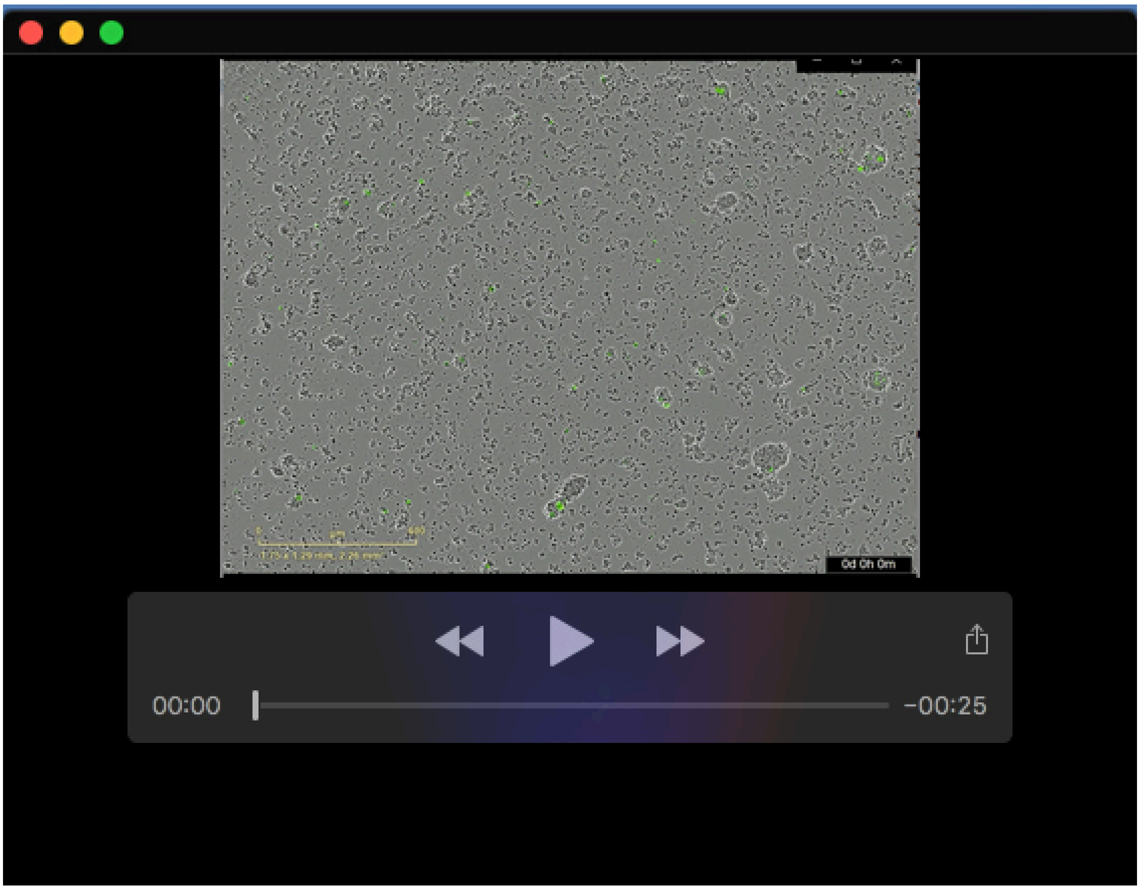

Movie 8. Time-lapse imaging of chick hindbrain cells electroporated with pcDNA3.1-ChABC plasmid showing phase/GFP merge image. Primary culture was generated from st.18 chick hindbrains following electroporation and ON incubation, cells were seeded (n=12 wells for each treatment) and recorded every 6 hours for 5 days. Time is indicated at the bottom right corner of the video.

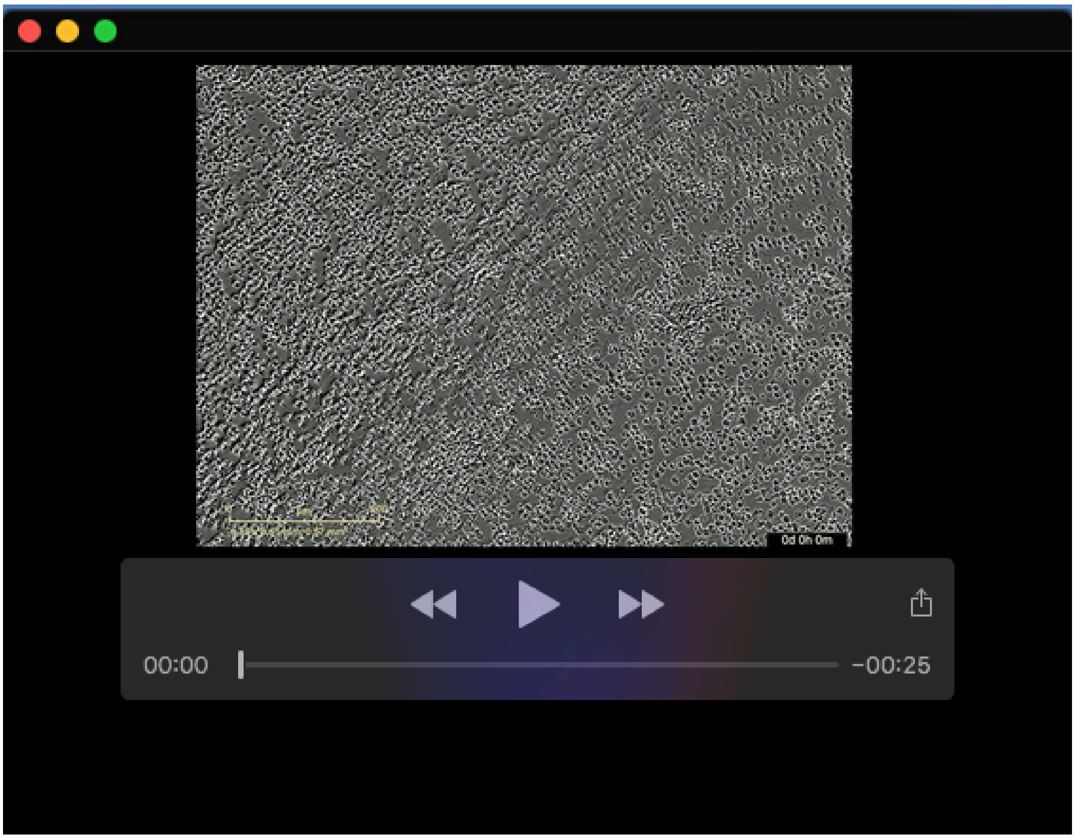

Movie 9. Time-lapse imaging of separated CSPG+ hindbrain cells. Hindbrains of st.18 chick embryos were separated to CSPG+\CSPG- fractions, then seeded separately and recorded every 4 hours for 88 hours (n=4 wells for each group). Video is showing a time-lapse imaging of a representative well. Time is indicated at the bottom right corner of the video.

Development • Supplementary information

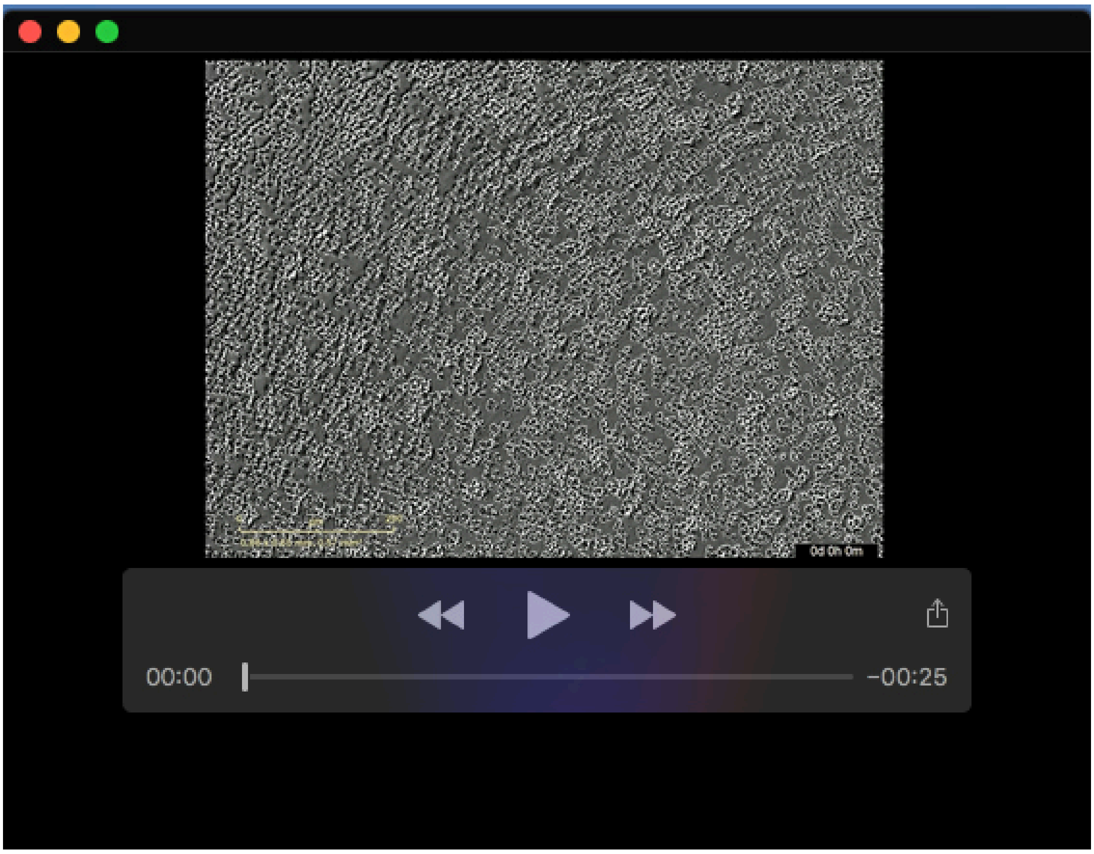

Movie 10. Time-lapse imaging of separated CSPG- hindbrain cells. Hindbrains of st.18 chick embryos were separated to CSPG+\CSPG- fractions, then seeded separately and recorded every 4 hours for 88 hours (n=4 wells for each group). Video is showing a time-lapse imaging of a representative well. Time is indicated at the bottom right corner of the video.

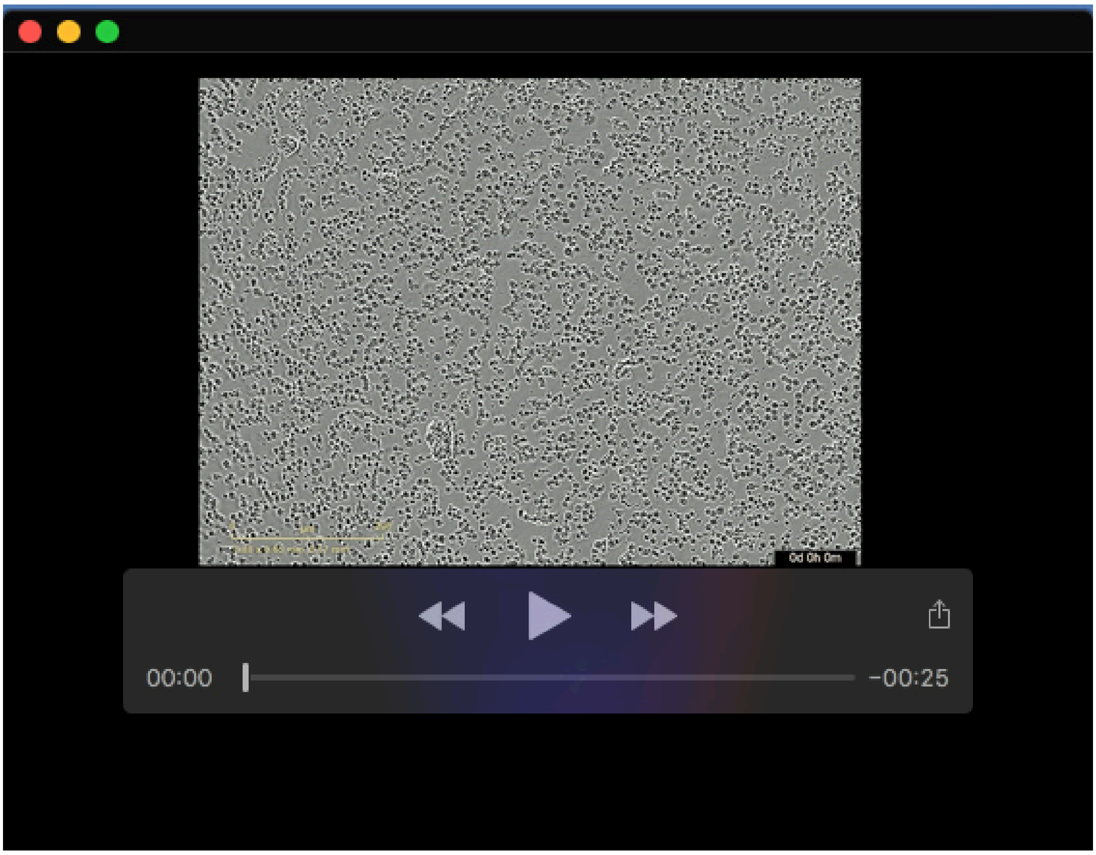

Movie 11. Time-lapse imaging of untreated separated CSPG+ hindbrain cells. Hindbrains of st.18 chick embryos were separated to CSPG+\CSPG- fractions, CSPG+ cells were seeded and recorded every 3 hours for 4 days (n=4 wells for each group). Video is showing a time-lapse imaging of a representative well. Time is indicated at the bottom right corner of the video.

Development • Supplementary information

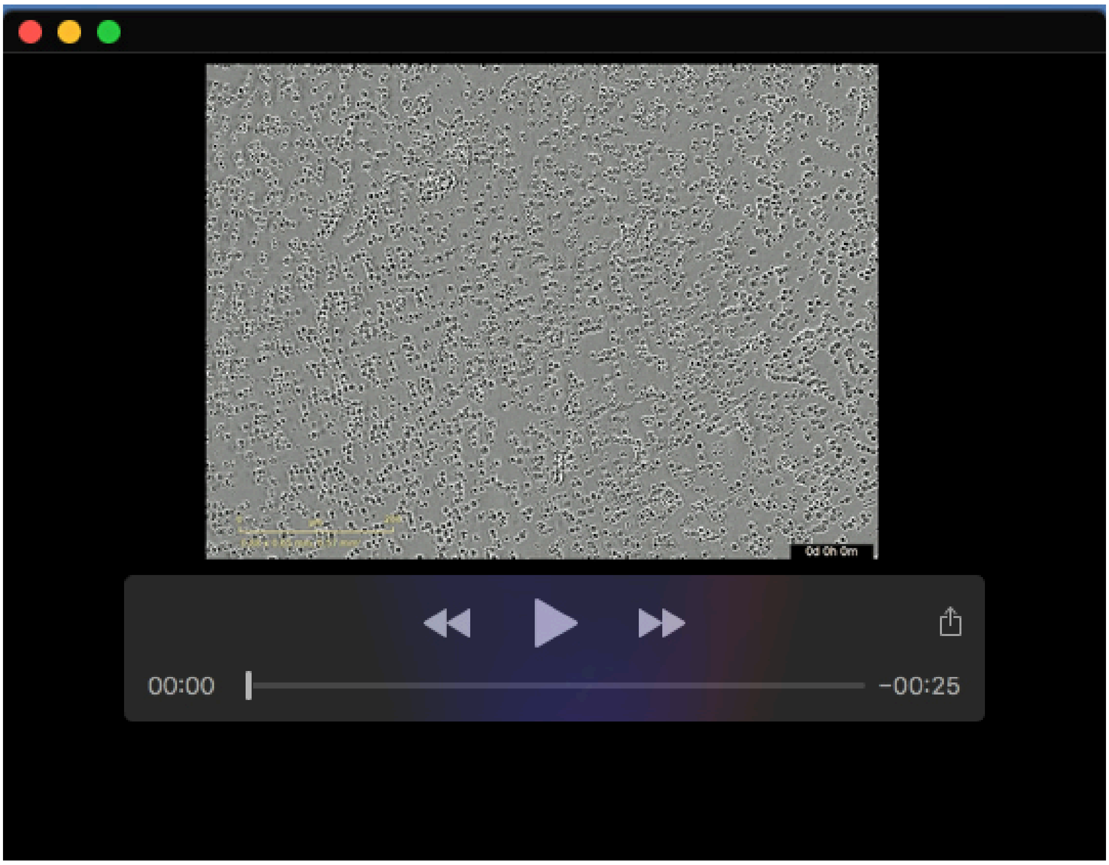

Movie 12. Time-lapse imaging of ChABC-treated separated CSPG+ hindbrain cells. Hindbrains of st.18 chick embryos were separated to CSPG+\CSPG- fractions, CSPG+ cells were seeded and recorded every 3 hours for 4 days (n=4 wells for each group). At time 0 and then every 48h, cells were exposed to 50 mU/ml of ChABC. Video is showing a time-lapse imaging of a representative well. Time is indicated at the bottom right corner of the video.

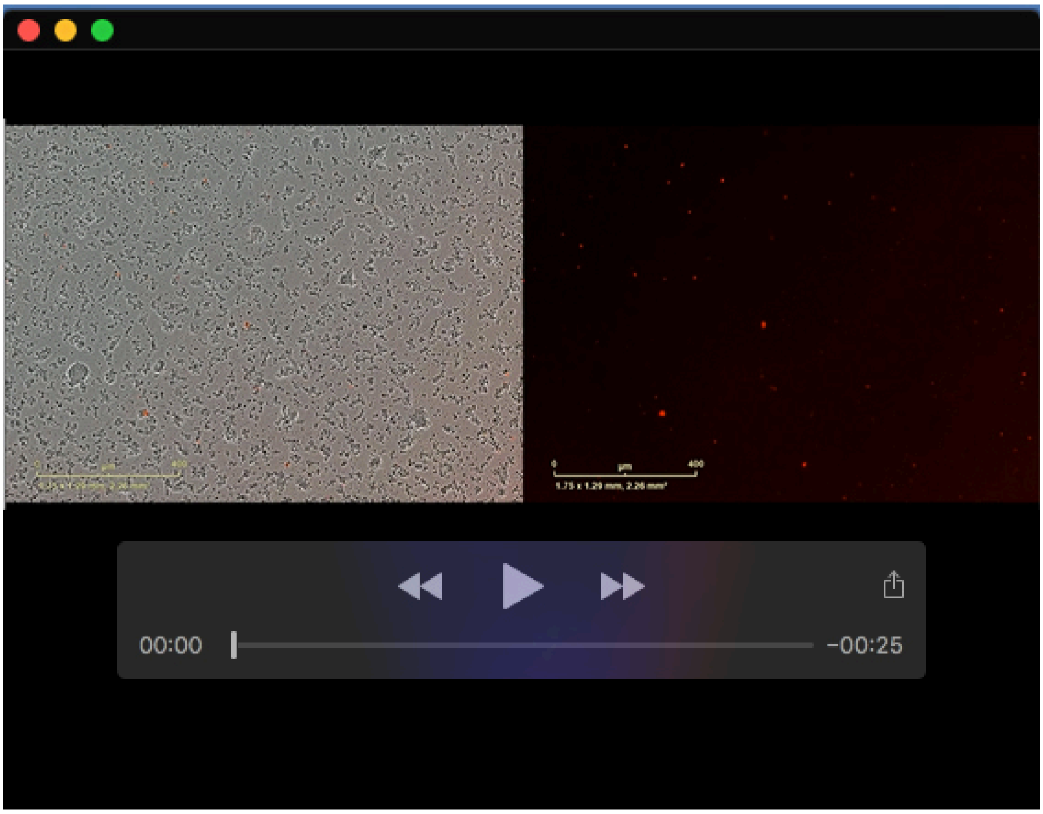

Movie 13. Time-lapse imaging of untreated hindbrain cells with HB cells labeled with Cm-DiI. Hindbrains of st.15 chick embryos were manually labeled with CM-DiI in their HB regions, then taken to generate a primary culture. Cells were seeded and recorded every 6 hours for 6 days (n=12 wells for each group). Video is showing a time-lapse imaging of a representative well. Phase/red merge view is presented to the left, red view alone is presented on the right, showing just labeled cells.

Development • Supplementary information

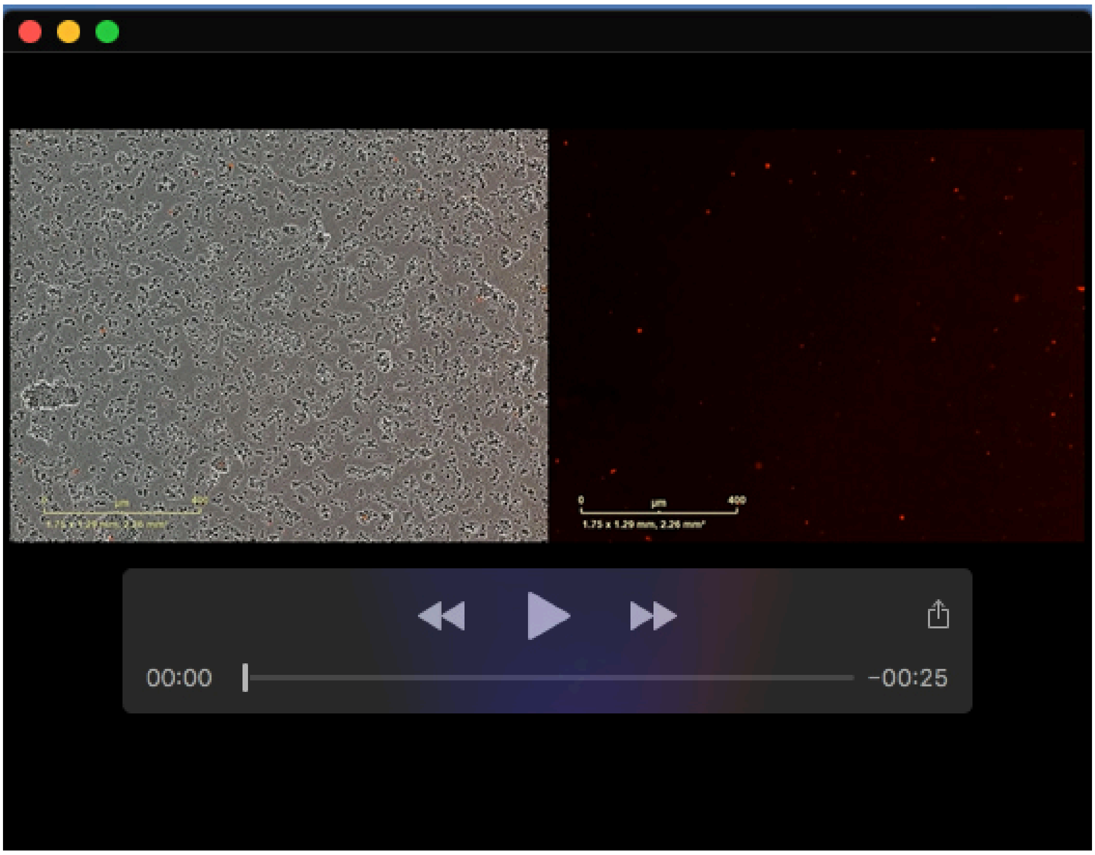

Movie 14. Time-lapse imaging of ChABC-treated hindbrain cells with HB cells labeled with Cm-DiI. Hindbrains of st.15 chick embryos were manually labeled with CM-DiI in their HB regions, then taken to generate a primary culture. Cells were seeded and recorded every 6 hours for 6 days (n=12 wells for each group). At time 0 and then every 48h, cells were exposed to 50 mU/ml of ChABC. Video is showing a time-lapse imaging of a representative well. Phase/red merge view is presented to the left, red view alone is presented on the right, showing just labeled cells.
