# Supplementary material for: Hindbrain boundaries as niches of neural progenitor and stem cells regulated by the extracellular matrix proteoglycan chondroitin sulphate
Source: Development. 2024 Feb 13;151(4):dev201934. doi: 10.1242/dev.201934 (PMC10911165; doi:10.1242/dev.201934)
Supplement: Supplementary file 1 [file develop-151-201934_review_history.pdf]

Hindbrain boundaries as niches of neural progenitor and stem cells regulated by the extracellular matrix proteoglycan chondroitin sulphate.

Carmel Hutchings, Yarden Nuriel, Daniel Lazar, Ayelet Kohl, Elizabeth Muir, Olga Genin, Yuval Cinnamon, Hadar Benyamini, Yuval Nevo and Dalit Sela-Donenfeld
DOI: 10.1242/dev.201934

Editor: James Briscoe

Review timeline
Original submission: 2 May 2023
Editorial decision: 21 June 2023
First revision received: 10 December 2023
Accepted: 12 January 2024

Original submission

First decision letter

MS ID#: DEVELOP/2023/201934

MS TITLE: Hindbrain boundaries as niches of neural progenitor/stem cells regulated by the extracellular matrix proteoglycan chondroitin sulphate.

AUTHORS: Carmel Hutchings, Yarden Nuriel, Daniel Lazar, Ayelet Kohl, Elizabeth Muir, Yuval Nevo, Hadar Benyamini, and Dalit Sela-Donenfeld

I have now received all the referees' reports on the above manuscript, and have reached a decision. The referees' comments are appended below, or you can access them online: please go to BenchPress and click on the 'Manuscripts with Decisions' queue in the Author Area.

As you will see, the referees express considerable interest in your work, but have some significant criticisms and recommend a substantial revision of your manuscript before we can consider publication. Several clarifications are requested. In addition, the referees highlight issues that need to be addressed to strengthen the link between the experiments you report and the conclusion of the study that is indicated in the title. How the observation of co-expression of Sox2 and CSPG outside of the boundaries and the enrichment of TuJ1 in boundary regions is consistent with the conclusion needs to be addressed. Further characterisation of the ChABC experiments is also needed to address questions raised by the Referees 1 and 3.

If you are able to revise the manuscript along the lines suggested, which may involve further experiments, I will be happy receive a revised version of the manuscript. Your revised paper will be re-reviewed by one or more of the original referees, and acceptance of your manuscript will depend on your addressing satisfactorily the reviewers' major concerns. Please also note that Development will normally permit only one round of major revision. If it would be helpful, you are welcome to contact us to discuss your revision in greater detail. Please send us a point-by-point response indicating your plans for addressing the referee’s comments, and we will look over this and provide further guidance.

Please attend to all of the reviewers' comments and ensure that you clearly highlight all changes made in the revised manuscript. Please avoid using 'Tracked changes' in Word files as these are lost

© 2024. Published by The Company of Biologists under the terms of the Creative Commons Attribution License (https://creativecommons.org/licenses/by/4.0/).

in PDF conversion. I should be grateful if you would also provide a point-by-point response detailing how you have dealt with the points raised by the reviewers in the 'Response to Reviewers' box. If you do not agree with any of their criticisms or suggestions please explain clearly why this is so.

Reviewer 1

Advance summary and potential significance to field

The article from Hutchings et al explores further how Sox2-expressing boundary cells behave as neural progenitors. Previously, the same lab had described that hindbrain boundary cells in the chick embryo express Sox2 and the proteoglycan CSPG. They showed that the majority of the Sox2-cells within the boundary core slow-divided, and they could form neurospheres in culture (Peretz et al, 2016).
Here, they show that Sox2-boundary cells do display CSPG, both in chick and mice embryos and several functional experiments suggest that CSPG maintains Sox2 boundary cells in the progenitor state. Transcriptomic analyses of CSPG-sorted boundary cells reveal they express neural progenitor genes. The authors state that CSPG maintains boundary cells in the undifferentiated state. The manuscript brings important findings such as the transcriptomic analyses of boundary cells.

Comments for the author

1. There is a main point all along the manuscript: Sox2 and CSPG are co-expressed in the most dorsal part (lateral in the flat-mounts) all along the hindbrain, not being boundary specific. Authors do not discuss this in the manuscript, but it is a point to be considered specially because they measure CSPG fluorescence and sort these CSPG-cells for later neurosphere experiments and transcriptional analyses.
2. As a general question, how is the timing of CSPG enrichment? Does it play an early role in rhombomere cells entering earlier in neurogenesis? Is this role specific of boundaries? If so, how cells distinguish the levels of CSPG and Sox2 to know how to behave or what to become?
3. About areas quantification: how can CSPG and Sox2 cell areas be compared between boundaries and rhombomeres, considering that CSPG is a membrane protein and Sox2 a nuclear one? in the same line, in mice (Fig 1N) the Sox2 nuclear area is higher than the CSPG area, how can this be explained? did the authors perform the controls of fluorescence intensities and possible differences due to the gain used in the image acquisitions?
4. TUJ1 staining is enriched in the boundaries, where Sox2 cells are. How do they explain so? Could they provide co-staining Sox2/TUJ1? Are these neurons originated from neighboring regions, can they trace them? When they treat embryos with ChABC, it seems they have non-cell autonomous effects because they observed a decrease of Sox2 in the whole hindbrain, and get ectopic TUJ1 cells even in the rhombomeres where there is no CSPG. They could respond to both questions by electroporation of GFP/ChABC-GFP and trace the cells, the fate and the cell-autonomous vs non-cell autonomous effect. Can they provide double staining progenitor/neuron upon ChABC treatment and use neural-committed cell staining such as proneural genes, instead of TUJ1 (a late differentiation gene)?
5. Do Sox2 boundary cells proliferate less upon ChABC? Or they switch fate?
Authors can do EdU and TUNEL experiments to rule out these possibilities. In the same line, culture experiments in Figure 5 seem to point out to the fact that there are no changes in the number of cells in different conditions. Isn't it contradictory with the hypothesis of CSPG maintaining boundaries as progenitors and preventing to undergo differentiation? Again, point 1, are authors sure that they have only CSPG-boundary cells and not the rest of CSPG-cells along the dorsal hindbrain? Does CSPG maintain boundary cells as slow dividing, or as dividing progenitors?
6. Can they provide a better characterization of the neurospheres? Number of progenitors (Sox2) vs. number of neuronal-committed cells (proneural) and differentiated cells (elav, TUJ1, 3A10). It surprises me to have Sox2 cells expressing TUJ1.
7. Could they verify their transcriptomic findings with in situ hybridization stainings? Not all the genes, but a subset of the most interesting ones to check that indeed they are enriched or are specific of the hindbrain boundaries, and not only in the most dorsal domain along AP axis.

Reviewer 2

Advance summary and potential significance to field

© 2024. Published by The Company of Biologists under the terms of the Creative Commons Attribution License (https://creativecommons.org/licenses/by/4.0/).

This is a comprehensive study that links classical findings on the nature of rhombomere boundaries in the developing vertebrate hindbrain with concepts of the role of the extracellular matrix (ECM) in stem cell niche maintenance. The authors have performed in vivo and in vitro experiments in amniote embryos (chick, mouse) as well as in primary cell culture to show that chondroitin sulfate proteoglycans (CSPGs) are expressed at high levels in rhombomere boundaries (as described by Heyman et al. in 1995) and that this ECM component is both necessary and sufficient to maintain a proliferative neural progenitor-like state in neuroepithelial cells whereas removal of CSPG leads to neuronal differentiation. The authors have also used gene expression profiling to compare the transcriptome of CSPG+ boundary cells and CSPG- non-boundary cells, and these experiments have led to the identification of batteries of genes expressed in either of those cell populations, including several genes that have been previously described (confirming the validity of the approach) but also a number of novel factors involved in neuronal proliferation or differentiation and in tumorigenesis.

As the authors discuss in the final section of the paper, their results not only provide a comprehensive transcriptomic description of a classical neurodevelopmental paradigm, they also highlight important links between early neural development and the potential emergence of brain tumours such as the highly aggressive medulloblastomas that often develop during childhood.

Comments for the author

The data presented in this study are comprehensive and of excellent quality. It would be nice to see an attempt to translate the CSPG gain of function experiments into embryos - is it possible to over-express or ectopically apply CSPGs in chick embryos and test how that influences the boundary cell type?

It also appears that a legend for Fig. 3 is entirely missing. In panel G of this figure, Tubb3 is analysed but this gene is not mentioned in the main text.

On page 9, it is mentioned that 'genes involved in neurogenesis, axonogenesis and neuroectoderm induction [...] were notably downregulated in the CSPG+ group'.
Neuroectoderm induction is a very early step in neural development that leads to the generation of proliferating neural progenitor cells which seems at odds with the notion that CSPGs maintain a stem cell-like state?

<u>Reviewer 3</u>

Advance summary and potential significance to field

The paper by Hutchings et al. analyses the function of CSPG in the maintenance of hindbrain boundaries (HBs) in the chick and mouse developing neural tube. The authors describe an enrichment in CSPG at the surface of Sox2+ cells in HBs.
They demonstrate that reduction of CSPG in vivo (upon treatment with the chodroitinase ChABC, or forced expression of ChABC with electroporation) leads to the loss of Sox2 expression and increased neuronal differentiation. They also make use of CSPG to sort CSPG+ and CSPG- cells from embryonic hindbrains characterize the respective bulk transcriptomes, and monitor a different, CSPG-dependent behavior in culture: while CSPG+ cells tend to form Sox2-positive spheres with little differentiation, CSPG- cells display the opposite phenotype.
The authors conclude on a role for CSPG in maintaining their expressing cells as neural progenitors at HBs.

There are several potentially interesting aspects to this paper, notably the evolutionary considerations, and the functional perturbations of CSPG that add to numerous previous studies in other systems highlighting the importance of this ECM component on cell state and fate.

However, I feel that the paper falls short of bringing us much forward. This is in a large part since analyses are conducted at the population level, in systems (whether in vivo or in vitro) that are a heterogeneous mixture of cells.

© 2024. Published by The Company of Biologists under the terms of the Creative Commons Attribution License (https://creativecommons.org/licenses/by/4.0/).

Individual cell fates are not analyzed, and the conclusions that follow are logical but not thoroughly demonstrated. For example, upon CSPG decrease, Sox2 expression decreases and TuJ1 expression and neurites increase, but without tracing actually showing the differentiation of previously Sox2+ cells the two observations could correspond to two different effects. Under this light, I see the progress brought by this paper as relatively minor.

Comments for the author

Current major limitations:
1. In vivo manipulations (Fig.3):
a. The effect of CSPG decrease on Tuj1 is puzzling: first, Tuj1 seems to be enriched at HBs under physiological conditions (Fig.3E, top) while HBs should be largely composed of progenitor cells. Second, Tuj1 induction upon ChABC seems to occur primarily inside compartments as opposed to HBs (Fig.3E, bottom). Can the authors explain these surprising observations?
b. HB cells would need to be traced to monitor their progenitor/differentiation status at the individual cell level upon ChABC treatment. Do individual Sox2+ cells convert to differentiation and TuJ1 expression? Can the authors rule out cell migration? Is the fate of individual compartment cells unchanged?
2. In vitro manipulations (Figs.8): given that the cultures are not pure (4 times enrichment for CSPG+ cells only), Sox2+ cells need to be individually traced to monitor their fate and ensure that excess Tuj1 upon ChABC is indeed due to the differentiation of previously Sox2+ cells (as opposed to independent increase in neuritogenesis, for example).

First revision

Author response to reviewers' comments

Dear Dr. Briscoe, Editor,

We are happy to re-submit our revised paper "Hindbrain boundaries as niches of neural progenitor/stem cells regulated by the extracellular matrix proteoglycan chondroitin sulphate (DEVELOP/2023/201934). We would like to thank you and the reviewers for the thorough reading of the manuscript and the insightful comments and suggestions. We have fully addressed all the requests and questions raised by the reviewers, which led us to design multiple new experiments as well as add new information and clarification/edition to the text. Consequently, the revised manuscript includes 3 additional new figures (new Figs. 4,6,10), 7 additional supplementary figure (new Figs. S2, S3, S5, S6, S8, S10, S13) and 4 additional movies (new movies 7,8,13,14), as well as contains multiple additions to existing figures (such as in Figs. 2F,H, 9C, S1B, S12). We believe that we substantially upgraded the merit and significance of the study and sharpened its message. We hope that you will find our revised manuscript suitable for publication in 'Development.

We apologize for the length of the reply leter, which was unavoidable due to the number or points addressed. Moreover, the many requests led inevitably to a substantial increase in the article length, which now contains 10 main figures, 13 supplementary figures, and 14 movies. Hence, we had to exceed the word limit (now ~8700 words), and hope this will be acceptable.

Our point-by-point response to the reviewer's comments is added below.

Reviewer 1-

1. There is a main point all along the manuscript: Sox2 and CSPG are co-expressed in the most dorsal part (lateral in the flat-mounts) all along the hindbrain, not being boundary specific. Authors do not discuss this in the manuscript, but it is a point to be considered specially because they measure CSPG fluorescence and sort these CSPG-cells for later neurosphere experiments and transcriptional analyses.

© 2024. Published by The Company of Biologists under the terms of the Creative Commons Attribution License (https://creativecommons.org/licenses/by/4.0/).

1. Reply: We thank the reviewer for this important comment. Indeed, it is interesting that, in addition to the rhombomere boundaries, the dorsal-most part of the rhombomeres also seem to expresses Sox2 and CSPG. We have addressed this concern thoroughly and provide evidences that support that our study deals particularly with the boundary specific Sox2+/CSPG+ cell population, as detailed bellow:

1.1. CSPGs are a family of proteins, most of which are secreted, and only a few are membrane-bound. The canonical CSPG antibody that we have used (CS-56) marks the sulfated GAG-chains in all of them. Yet, we have reasoned that the CSPG subtype in the boundaries is a membrane-bound one, based on the immunostaining patern, as well as on the ability to sort the cells (as otherwise cells could not be sorted when based on an extracellular protein). The main membrane-bound CSPG is DSD1 (also termed PTPRz1), to which an antibody exist. To verify the possibility that the boundary-CSPG is indeed a membranal one, we have used the DSD1 antibody, and found it to be exclusively confined to the boundaries, with no dorsal expression (New Fig.S1). This corroborates the data reported in *Heyamn et al., 1995,* where another DSD1 antibody had been used (473HD). Notably, the observed DSD1 expression patern is in agreement with the RNAseq data which found upregulation of PTPRz1 in the CSPG^{+} cell group (Fig.5, new Fig.6), whereas several secreted CSPGs (NCAN, VCAN, CSPG5) were actually enriched in the CSPG- cell group. These results indicate that the boundary cells express a different type of CSPG than the dorsal rhombomeres, evidently a membranal-one (now discusses in page 5).

1.2. if indeed the dorsal CSPG+ hindbrain cells were sorted-out together with the boundary cells, that would predict that typical dorsally-expressed genes would be enriched in the CSPG+ cell group in the RNAseq. Yet, by analyzing the data, many dorsal hindbrain markers (i.e., Atho1, Lhx2&9, Lmx1b, Msx1&2, Olig3, Wnt3a) were found to upregulated in the CSPG- cell group, ruling out the possibility that the dorsal rhombomere cells were collected together with the boundary cells. This new data is now presented in the new Fig.6 and in page 10.

We believe these evidences strongly support the point that the CSPG+/Sox2+ cells investigated in this research are the boundary-localized ones. Investigating the dorsal-subgroup, which is also very interesting, will be the focus of a separated study.

2. As a general question, how is the timing of CSPG enrichment? Does it play an early role in rhombomere cells entering earlier in neurogenesis? Is this role specific of boundaries? If so, how cells distinguish the levels of CSPG and Sox2 to know how to behave or what to become?

2. Reply: We have addressed this point by analyzing the expression of Sox2 and CSPG at various HH stages (14,16,18,20). The data shows that the co-expression of CSPG and Sox2 is more profound at the boundaries at stages 16-20, rather than at st.14. In the earlier stage, Sox2 is more broadly expressed at rhombomere cells (as also reported in *Peretz et at, 2016*), with very weak/no CSPG expression (although it begins to appear as a stripe at the boundaries). These results suggest that Sox2/CSPG co-expression is evidently prominent at the boundary cells of older embryos rather than at rhombomeres, where Sox2 is expressed at earlier stages, indicating that CSPG does not play a major role at earlier stages (as also suggested for CSPG in Heyman et al., 1995) and therefore a main role of CSPG on rhombomere cells is less probable. The spatial characterization is now presented in the new Fig. S2 and discussed in page 5.

3. About areas quantification: how can CSPG and Sox2 cell areas be compared between boundaries and rhombomeres, considering that CSPG is a membrane protein and Sox2 a nuclear one? in the same line, in mice (Fig 1N) the Sox2 nuclear area is higher than the CSPG area, how can this be explained? did the authors perform the controls of fluorescence intensities and possible differences due to the gain used in the image acquisitions?

3. Reply: Quantification was done by comparison of the mean fluorescence of individual markers in manually-selected areas along with subtracting the background for each measurement and normalizing the area sizes, using ImageJ software. As such, comparison of fluorescent area in Rhs vs. HB In Fig.1G, was done separately for CSPG and Sox2, as we did not compare between the two markers but quantified the relative expression for each channel in Rh vs. HB regions. Hence, the different staining paterns (membranal\nuclear) are not affecting the results. Moreover, in mice, for

© 2024. Published by The Company of Biologists under the terms of the Creative Commons Attribution License (https://creativecommons.org/licenses/by/4.0/).

obvious limitations, staining and imaging was done on embryos from different liters obtained on separated occasions (as can also be observed by the higher variability in Fig.1G comparing to Fig.1N). Therefore, different settings were applied for imaging different embryos, which could explain why in some embryos the Sox2+ relative area was higher than the corresponding CSPG area. Different settings were also applied for the different channels, which could also lead to a relatively higher No. of pixels where Sox2 is detected but CSPG is not. Yet, since comparison was done between the fluorescent area of each marker in the different regions of individual embryos, rather than between Sox2 and CSPG, the coincidental higher area of Sox2 in some embryos does not affect our results and claim of higher presence of both markers in the HBs compared to Rhs. Notably, the only co-quantification of Sox2 and CSPG staining was performed by the IMARIS analysis (Fig.O,P), where the adjacent/distant expression of the two markers was measured. Finally, in relation to comment #1, all quantifications of CSPG\Sox2 staining did not include the dorsal parts. These clarifications were now added to the Materials and Methods (page 27).

4. TUJ1 staining is enriched in the boundaries, where Sox2 cells are. How do they explain so? Could they provide co-staining Sox2/TUJ1? Are these neurons originated from neighboring regions, can they trace them?

4. Reply: We thank the reviewer for this important comment (also raised by reviewer 3, point 1a). Actually, the spatial expression of Sox2 and Tuj1 in the HBs was thoroughly addressed in our previous paper (Peretz et al., 2016); While in flat-mounted overviews they seem to co-localize at the HBs, when analyzing the Z axis, Sox2+ cells are shown to localize at the ventricular/sub-ventricular zone, whereas Tuj1+ cells/axons are mostly enriched in in the mantle layer (the enrichment of the boundary's mantle layer with axonal fibers was actually already reported in *Heyman et al., 1995*). Moreover, we have also GFP-labelled and traced the HB-Sox2 cells in our previous study, and demonstrated that the differentiating Tuj1+ neurons which migrate to the HBs-mantle zone, originate from Sox2+ cells in the HBs ventricular zone. Hence, we have concluded that the HBs' population is comprised of progenitor cells (Sox2+) in the ventricular zone that give rise to differentiating cells, which migrate towards the mantle zone and upregulate Tuj1/3A10/HuC/D (Peretz et al., 2016). Notably, our previous analysis also revealed that some of the radially migrating Sox2+ cells begin to upregulate Tuj1 before downregulating Sox2, whereas others upregulate Tuj1 only after reaching the mantle layer and losing Sox2 expression.

Yet, to beter clarify this point in the present manuscript (, we conducted staining in chick and mouse hindbrains, capturing images of the tissue in whole-mounted views and transverse sections. Our analysis reveals that in both species, Sox2+ cells are present in the ventricular and subventricular layers of the HBs but not in the mantle region, while Tuj1 is predominantly localized in the mantle zone. However, some Sox2+/Tuj1+ cells are also observed more apically, consistent with our previous results and similar findings in the chick spinal cord (Peretz et al., 2016, Das and Storey 2014). These data are now presented in new Fig. S5 and in the text (page 6). Moreover, we have now also traced boundary cells by electroporating them with control-GFP or ChABC-GFP plasmids and showed their gradual radial migration towards the pial surface, together with their downregulation of Sox2, upregulation of Tuj1 and tangential migration in the mantle zone (Fig. 2F, Fig S6, see also reply 5 below). Finally, these expression paterns have been also verified by our RNAseq data, which shows a differential expression of *Sox2* or *Tuj1* and Huc/D in the CSGP+ or CSPG- groups, respectively (Fig. 5, new Fig. 6).

To conclude, these data suggest that the boundary-localized Tuj1+ neurons originate predominantly from the boundary's Sox2+ progenitors. Yet, we cannot rule out the option that some of the Tuj1+ filaments in the boundaries arrive from rhombomeres (although, it is less likely that rhombomere neurons project longitudinal axons towards the boundaries that turn transversely when reaching there, as this was not observed in our previous hindbrain axonal studies; (i.e., Kohl et al., 2012, 2015).

5. When they treat embryos with ChABC, it seems they have non-cell autonomous effects because they observed a decrease of Sox2 in the whole hindbrain, and get ectopic TUJ1 cells even in the rhombomeres where there is no CSPG. They could respond to both questions by electropora�on of GFP/ChABC-GFP and trace the cells, the fate and the cell-autonomous vs non-cell autonomous effect.

© 2024. Published by The Company of Biologists under the terms of the Creative Commons Attribution License (https://creativecommons.org/licenses/by/4.0/).

5. Reply: As suggested by the referee (see also reply 1b to reviewer 3), we have performed electroporations with control-GFP or ChABC-GFP plasmids and live-traced the cells in vitro. ChABC-expressing cells displayed a marked neural-differentiation morphology of cells that left the sphere, downregulated Sox2 and extended Tuj1+ neurites, in contrast to control-GFP cells that remained incorporated within rounded spheres, expressing Sox2 (new Fig. 4, new Fig. S10, Movies 7-8). The effect was non-cell autonomous, as in the ChABC electroporated group, both the GFP-expressing cells and the non-electroporated cells displayed these phenotypes. Such observation is reasonable since the plasmid encodes a secreted enzyme, hence effecting non-electroporated cells when grown in a culture. We have also performed this experiment in vivo, and analyzed the behavior of GFP-expressing cells in HBs sections; the same phenotypes of higher rates of radial migration of GFP+ cells towards the basal side, extension of GFP+ axons in the mantle zone and upregulation of Tuj1/downregulation of Sox2 in the GFP+ cells were observed in the ChABC-expressing HB cells compared to controls, indicating the direct effect of CSPG loss on the fate of electroporated cells (Fig. 2F,H, Fig. S6C,D,).

As to the comment of the enhanced expression of Tuji1 not only in the boundaries but also in the rhombomeres, one way to explain that is based on the fact that, although the boundaries are the main sites of Sox2/CSPG expression, some rhombomere cells also express Sox2 and CSPG (Peretz et al 2016, and see also Fig.1, Fig. S2). Hence, it is possible that the exposure of these scatered Sox2+/CSPG+ cells in rhombomeres to ChABC have led to their differentiation in these sites. Another possibility is based on our previous findings that some amplifying Sox2+ boundary cells are being contributed to the rhombomeres, where they undergo differentiation (Peretz et al, 2016). Therefore, it is possible that CSPG-loss also accelerates this contribution, leading to enhanced differentiation of boundary-derived Sox2 cells in the rhombomeres. This is now being discussed in pages 6 and 18.

6. Can they provide double staining progenitor/neuron upon ChABC treatment and use neural-commited cell staining such as proneural genes, instead of TUJ1 (a late differentiation gene)?

6. reply: As requested by the reviewer, we have now labelled the cultures with the early differentiation marker doublecortin (DCX), and the late differentiation marker Map2, providing further evidence of the shift towards differentiation when CSPG is removed (new Fig. S9). Yet, we wish to correct the reviewer's comment of Tuj1 being a late marker, as in chick and mouse it is actually one of the most-earlier markers that are being expressed at the onset of neural differentiation, as we and others have previously reported (Memberg, 1995, Das and Storey, 2014, Peretz et al., 2016, D), and as shown in the sections in new Fig. S5. Notably, these markers were also found to be upregulated in the CSPG- cell group of the RNAseq.

7. Do Sox2 boundary cells proliferate less upon ChABC? Or they switch fate? Authors can do EdU and TUNEL experiments to rule out these possibilities. In the same line, culture experiments in Figure 5 seem to point out to the fact that there are no changes in the number of cells in different conditions. Isn't it contradictory with the hypothesis of CSPG maintaining boundaries as progenitors and preventing to undergo differentiation? Again, point 1, are authors sure that they have only CSPG-boundary cells and not the rest of CSPG-cells along the dorsal hindbrain? Does CSPG maintain boundary cells as slow dividing, or as dividing progenitors?

7. Reply: We agree that the question of proliferation is very interesting. In a first atempt to characterize the proliferative behavior of the Sox-boundary cells we have conducted pH3 staining in mouse and chick hindbrains and found a much lower number of Ph3+ cells in the boundaries compared to the rhombomeres in both species (appended figure, panel A). Intriguingly, an opposite patern of BrdU staining was found: A short exposure to BrdU (30 minutes) revealed a higher amount of boundary cells that are BrdU+, compared to rhombomere cells (data not shown, see also appended figure panel B, left image). These paterns suggest that majority of the HB/Sox2+ cells are slow-dividing, as reflected by their longer S phase (which results in more cells incorporating BrdU), together with fewer cells in the M phase (marked by Ph3), as opposed to the cells in the rhombomeres. Notably, when the hindbrains were added with ChABC, a significant increase in the number of Ph3+ cells were found at the boundaries, together with a reduction in the number of BRDU+ cells, indicating that more boundary cells entered mitosis and accelerated their cell cycle CSPG was removed (appended figure, B). This result suggests that the presence of

© 2024. Published by The Company of Biologists under the terms of the Creative Commons Attribution License (https://creativecommons.org/licenses/by/4.0/).

CSPG in the boundary cells maintains them as slow-dividing progenitors, whereas in its lost, they shift to an actively-proliferating state, a mandatory step before moving towards differentiation.

These findings open many new questions regarding, for instance, the manner by which CSPG is coupled to the cell cycle, the molecular regulators that halt the cell's entry into mitosis in the normal conditions (actually, very interesting G2/M regulators have been found in the RNAseq to be upregulated in the CSPG+ group), the expected gradual exit from the cell cycle when these amplifying cells shift towards differentiation, and the possibility that CSPG-loss triggers more amplifying boundary progenitors to enter the rhombomeres and to undergo differentiation. All these questions need to be addressed in a subsequent study; therefore, we prefer not to include the proliferation data in the current manuscript. Yet, we added a new paragraph on this topic in the Discussion (page 18).

Moreover, regarding the TUNEL, we have provided the data showing no change in cell death upon treatment with ChABC (Fig. S4A)

NOTE: We have removed unpublished data that had been provided for the referees in confidence.

8. Can they provide a beter characterization of the neurospheres? Number of progenitors (Sox2) vs. number of neuronal-commited cells (proneural) and differentiated cells (elav, TUJ1, 3A10). It surprises me to have Sox2 cells expressing TUJ1.

8. Reply: The molecular properties of hindbrain-derived neurospheres have been thoroughly covered in our previous study (Peretz et al., 2018). Briefly, in that study, we have demonstrated that Sox2+ cells are distributed throughout the neurosphere, constituting approximately 50% of its total cells. Neural differentiation markers such as doublecortin (DCX), Tuj1, and NeuN, were also shown to be expressed within the spheres, primarily located at the periphery or in the neurites extending from the spheres, consistent with the expected patern for differentiating neurons in neurospheres. These paterns recurred at the present study (Fig. 3E, new Fig. 4, new Fig.10E), showing Tuj1 expression in the sphere's periphery and neurites, with Sox2+ cells scatered throughout the sphere. Concerning Sox2/Tuj1 co-expression, in our previous papers (Peretz et al, 2016, 2018), Tuj1 was found to be predominantly absent from Sox2+ cells in the spheres, comprising approximately 20% of the total cells. However, a monitory of Tuj1+ cells in the sphere's periphery were also expressing Sox2 (see Fig.5E in the 2016 paper, and Fig.4A in the 2018 paper). Notably, as detailed in our reply to point 4, the co-expression of Sox2 and Tuj1 was also found in some of the hindbrain boundary cells in-vivo, as well as in the spinal cord.

In addition to the above clarifications, which have now been added to the text (pages 7-8), we have also beter evaluated the effect of ChABC on the molecular state of neurospheres, by adding two more markers, the early neurogenic marker DCX and the late differentiation marker MAP2, in addition to several more staining with Tuj1 (new Fig.4, Fig.9B,C, Fig.10E, new Fig. S8). We observed that the previously reported hierarchy is maintained in both the control and treated groups. Specifically, Sox2 cells disperse along the sphere, while early/late differentiation markers (Tuj1/Map2) are predominantly located at the periphery of the spheres, with litle presence in their core. Interestingly, we found that the immature neuronal marker DCX is not confined to the sphere periphery only. Nonetheless, we found that the main difference between the control and ChABC-treated cultures is evident in the formation of extending neurons, heavily formed upon loss of CSPG (as can be seen in Figs. 3,4,9,10). As we previously reported, these neurites that extend from the spheres are expressing DCX and Tuj1, while here we also found that they are positive to Map2. Quantification of DCX/Map2 in the cultures revealed a higher abundance of both markers in the ChABC-treated group, likely due to the increased presence of extending neurons, which were predominantly formed in this group. In contrast, DCX/Map2-positive cells in the control group probably represent the lower fraction of the positive cells located within the spheres. Furthermore, we observed a higher number of DCX+ cells in the control group, compared to Map2, which appears to result from the abundant formation of spheres where DCX is expressed throughout the structure, while Map2 is primarily localized to its periphery. In contrast, upon treatment, we found an equally high presence of both markers, demonstrating their co-expression in the extended neurites (Fig. S8). Finally, the revised version now includes more quantifications of the stained neurospheres in controls vs. ChABC groups, such as the percentage

© 2024. Published by The Company of Biologists under the terms of the Creative Commons Attribution License (https://creativecommons.org/licenses/by/4.0/).

of Sox2+/Map2+ cells in the CSPG+ derived spheres (Fig.9E) or Tuj1+ cells out of all DiI-labelled boundary cells (Fig. 10D).

We believe that these clarifications and additions provide a much-detailed characterization of the molecular/cellular composition of the neuropsheres in the multiple performed experiments.

9. Could they verify their transcriptomic findings with in situ hybridization stainings? Not all the genes, but a subset of the most interesting ones to check that indeed they are enriched or are specific of the hindbrain boundaries, and not only in the most dorsal domain along AP axis.

9. Reply: To address this request, we have performed multiple ISH or IF staining of DEGs in the CSPG+ and CSPG- groups (new Fig. 6); by examining multiple DEGs that were upregulated in the CSPG+ group (Dusp6, FGF3, FGF8, HSPG2, MMP2, LAmC1, MMP9, MMP16, Pax2, PTPRz1), their expression was found to be exclusively or mostly enriched at the boundaries, rather than in the dorsal hindbrain. Conversely, analysis of the RNAseq data of typical hindbrain dorsal markers (i.e., Wnt3a, Atoh1, Olig3, Lmx1, Msx1, Msx2, Lhx2, LhX9) has revealed their upregulation in CSPG- group, indicating that dorsal cells expressing these genes have not been collected with the CSPG+ group. ISH for two of these markers is also provided to show their exclusive dorsal expression (New Fig.6). Finally, examination of DEGs upregulated in the CSPG- group revealed their broad expression in the hindbrain, either being excluding/weaker at the boundaries (DLL1, LHX1/5, Pax7), or in domains of differentiating/differentiated neurons (ALAVL4, Isl1, Tuj1). Altogether, these results validate the sorting of the CSPG+ cell group to be specific to boundaries.

Reviewer 2-

1. The data presented in this study are comprehensive and of excellent quality. It would be nice to see an atempt to translate the CSPG gain of function experiments into embryos - is t possible to over-express or ectopically apply CSPGs in chick embryos and test how that influences the boundary cell type?

1. Reply: As suggested by the reviewer, we have ectopically added CSPG to the hindbrain lumen, and examined the expression of Sox2. While the results showed a tendency of the Sox2+ cells to become more confined at the boundaries upon excess CSPG, it was not statistically significant, so we have decided to not include it in the revised version. Here we provide an example of such a result (appended below). One way to explain the non-conclusive effect in vivo may be due to the fact that CSPGs are a group of proteins, mostly secreted, whereas the boundary specific CSPG is a membranal one, DSD1 (Fig. S1). The RNAseq data supported this finding by detecting various secreted CSPG to be upregulated in the CSPG-group, whereas DSD1 (termed PTPRz1 in the RNAseq) was upregulated in the CSPG+ group (Fig.5, new Fig.6). Hence, the strategy to add the soluble CSPG may not be very effective in recapitulating the role of the membranal-bound CSPG in the boundaries. The discrepancy between the lack of effect in vivo versus in vitro may be due the fact that the culture conditions positively select for the growth of NPSCs rather than neurons (Peretz et al., 2018), leading to a more uniform cell population in the dish that reacts to ectopic CSPG. Alternatively, as CSPG is a very large protein, it may have been washed away from the hindbrain lumen in vivo while persisted in the culture in vitro. Unfortunately, we couldn't get hold of a DSD1 plasmid to overexpress it in vivo. Nevertheless, as we think that loss-of-function experiments are very informative in uncovering a role of a gene, we have performed several new experiments that beter show the direct effect of CSPG-loss on the boundary/Sox2 cell fate and behavior (Fig.2F, new Fig. 4, new Fig.10)

NOTE: We have removed unpublished data that had been provided for the referees in confidence.

2. It also appears that a legend for Fig. 3 is entirely missing. In panel G of this figure, Tubb3 is analyzed but this gene is not mentioned in the main text.

2. Thank you for noticing these mistakes. We have now corrected this by explaining in the text that Tubb3 means Tuj1.

3. On page 9, it is mentioned that 'genes involved in neurogenesis, axonogenesis and

© 2024. Published by The Company of Biologists under the terms of the Creative Commons Attribution License (https://creativecommons.org/licenses/by/4.0/).

neuroectoderm induction [...] were notably downregulated in the CSPG+ group'. Neuroectoderm induction is a very early step in neural development that leads to the generation of proliferating neural progenitor cells which seems at odds with the notion that CSPGs maintain a stem cell-like state?

3. The referee is correct, as indeed some of the genes that were upregulated in the CSPG- groups are known from other studies to be involved in neural induction (such as Zic2/3). Yet, the same genes were also reported at later stages to be expressed during neural differentiation and circuitry formation. These evidences indicate the same gene has multifaceted roles that seem contradictory, yet, they take place at different time points and developmental processes. As these genes are upregulated together with many genes that are known to act during/after neural differentiation, we believe that also these genes have altered their roles to support these events. This is now noted in the text (page 11).

Reviewer 3-

1a. in vivo manipulations (Fig.3): The effect of CSPG decrease on Tuj1 is puzzling: first, Tuj1 seems to be enriched at HBs under physiological conditions (Fig.3E, top) while HBs should be largely composed of progenitor cells.

1a. Reply: This is a very important comment (see also reply 4 to referee 1), which we now beter clarify: The spatial expression of Sox2 and Tuj1 at the HBs was thoroughly addressed in our previous paper (Peretz et al., 2016); While in flat-mounted overviews they seem to co-localize at the HBs, when analyzing the Z axis, Sox2+ cells are shown to localize at the ventricular/sub-ventricular zone, whereas Tuj1+ cells/axons are mostly enriched in in the mantle layer (the enrichment of the boundary's mantle layer with axonal fibers was actually already reported in Heyman et al., 1995. Moreover, we have also GFP-labelled and traced the HB-Sox2 cells in our previous study, and demonstrated that the differentiating Tuj1+ neurons which migrate to the HBs-mantle zone, originate from Sox2+ cells in the HBs ventricular zone. Hence, we have concluded that the HBs' population is comprised of progenitor cells (Sox2+) in the ventricular zone that give rise to differentiating cells, which migrate towards the mantle zone and upregulate Tuj1/3A10/HuC/D (Peretz et al., 2016). Notably, our previous analysis also revealed that some of the radially migrating Sox2+ cells begin to upregulate Tuj1 before downregulating Sox2, whereas others upregulate Tuj1 only after reaching the mantle layer and losing Sox2 expression.

Yet, to beter clarify this point in the present manuscript, we conducted staining in chick and mouse hindbrains, capturing images of the tissue in whole-mounted views and transverse sections. Our analysis reveals that in both species, Sox2+ cells are present in the ventricular and subventricular layers of the HBs but not in the mantle region, while Tuj1 is predominantly localized in the mantle zone. However, some Sox2+/Tuj1+ cells are also observed more apically, consistent with our previous results and similar findings in the chick spinal cord (Peretz et al., 2016, 2018, Das and Storey, 2014). These data are now presented in new Fig. S5 and in the text (page 6). Moreover, we have now also traced boundary cells by electroporating them with control-GFP or ChABC-GFP plasmids and showed their gradual radial migration towards the pial surface, together with their upregulation of Tuj1 and tangential migration in the mantle zone (Fig. 2F, H, Fig. S6C,D, as also noted in reply XXXX below). Finally, these expression paterns have been also verified by our RNAseq data, which shows a differential expression of *Sox*2 or *Tuj1* and Huc/D in the CSGP+ or CSPG- groups, respectively (Fig. 5, new Fig.6).

1b. Second, Tuj1 induction upon ChABC seems to occur primarily inside compartments as opposed to HBs (Fig.3E, botom). Can the authors explain these surprising observations?

Ib. Reply: We thank the reviewer for this important observation, which can be explained by two scenarios (see also reply 5 to reviewer 1); first, as shown in Fig.1, new Fig. S2, as well as in Peretz et al., 2016, although CSPG+/Sox2+ cells are mostly enriched at the boundaries, there are fewer Sox2+/CSPG+ in the rhombomeres as well, which may have also been affected by ChABC, leading to their enhanced differentiation in these sites. Another possibility is based on our previous study, where we have reported that while most of the HB/sox2+ cells are slow-dividing, some rapid- amplifying Sox2 cells exist near the boundary-rhombomere interphase and are being contributed to the rhombomeres and they undergo differentiation (Peretz et al, 2016). Therefore, it is also possible

© 2024. Published by The Company of Biologists under the terms of the Creative Commons Attribution License (https://creativecommons.org/licenses/by/4.0/).

that upon CSPG-loss, more boundary cells become fast-amplifying progenitors that arrive to the rhombomeres, resulting in the enhanced differentiation of the boundary-derived Sox2 cells in the rhombomeres. We have preliminary evidence which support this possibility (see also reply 7 to Reviewer 1), and have now added a paragraph that discussed this observation (page 18). Moreover, we have performed new experiments to trace individual HB cells in vivo to beter observe the local/global effect of CSPG-loss (detailed in the paragraph below).

2. HB cells would need to be traced to monitor their progenitor/differentiation status at the individual cell level upon ChABC treatment. Do individual Sox2+ cells convert to differentiation and TuJ1 expression? Can the authors rule out cell migration? Is the fate of individual compartment cells unchanged?

2. Reply: As suggested by the referee, we have performed electroporation with control-GFP or ChABC-GFP plasmids and analyzed the GFP-expressing Sox2+ cells in sections; higher number of radially migrating GFP+ cells that move towards the basal side, downregulate Sox2, upregulate Tuj1 and extend axons were observed in the ChABC-expressing -boundary cells compared to controls. This data, now presented in Fig. 2F,H and Fig. S6C, indicate that individual boundary cells change their fate towards differentiation upon losing CSPG. This experiment has also been conducted in vitro (new Fig. 4, Fig. S10, Movies 7-8), showing the same results. Regarding the comment on cell migration, we wish to note that, since radial migration of progenitors is coupled with neural differentiation in the neural tube, we cannot dissociate between these two features. Nevertheless, as the basally-migrating cells which express ChABC are upregulating Tuj1 and downregulating Sox2 (Fig. 2F,H and Fig. S6C), the possibility that the CSPG-excluded cells migrate basally without switching to differentiation mode is less likely.

Regarding the compartments, we have used a pan-rhombomere marker, HNK1 (Peretz et al., 2016), and found that the robust segmental organization of the hindbrain has not been modified upon CSPG loss (figure appended below, and mentioned in the text (page 6)). We have not examined specific Rhs markers, as both our work and previous studies have demonstrated that manipulations of chick hindbrain boundaries at the stages we investigate, considerably beyond the time when compartment identities are established, do not influence compartment identities, which appear to be highly stable in the tissue (Guthrie and Lumsden, 1991, Nitenberg et al., 1997, Weisinger et al., 2008, 2012). Hence, since CSPG/Sox2 cells are found in all boundaries, we suggest that the boundaries serve as local reservoirs of progenitors maintained by CSPG between any two rhombomeres, regardless of the later's identities.

NOTE: We have removed unpublished data that had been provided for the referees in confidence.

3. in vitro manipulations (Figs.8): given that the cultures are not pure (4 times enrichment for CSPG+ cells only), Sox2+ cells need to be individually traced to monitor their fate and ensure that excess Tuj1 upon ChABC is indeed due to the differentiation of previously Sox2+ cells (as opposed to independent increase in neuritogenesis, for example).

3. Reply: Again, we thank the reviewer for this important comment. The best manner to track Sox2+ boundary cells is by lineage tracing. We have tried that by two different approaches: (i) electroporating different Sox2-GFP enhancers in the chick (Uchikawa et al. 2003) or (ii) using Sox2CreER; R26RmTmG mice to induce Sox2 expression upon Tamoxifen injection (Du et al., 2017). In both cases, GFP expression was leaky and not confined to the boundaries only, preventing us from being able to specifically trace the boundary-Sox2 cells. The GFP-leakiness could have been anticipated due to the dynamic nature of Sox2 expression in the hindbrain, initially broadly expressed in rhombomeres, then gradually downregulated, and maintained in the boundaries (new Fig.S2, Peretz et al., 2016), preventing the ability to exclusively trace boundary-Sox2 by these genetic approaches. Therefore, we decide to take advantage of the chick embryo's ability to be manually labeled in-ovo. As the vast majority of the cells in the boundaries' ventricular layer express Sox2 and CSPG (Fig. 1, Peretz et al 2016), we have microinjected DiI in vivo to a portion of these cells, made primary cultures and live-traced the DiI+ cells with and without CSPG (new Fig. 10. Fig. S13, movies 13,14). Most of the control DiI+ cells remained as part of the rounded neurospheres. In contrast, many of the ChABC treated DiI+ cells left the spheres, flatened and extended neurites. Stanning for Sox2/Tuj1 clearly showed the loss of Sox2 and induction of

© 2024. Published by The Company of Biologists under the terms of the Creative Commons Attribution License (https://creativecommons.org/licenses/by/4.0/).

Tuj1 in the DiI+ ChABC treated cells, as opposed to the control. This experiment confirms that the HB/Sox2+ cells are shiftiing towards differentiation when CSPG is inhibited, as we found in the CSPG+ purified cultures. In addition, as requested by reviewer 1 (see reply 5), we have also performed electroporations with control-GFP or ChABC-GFP plasmids and live- traced the cells in vitro (new Fig. 4, Fig. S10, movies 7,8). ChABC-expressing cells displayed a marked neural differentiation morphology of cells that left the sphere, downregulated Sox2 and extended Tuj1+ neurites, in contrast to control-GFP cells that remained within the sphere and expressed Sox2.

Finally, we wish to note that we have improved the immunocoloum protocol for separating the CSPG+ cells, achieving a much higher level of enrichment of the CSPG+ group (>15 fold increase, data not shown). In this case, the behavior of CSPG+ vs. CSPG- cells was consistent with the culture presented in the manuscript (data not shown), aligning with our earlier study demonstrating the selective growth conditions favoring progenitor cells over neurons (Peretz et al., 2018). Consequently, even in a more mixed cell culture, the dish is enriched with Sox2+/CSPG+ progenitors.

List of References:

Das RM, Storey KG. Apical abscission alters cell polarity and dismantles the primary cilium during neurogenesis. Science. 2014 Jan 10;343(6167):200-4. doi: 10.1126/science.1247521. PMID: 24408437; PMCID: PMC4066580.

Du, W., Hu, J. K.-H., Du, W. and Klein, O. D. (2017). Lineage tracing of epithelial cells in developing teeth reveals two strategies for building signaling centers. *Journal of Biological Chemistry* 292, 15062–15069.

Guthrie, Sarah, and Andrew Lumsden. 1991. “Formation and Regeneration of Rhombomere Boundaries in the Developing Chick Hindbrain.” *Development (Cambridge, England)* 112(1): 221–29.

Heyman, I., Faissner, A. and Lumsden, A. (1995). Cell and matrix specialisations of rhombomere boundaries. *Developmental Dynamics* 204, 301–315.

Kohl A, Hadas Y, Klar A, Sela-Donenfeld D. Axonal paterns and targets of dA1 interneurons in the chick hindbrain. J Neurosci. 2012 Apr 25;32(17):5757-71. doi: 10.1523/JNEUROSCI.4231-11.2012. PMID: 22539838; PMCID: PMC6703607.

Kohl A, Marquardt T, Klar A, Sela-Donenfeld D. Control of axon guidance and neurotransmiter phenotype of dB1 hindbrain interneurons by Lim-HD code. J Neurosci. 2015 Feb 11;35(6):2596-611. doi: 10.1523/JNEUROSCI.2699-14.2015. PMID: 25673852; PMCID: PMC6605615.

Nitenberg R, Patel K, Joshi Y, Krumlauf R, Wilkinson DG, Brickell PM, Tickle C, Clarke JD. Cell movements, neuronal organisation and gene expression in hindbrains lacking morphological boundaries. Development. 1997 Jun;124(12):2297-306. doi: 10.1242/dev.124.12.2297. PMID: 9199356.

Peretz, Y., Eren, N., Kohl, A., Hen, G., Yaniv, K., Weisinger, K., Cinnamon, Y. and Sela-Donenfeld, D. (2016). A new role of hindbrain boundaries as pools of neural stem/progenitor cells regulated by Sox2. *BMC Biol.* 14, 1–20.

Peretz, Y., Kohl, A., Slutsky, N., Komlos, M., Varshavsky, S. and Sela-Donenfeld, D. (2018). Neural stem cells deriving from chick embryonic hindbrain recapitulate hindbrain development in culture. *Sci. Rep.* 8, 1–15.

Uchikawa, Masanori, Yoshiko Ishida, Tatsuya Takemoto, Yusuke Kamachi, and Hisato Kondoh. 2003. “Functional Analysis of Chicken Sox2 Enhancers Highlights an Array of Diverse Regulatory Elements That Are Conserved in Mammals.” *Developmental cell* 4(4): 509–19.

Weisinger, Karen, Galya Kayam, Talya Missulawin-Drillman, and Dalit Sela-Donenfeld. 2010. “Analysis of Expression and Function of FGF-MAPK Signaling Components in the Hindbrain

© 2024. Published by The Company of Biologists under the terms of the Creative Commons Attribution License (https://creativecommons.org/licenses/by/4.0/).

Reveals a Central Role for FGF3 in the Regulation of Krox20, Mediated by Pea3." *Developmental biology* 344(2): 881– 95.

Weisinger, Karen, Ayelet Kohl, Galya Kayam, Efrat Monsonego-Ornan, and Dalit Sela-Donenfeld. 2012. "Expression of Hindbrain Boundary Markers Is Regulated by FGF3." *Biology open* 1(2): 67–74.

Second decision letter

MS ID#: DEVELOP/2023/201934

MS TITLE: Hindbrain boundaries as niches of neural progenitor/stem cells regulated by the extracellular matrix proteoglycan chondroitin sulphate.

AUTHORS: Carmel Hutchings, Yarden Nuriel, Daniel Lazar, Ayelet Kohl, Olga Genin, Yuval Cinnamon, Elizabeth Muir, Hadar Benyamini, Yuval Nevo, and Dalit Sela-Donenfeld
ARTICLE TYPE: Research Article

I am happy to tell you that your manuscript has been accepted for publication in Development, pending our standard ethics checks.

Reviewer 1

Advance summary and potential significance to field

The article from Hutchings et al explores further how Sox2-expressing boundary cells behave as neural progenitors. Previously, the same lab had described that hindbrain boundary cells in the chick embryo express Sox2 and the proteoglycan CSPG. They showed that the majority of the Sox2-cells within the boundary core slow-divided, and they could form neurospheres in culture (Peretz et al, 2016).
Here, they show that Sox2-boundary cells do display CSPG, both in chick and mice embryos and several functional experiments suggest that CSPG maintains Sox2 boundary cells in the progenitor state. Transcriptomic analyses of CSPG-sorted boundary cells reveal they express neural progenitor genes. The authors state that CSPG maintains boundary cells in the undifferentiated state.

Comments for the author

The authors have done a great effort to address all raised issues and comments.
Currently, the manuscript shows that CSPG is crucial to maintain boundary cells undifferentiated in amniotes and brings important findings such as the transcriptomic analyses of boundary cells.

Reviewer 3

Advance summary and potential significance to field

These authors have made an extensive and careful revision. This version of the work includes important new data and is overall more conclusive. I feel that the revisions satisfactorily address my specific concerns. In particular, the DiI tracing data presented in the new Fig.10 bring us one step further in the demonstration of cell fate changes (from the Sox2+ to the TuJ+ state) when blocking CSPG.

Comments for the author

I thank the authors for their efforts and I am ready to accept this revised version of the manuscript.

© 2024. Published by The Company of Biologists under the terms of the Creative Commons Attribution License (https://creativecommons.org/licenses/by/4.0/).
